# Copper (triazole-5-yl)methanamine complexes onto MCM-41: the synthesis of pyridine-containing pseudopeptides through the 6-*endo*-dig cyclization of 1,5-enynes†

**DOI:** 10.1039/c9ra10885h

**Published:** 2020-03-12

**Authors:** Neda Akbarikalani, Kamran Amiri, Ahmed Al-Harrasi, Saeed Balalaie

**Affiliations:** Peptide Chemistry Research Center, K. N. Toosi University of Technology P. O. Box 15875-4416 Tehran Iran balalaie@kntu.ac.ir +98-21-22889403 +98-21-23064226; Natural and Medical Sciences Research Center, University of Nizwa P. O. Box 33, Postal Code 616, Birkat Al Mauz Nizwa Sultanate of Oman; Medical Biology Research Center, Kermanshah University of Medical Sciences Kermanshah Iran

## Abstract

An efficient approach for the synthesis of immobilized copper (triazole-5-yl)methanamine complexes onto MCM-41 (Cu@TZMA@MCM-41), as a novel recyclable nanocatalyst, is described. This nanocatalyst was used for the synthesis of pyridine-containing pseudopeptides through a sequential Ugi/nucleophilic addition/1,5-enyne cyclization reaction and elicited good-to-excellent yields. The nanocatalyst was fully characterized by SEM, EDS, TEM, BET, ICP-OES, TGA, and XRD techniques. Furthermore, the catalyst was recovered by simple filtration and could be used for at least 5 cycles without significant loss of activity.

## Abstract 2

A sequential Ugi/nucleophilic addition/1,5-enyne cyclization reaction was used for the synthesis of pseudopeptides containing pyridine skeletons in the presence of Cu@TZMA@MCM-41.

## Graphical abstract

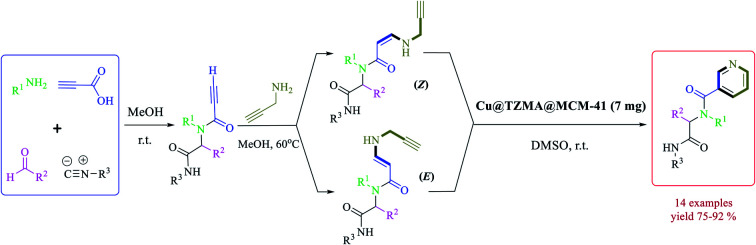

## Introduction

**Fig. 1:**
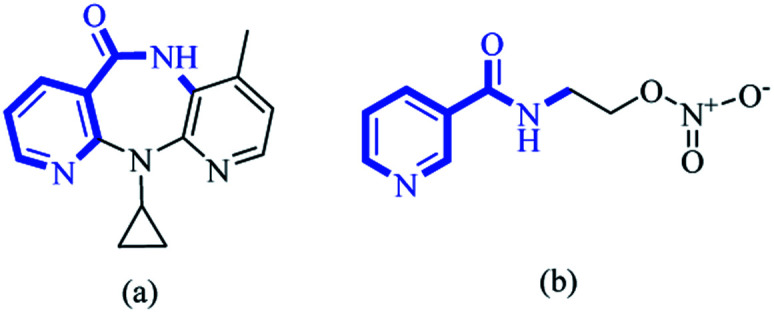
The structures of (a) nevirapine and (b) nicorandil.

Over recent decades, selection of functionalized building blocks has allowed chemists to design novel “domino” or sequential reactions to access complex molecules. As bifunctional compounds, 1,*n*-enynes (particularly 1,5-enynes) have emerged as attractive moieties for generating various cyclic skeletons, such as 1-aza anthraquinones,^1^ 1,4-oxazepine,^2^ bicyclo [3.1.0] hexane,^3^ cyclohexadienes,^4^ pyrido[4,3,2-*mn*] acridin-8-one,^5^ furans,^6^ dihydropyrans^7^ and cyclohex-4-ene-1,2-diol.^8,9^ The pyridine backbone is a well-known heterocyclic skeleton in bioactive and pharmaceutical compounds, with biological activities.^10–17^ For instance, nevirapine and nicorandil show antiviral and antianginal activities, respectively (Fig. 1).

In recent years, depending on the structure of the starting 1,*n*-enynes, reaction conditions, and different types of metal catalysts (*e.g.*, Pt,^18^ Au,^7,19^ Cu,^20^ Fe,^21^ Bi,^22^ Rh^23^ and Ag^24^), various nitrogen-containing heterocyclic compounds have been synthesized.^25^

Nevertheless, the separation and recycling of homogenous metal catalysts from the product is a major drawback limiting wider application of these compounds.

The design of efficient and reusable heterogeneous catalysts (particularly for C–C and C-heteroatom coupling reactions) has become important.^26^ However, support of homogeneous catalysts on various solid supports is disadvantageous due to the reduction in catalytic activity. Hence, strategies have focused on using nanoparticles as ideal and efficient heterogeneous supports.^27^

Among various nanomaterials, mesoporous silica (*e.g.*, MCM-41) due to having nano-sized pores, high pore volume, specific surface area, non-toxic content, and good thermal and mechanical stability, has been employed as a support.

Meanwhile, several MCM-41-supported copper complexes have been reported and used as heterogeneous catalysts in organic synthesis.^28^

The design of sequential multiple components and cyclization reactions are powerful approaches for the synthesis of structurally complex and functionally diverse heterocyclic compounds.^29^ Recently, the Ugi post-transformation reaction has been shown to be a powerful reaction for facile synthesis of novel heterocyclic skeletons under simple and mild reaction conditions that could open up new areas in drug synthesis.^30^ Meanwhile, this method is known for the synthesis of multi-functionalized compounds.

**Scheme 1:**
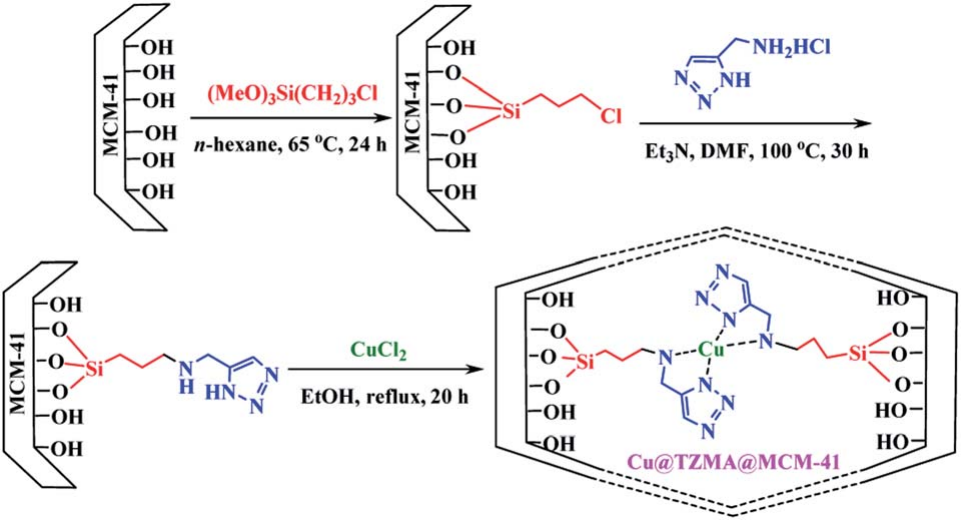
The synthesis of copper (triazole-5-yl)methanamine complexes onto MCM-41 (Cu@TZMA@MCM-41).

**Scheme 2:**
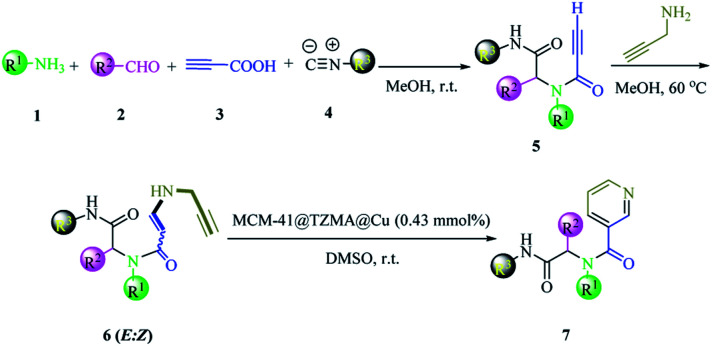
The synthesis of pyridine-containing pseudopeptide backbone derivatives in the presence of Cu@TZMA@MCM-41 through a sequential Ugi/nucleophilic addition/1,5-enyne cyclization reaction.

We have a strong interest in designing post-transformation reactions.^31^ Herein, we report an efficient, mild and facile procedure to provide 1,5-enynes and design of novel cyclization reactions in the presence of a new nano heterogeneous catalyst for the synthesis of a pyridine skeleton. We introduced a new strategy for the synthesis of a pyridine-containing pseudopeptides backbone through sequential Ugi/nucleophilic addition/1,5-enyne cyclization reaction. We immobilized Cu (triazole-5-yl)methanamine complexes onto MCM-41 (Cu@TZMA@MCM-41) as a novel nanocatalyst (Scheme 1 and 2).

## Results and discussion

To access a novel reusable nanocatalyst (Cu@TZMA@MCM-41), initially MCM-41 was prepared through a sol–gel method (Scheme 1). Subsequently, MCM-41 was functionalized using 3-chloropropyltrimethoxysilane, and then (triazole-5-yl) methanamine was grafted as a bidentate ligand. Finally, immobilization of copper on the surface TZMA@MCM-41 led to the formation of Cu@TZMA@MCM-41. Scanning electron microscopy (SEM), transmission electron microscopy (TEM), Brunauer–Emmett–Teller (BET) theory, thermogravimetric analysis (TGA), X-ray powder diffraction (XRD) spectroscopy, inductively coupled plasma atomic emission spectroscopy (ICP-OES) and energy-dispersive X-ray (EDX) spectroscopy were used to characterize the structure of the catalyst.

**Fig. 2:**
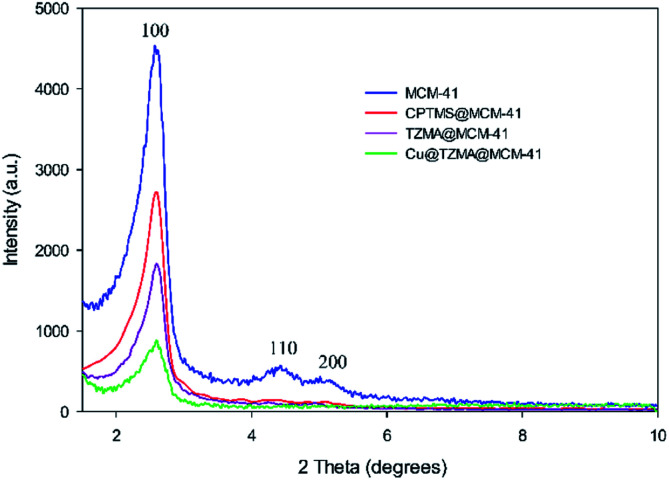
XRD patterns of MCM-41 (blue curve), CPTMS@MCM-41 (red curve), TZMA@MCM-4 (violet curve) and Cu@TZMA@MCM-41 (green curve).

The XRD spectroscopy patterns at a low angle of the MCM-41 support and supported catalysts are illustrated in Fig. 2. The diffractograms of samples exhibited three peaks: an intense diffraction peak for the d_100_ plane at 2*θ* = 2.58° and two weak diffractions for d_110_ and d_200_ planes at 2*θ* = 4.41 and 5.13°, respectively. After functionalization of MCM-41 channels, peak intensities were decreased, which confirmed incorporation of the copper complex onto MCM-41 channels and that the structural integrity of the mesoporous had been retained.

**Fig. 3:**
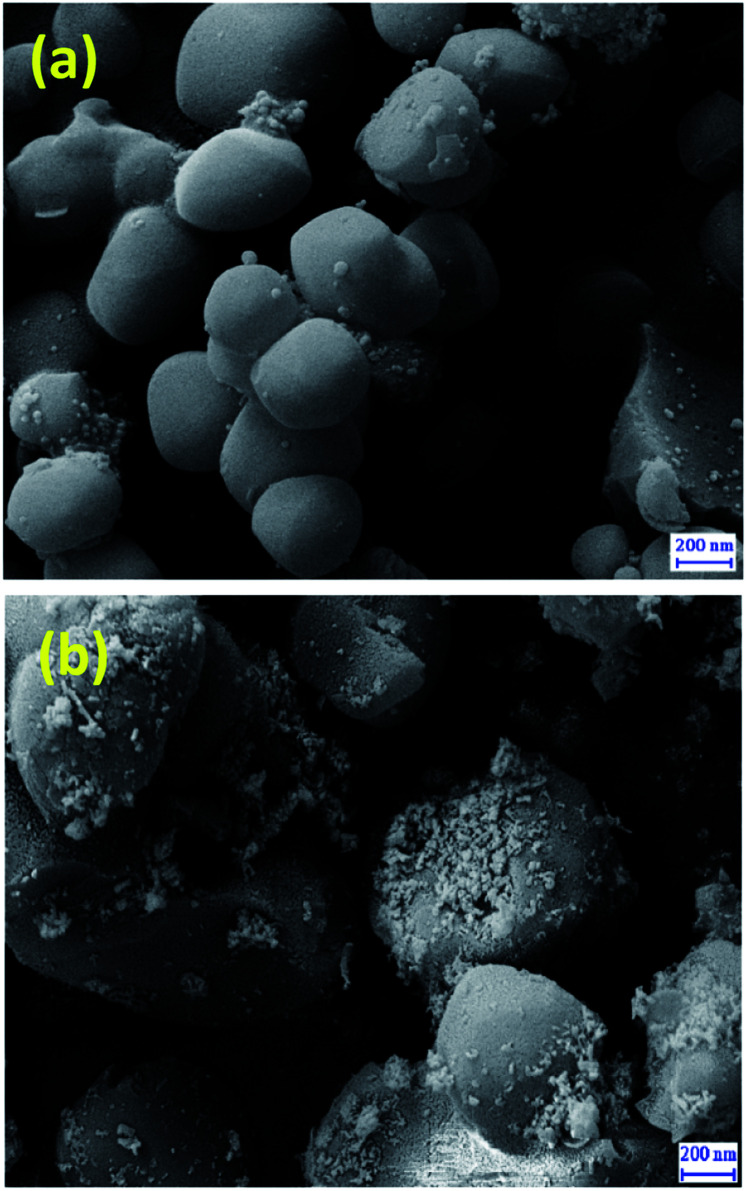
FE-SEM images of MCM-41 (a) and Cu@TZMA@MCM-41 (b).

SEM images of MCM-41 (Fig. 3a) and Cu@TZMA@MCM-41 (Fig. 3b) demonstrated that these catalysts had been formed as spheres. Furthermore, upon functionalization of MCM-41, the surface morphology of the nanocatalyst was not changed significantly.

**Fig. 4:**
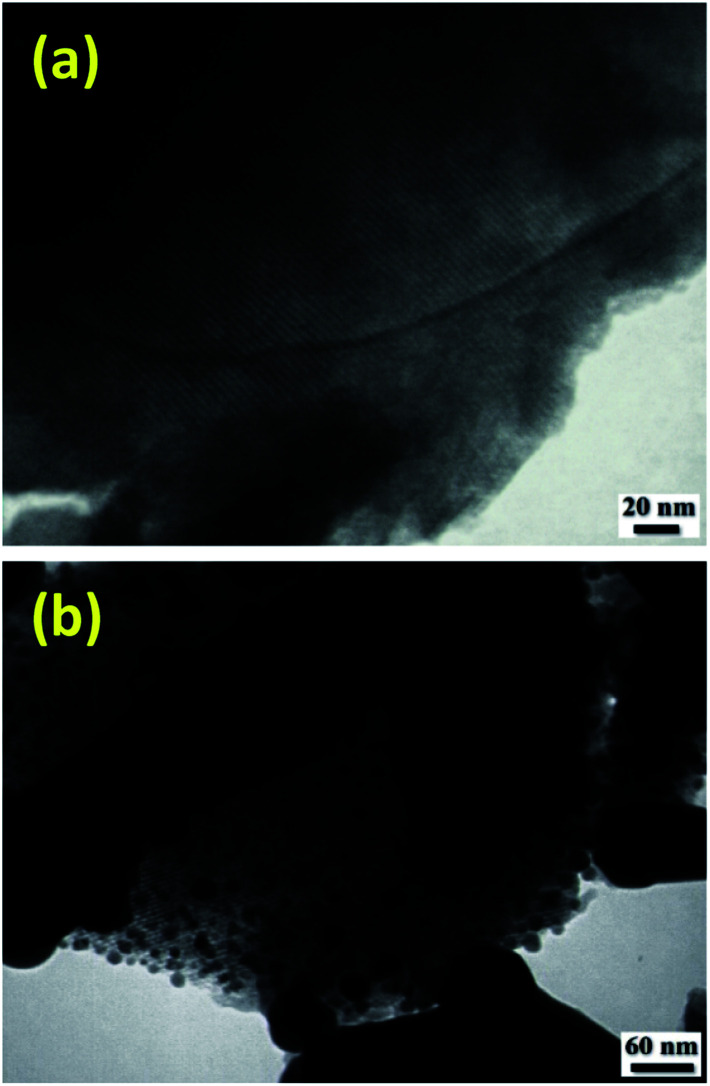
TEM images of MCM-41 (a) and Cu@TZMA@MCM-41 (b).

TEM images of some of the samples clearly illustrated the well-ordered arrangement of pores. As shown in Fig. 4, copper grafting did not change the periodicity of the hexagonally mesoporous structure.

**Fig. 5:**
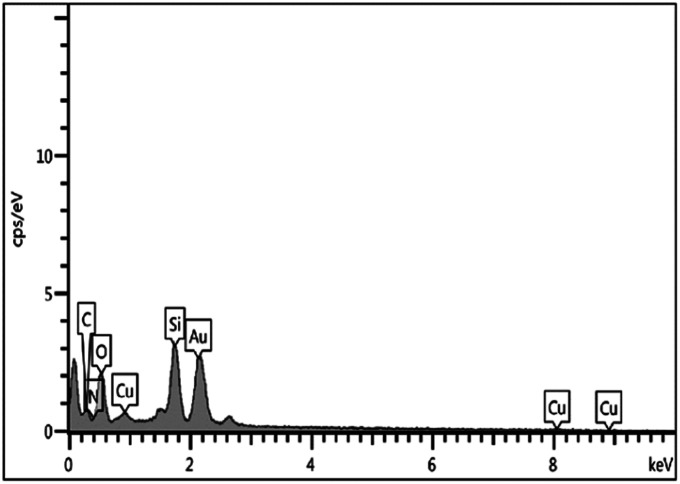
EDX spectrum of Cu@TZMA@MCM-41.

The EDX spectrum indicated support of copper particles onto the MCM-41 surface (Fig. 5). Furthermore, ICP-OES was used to ascertain the percentage of copper particles immobilized on the mesoporous catalyst. The loading amount of copper in the catalyst was found to be 3.9%.

**Fig. 6:**
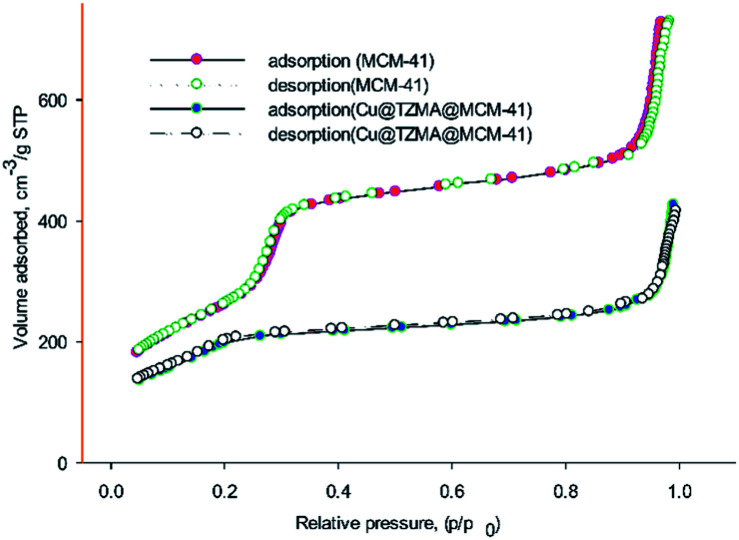
Nitrogen adsorption–desorption curves of MCM-41 and Cu@TZMA@MCM-41.

The textural parameters of MCM-41 and Cu@TZMA@MCM-41 were investigated using nitrogen adsorption–desorption (Fig. 6). Based on the IUPAC classification, both samples demonstrated type-IV isothermal curves, which are related to mesoporous structures. After functionalization of the pores of MCM-41, the pore volume, surface area and wall diameter of MCM-41 and Cu@TZMA@MCM-41 was found to be 1.3 and 0.8 cm^3^ g^−1^, 985 and 470 m^2^ g^−1^, and 1.06 and 2.81 nm, respectively. Hence, the total pore volume and specific surface area were decreased, and the wall diameter was increased. These data were attributed to grafted organic moieties and copper on pore channels.

**Fig. 7:**
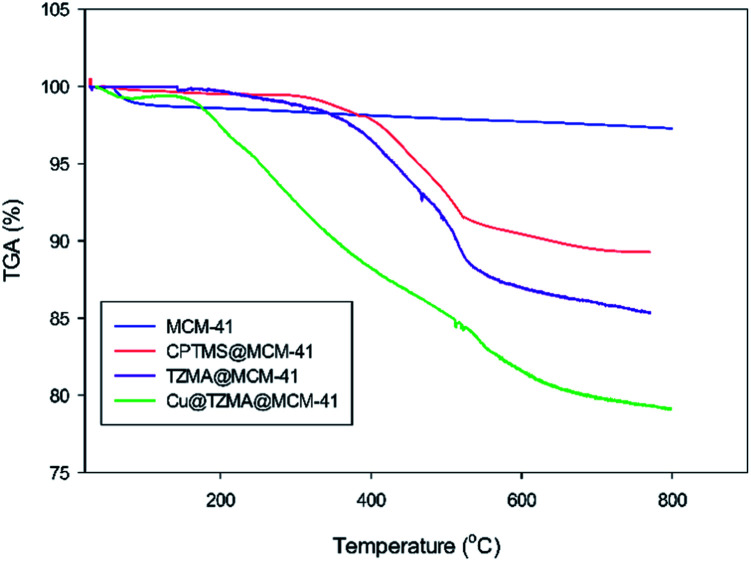
TGA curves of MCM-41, CPTMS@MCM-41, TZMA@MCM-4 and Cu@TZMA@MCM-41.

Thermograms were used to determine the weight changes of catalysts before and after modification of synthesized mesoporous silica (Fig. 7). The first step of weight loss (at <200 °C) corresponded to removal of physically absorbed water and organic solvents. The next step of weight loss occurred upon increasing the temperature to 800 °C: this was related to decomposition of immobilized organically modified moieties and silanol groups.

After nanocatalyst generation, we attempted the synthesis of new functionalized 1,5-enynes. To achieve this goal, in the initial study, the Ugi 4-CR of 4-nitroaniline 1h, benzaldehyde 2h, propiolic acid 3h, and cyclohexyl isocyanide 4h in MeOH at room temperature was selected as a model reaction. In the structure of the product 5h, there was an alkyne moiety that had high potential for nucleophilic addition. Then, propargylamine was added to the crude Ugi-adduct 5h in the same reaction vessel. There were two possibilities for the nucleophilic addition of propargylamine to an alkyne moiety that led to the synthesis of two diastereomers of 6h (*E* : *Z* 78 : 22). The ratio of the isomers was ascertained using ^1^H nuclear magnetic resonance (NMR) spectroscopy.

Finally, without separating 1,5-enyne 6h, the solvent was evaporated and then the reaction was investigated for 6-*endo*-dig cyclization in the presence of various amounts of nanocatalysts (Table 1, entries 2–5). In comparison with other amounts of catalysts, 7 mg (0.43 mmol%) of catalyst gave a better result (Table 1, entry 4). When the amount of nanocatalyst was increased to 9 mg, no change was observed in the yield of the product (Table 1, entry 5). Also, the experiment did not occur without a catalyst (Table 1, entry 1). Various solvents were screened (Table 1, entries 6–11). It appeared that dimethyl sulfoxide (DMSO) was the most suitable solvent for the final step of the reaction (Table 1, entry 4). Also, comparison of the activity of Cu@TZMA@MCM-41 and Cu/C in the model reaction was investigated: the yield of the desired product in the presence of Cu/C was only 50%.

**Table 1:** Optimization of the reaction conditions for the synthesis of 7h^a^

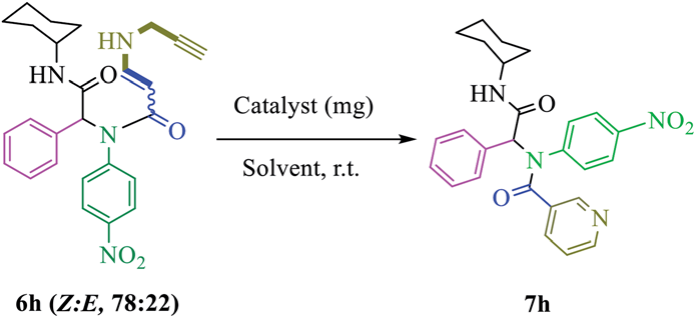			
Entry	Catalyst (mg)	Solvent	Yield (%)
1	—	DMSO	—
2	3	DMSO	76
3	5	DMSO	83
4	7^b^	DMSO	92
5	9	DMSO	92
6	7	DMF	85^c^
7	7	PEG	70
8	7	MeOH	Trace
9	7	EtOH	—
10	7	Dioxane	—
11	7	DCE	15
12	7	MeOH-DMSO	45

a Duration of the reaction was 3 h.

b 7 mg = 0.43 mmol%.

c Duration of the reaction was 4.5 h.

Based on observations stated above, we evaluated the scope and limitations of this newly developed protocol. Hence, various Ugi adducts were synthesized from propiolic acid and several aniline derivatives, benzaldehydes, and isocyanides, and subjected to the sequential Ugi-4CR/intermolecular nucleophilic addition/cyclization reaction in the presence of Cu@TZMA@MCM-41 at high yields (Table 2).

**Table 2:** The substrate scope for the synthesis of the pyridine-containing pseudopeptides 7a–n

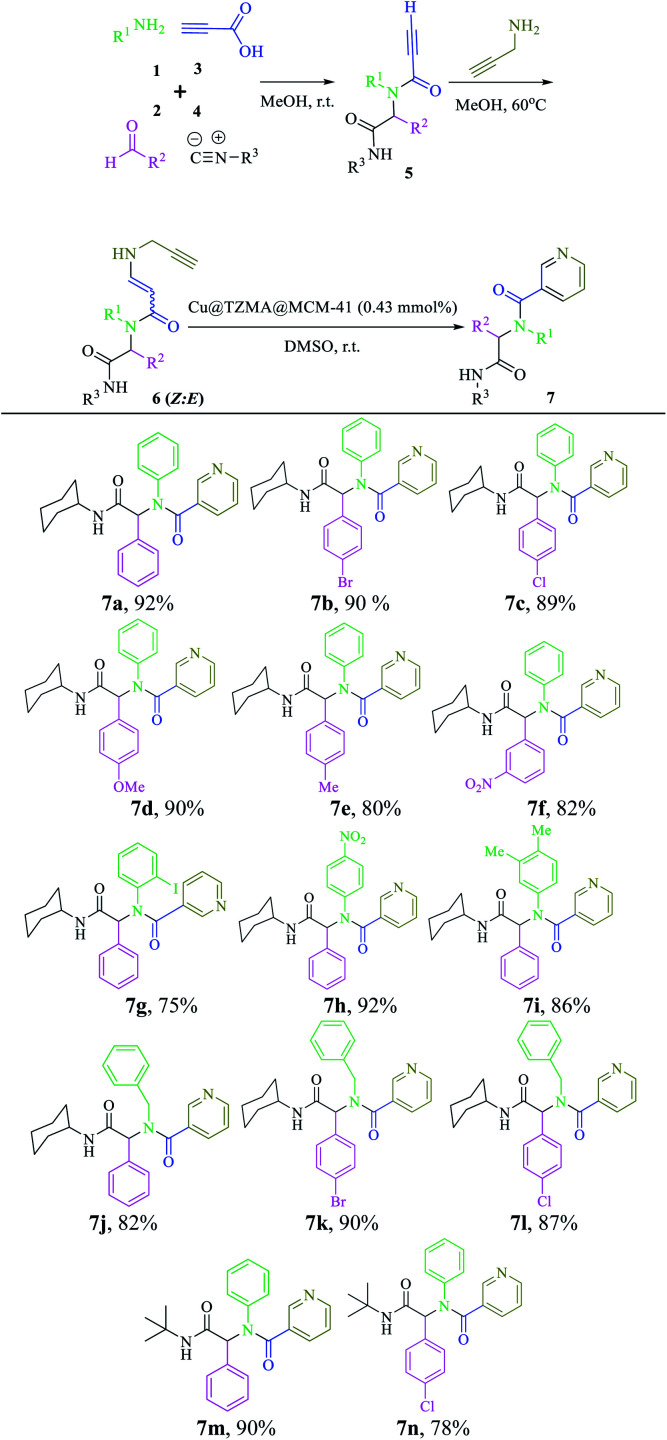

The structures of three compounds, 6b, h and n, were selected and determined by spectroscopy (Fourier transform-infrared (FT-IR), ^1^H NMR, ^13^C NMR). The ratio of the *E* : *Z* isomers was determined based on their ^1^H NMR spectral data. In cases 6b, h and n, the ratio of *E* : *Z* isomers was 74 : 26, 78 : 22 and 85 : 15, respectively. The presence of bonds at 1671, 2241 and 3293 cm^−1^ was attributed to (C=O), (C≡C) and (N–H), respectively. The ^1^H NMR spectrum of 6h (*Z*) and (*E*) included a mixture of two diastereomers. One of them was a doublet for H_a_-vinyl (*Z*) at *δ* = 4.07 ppm with *J* = 8.2 Hz, and the other one had a doublet for H_a_-vinyl (*E*) at *δ* = 4.43 ppm with *J* = 12.8 Hz. Formation of final products 7 was confirmed by spectroscopy. In the FT-IR spectrum of 7h, absorption at 2241 cm^−1^ was related to deletion of C≡C bonds. The absence of the acetylenic H at *δ* = 2.15 and 2.28 ppm and presence of a singlet peak for H-pyridine at *δ* = 8.48 ppm in the ^1^H NMR spectrum and, moreover, the presence of C-pyridine at *δ* = 150.7 ppm and absence of the acetylenic carbon at *δ* = 85.2 and 79.2 ppm, revealed the 6-*endo*-dig cyclization reaction.

**Scheme 3:**
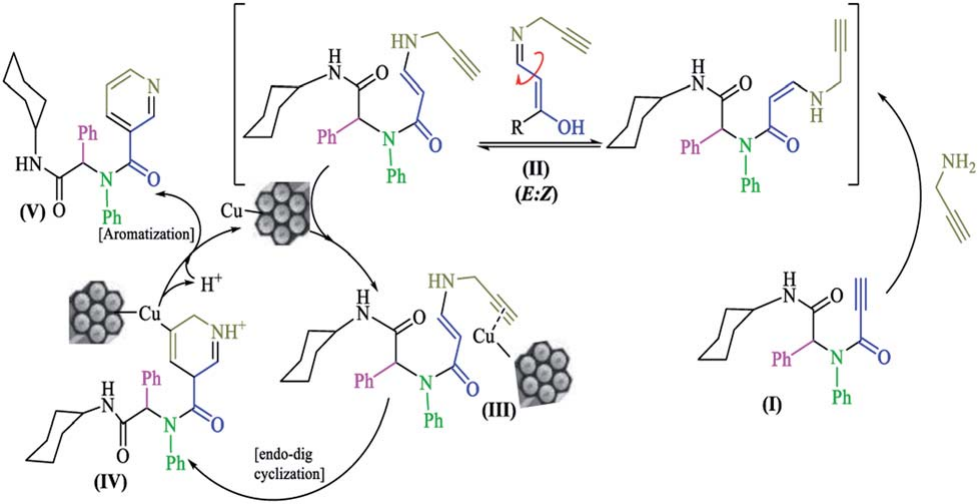
The proposed mechanism for the synthesis of 7a–n.

On the basis of work by Abbiati and colleagues,^32^ a plausible reaction mechanism is shown in Scheme 3. The *N*-substituted-2-alkynamide intermediate (I) had high affinity towards intramolecular nucleophilic addition. Propargylamine was added to activate the triple bond to furnish a mixture of two diastereomers (*Z* and *E*) *N*-propargylic enamide; during the reaction, the *Z* diastereomer was converted to the *E* diastereomer (II). The copper nanocatalyst coordinated with the new terminal triple bond, rendering it active for nucleophilic attack (III). Subsequently by attack of the carbon α-position and 6-*endo*-dig cyclization, a six-membered ring intermediate (IV) was formed, which aromatized to pyridine (V) *via* oxidation by air and regeneration of the copper catalyst.

Simple recovery and recyclability are valuable properties of catalysts. Therefore, the reusability of the catalyst was studied in the 6-*endo*-dig cyclization reaction of 1,5-enynes. Hence, after completion of the reaction, the nanocatalyst was easily separated *via* filtration and washed several times with EtOAc and reused directly in the next runs (up to five times) using the same process without a change in catalytic activity. The exact amount of copper was measured by ICP-EOS, and showed that the amount of copper had decreased from 3.9% at the first run to 3.8% after five recycles.

## Experimental section

### General remarks

The reagents and solvents used in this project were purchased from various chemical companies and were used directly without additional purification. The reactions were monitored by thin-layer chromatography (TLC) over gel-60 F_254_ plates. Chromatography columns were packed using 63–200 mesh ASTM silica gel. Melting points were measured *via* an electrothermal 9100 apparatus. FT-IR spectroscopy was done using KBr disks in a AABFT-IR (FTLA 2000) spectrophotometer. ^1^H NMR (300 and 600) and ^13^C NMR (75 MHz) spectra were recorded with a Bruker spectrometer. High-resolution mass spectra of the products were recorded with an Agilent quadrupole time-of-flight liquid chromatography/mass spectrometry system. XRD spectroscopy patterns of as-prepared samples were obtained by an XPert Pro Panalytical setup. The morphology and size of particles were recorded using a scanning electron microscope (Zeiss-SIGMA VP). TEM images were obtained by a Zeiss EM10C (100 kV) system. The specific surface area and pore size of the synthesized nano-catalyst were determined by N_2_ sorption–desorption. The TGA curves of samples were obtained using a Shimadzu DTG-60 instrument. The copper content of the nanocatalyst was determined ICP-OES. The elemental analysis of the catalyst was achieved by EDX using a Zeiss SIGMA VP system.

### Preparation of ligand ((triazole-5-yl)methanamine)

To a stirring solution of phthalimide (3.4 mmol) and K_2_CO_3_ (5.1 mmol) in CH_3_CN (12 mL) was added propargyl bromide (5.8 mmol) under reflux. After 48 h, the hot mixture was filtered and cooled at room temperature. The solvent was concentrated under reduced pressure. The resulting residue was purified by direct crystallization to afford *N*-prop-2-ynylphthalimide (A) as a colourless solid. Subsequently, the reaction of *N*-prop-2-ynylphthalimide (1 mmol) with TMSN_3_ (1.5 mmol) in DMF : H_2_O (9 : 1) (3 mL) was carried out in the presence of CuI (10 mol%) at 100 °C. Reaction progress was monitored using TLC and, after the reaction had finished, the reaction mixture was extracted with ethyl acetate. The organic phase was dried over Na_2_SO_4_ and the solvent was evaporated under vacuum. The crude residue was purified by silica-gel column chromatography to give the desired product. Finally, to a solution of the product (3.7 mmol) in EtOH (10 mL) was added hydrazine hydrate (3.7 mmol) and stirred under reflux for 3 h. After cooling and filtration, the filtrate was acidified with HCl, and filtered once more. The volatile solvent of the filtrate was removed and (triazole-5-yl) methanamine was crystallized as salts from ethyl acetate.

### Synthesis of Cu@TZMA@MCM-41

Preparation of Cu@TZMA@MCM-41 was carried out in four steps. In the first step, according to the literature, MCM-41 was synthesized using a sol–gel protocol.^33^ Briefly, in a round-bottomed flask (500 mL), 1.75 mL of NaOH (2 M) solution was added to 240 mL of deionized water with stirring and heating at 80 °C. Next, 0.5 g (1.37 mmol) of the surfactant cetyltrimethylammonium bromide (CTAB) was added. After homogenization of the solution, 2.5 mL of tetraethyl orthosilicate (TEOS) was dropped slowly into the solution, to prepare a white slurry. The obtained mixture was refluxed for 2 h under continuous stirring. After cooling to room temperature, the resulting mixture was separated by filtration, washed with deionized water, and calcined at 600 °C for 5 h at a rate of 2 °C min^−1^. In the second step, silanization of the synthesized MCM-41 (2.4 g) was carried out by refluxing of 3-chloropropyl trimethoxysilane (CPTMS) (2.5 g) in *n*-hexane (48 mL) under a N_2_ atmosphere and stirring for 24 h. The obtained solid Cl-(CH_2_)_3_@MCM-41 was washed thrice with *n*-hexane and dried under vacuum. In the third step, in a 50 mL round-bottomed flask, under continuous stirring, Cl-(CH_2_)_3_@MCM-41 (0.5 g), (triazole-5-yl)methanamine (1 mmol) and Et_3_N (1 mL) were added to DMF (20 mL) at 100 °C for 30 h to afford (triazole-5-yl)methanamine@MCM-41. Then, the prepared TZMA@MCM-41 was filtered and washed several times by ethanol and dried in a vacuum oven. Finally, a mixture of the resulting TZMA@MCM-41 (1.5 g) and CuCl_2_ anhydrous (0.5 g) was dispersed in EtOH (25 mL) under ultrasonic agitation for 20 min and stirred under reflux for 20 h. Then, the nano-catalyst (Cu@TZMA@MCM-41) was separated and washed thoroughly using ethanol and dried under a vacuum.

### General procedure for preparation of pyridine-containing pseudopeptides in the presence of Cu@TZMA@MCM-41 (7a–n)

Primary amine (1 mmol), aldehyde (1 mmol) and 2 mL of MeOH were added to a 25 mL round-bottomed flask equipped with a magnetic stirrer at room temperature. After 15 min, propiolic acid (1 mmol) was added to the reaction flask and stirring was continued for 5 min. Isocyanide (1 mmol) was added and the mixture stirred at ambient temperature for 1 day. Reaction progress was monitored using TLC. Upon reaction completion, without isolation or purification, propargylamine was added to the Ugi mixture. The reaction mixture was stirred at 60 °C for 8 h. After reaction completion, the solvent was removed using a rotary evaporator. Without purification, DMSO (2 mL) and Cu@TZMA@MCM-41 (7 mg, 0.43 mmol%) were added to the flask and the reaction mixture stirred at room temperature. Reaction progress was monitored using routine TLC. Upon reaction completion, the nano-catalyst was separated by filtration and recycled as such for the next experiment. The mixture was diluted with water and EtOAc. The organic layer was separated and washed with brine, dried over anhydrous MgSO_4_, and evaporated under reduced pressure. The obtained residue was purified using column chromatography on silica gel to obtain the pure product. The yield was 75–92%.

## Conclusions

In conclusion, we have successfully established an efficient route toward the synthesis of a diverse array of pyridine-containing pseudopeptide backbone derivatives in the presence of a novel nano-catalyst through the design of an expedient post-transformation reaction sequence. This approach was efficient, facile, cost-effective, highly diverse, and high-yielding. We obtained products from readily available starting materials. Also, a new copper complex supported on MCM-41 was synthesized and fully characterized using different methods. The design of novel cyclization reactions using 1,5-enynes in the presence of novel catalysts is in progress in our laboratory.

## Conflicts of interest

There are no conflicts to declare.

## Supplementary Material

RA-010-C9RA10885H-s001
