# Supplementary material for: Copper (triazole-5-yl)methanamine complexes onto MCM-41: the synthesis of pyridine-containing pseudopeptides through the 6-endo-dig cyclization of 1,5-enynes
Source: RSC Adv. 2020 Mar 12;10(18):10577–83. doi: 10.1039/c9ra10885h (PMC9050386; doi:10.1039/c9ra10885h)
Supplement: RA-010-C9RA10885H-s001 [file RA-010-C9RA10885H-s001.pdf]

Electronic Supplementary Material (ESI) for RSC Advances.
This journal is © The Royal Society of Chemistry 2020

Supporting Information

Copper (triazole-5-yl)methanamine complexes onto MCM-41: synthesis of pyridine containing pseudopeptides through 6-*endo*-dig cyclization of 1,5-enynes

Neda Akbarikalani,[a] Kmaran Amiri,[a] Ahmed Al-Harrasi, [b*] Saeed Balalaie [a,c*]

[a] Peptide Chemistry Research Center, K. N. Toosi University of Technology, P. O. Box 15875-4416, Tehran, Iran. E-mail: balalaie@kntu.ac.ir; Fax: +98-21-22889403; Tel: +98-21-23064226

[b] Chair of Oman's Medicinal Plants and Marine Natural Products, University of Nizwa, P.O. Box 33, Postal Code 616, Birkat Al Mauz, Nizwa, Sultanate of Oman

[c] Medical Biology Research Center, Kermanshah University of Medical Sciences, Kermanshah, Iran

Content:

Spectral data for the synthesized compounds 2-6

^{1}H, ^{13}C NMR, FT-IR and HRMS spectra 7-43

Spectral data:

***N*-(1-(4-Bromophenyl)-2-(cyclohexylamino)-2-oxoethyl)-*N*-phenyl-3-(prop-2-yn-1-ylamino) acrylamide (6b)**.

Colorless solid (234 mg, 96%), m.p. 143-146 ºC. **^{1}H NMR** ($CDCl_3$, 300 MHz) δ (ppm) (mixture of two diastereomers (*Z*:*E*,74:26)) = 1.03−1.20 (m, 3H, H-cyc (mixture of two diastereomers)), 1.23−1.42 (m, 2H, H-cyc (mixture of two diastereomers)), 1.55−1.71 (m, 3H, H-cyc (mixture of two diastereomers)), 1.80−1.97 (m, 2H, H-cyc (mixture of two diastereomers)), 2.14 (t, 1H, *J* = 2.4 Hz, acetylenic H (minor)), 2.27 (t, 1H, *J* = 2.4 Hz, acetylenic H (major)), 3.52−3.55 (m, 1H, H-cyc (minor)), 3.70−3.90 (m, 3H, CH_2-NH, H-cyc (major)), 4.17 (d, 1H, *J* = 8.3 Hz, H_a-vinyl (major)), 4.51 (d, 1H, *J* = 12.9 Hz, H_a-vinyl (minor)), 4.69− 4.75 (m, 1H, NH (minor)), 6.04 (s, 1H, −C(sp^3)−H (major)), 6.05 (s, 1, −C(sp^3)−H (minor)), 6.40− 6.56 (m, 2H, amidic NH, NH (major)), 7.00−7.09 (m, 4H, H−Ar (mixture of two diastereomers)), 7.20−7.23 (m, 3H, H−Ar (mixture of two diastereomers)), 7.30 (d, 2H, *J* = 6.7 Hz, H−Ar (mixture of two diastereomers)), 7.45 (dd, 1H, *J* = 12.6,7.8 Hz, H_b-Vinyl (minor)), 8.39− 8.47 (m, 1H_b, H-Vinyl (major)). **^{13}C NMR** ($CDCl_3$, 75 MHz) δ (ppm) (mixture of two diastereomers) = 24.7, 24.8, 25.5, 32.8, 36.9, 40.9, 48.3, 64.1, 72.7, 79.4, 122.0, 127.7, 128.9, 130.5, 131.2, 131.6, 134.7, 140.8, 149.3, 169.3, 171.0. **IR** *v* (cm^{-1}): 3298, 2114, 1672.

***N*-(2-(Cyclohexylamino)-2-oxo-1-phenylethyl)-*N*-(4-nitrophenyl)-3-(prop-2-yn-1-ylamino)acrylamide (6h).**

Colorless solid (216 mg, 94%), m.p. 134-137 ºC. **^{1}H NMR** ($CDCl_3$, 300 MHz) δ (ppm) (mixture of two diastereomers (*Z*:*E*,78:22))= 0.93−1.99 (m, 10H, H-cyc (mixture of two diastereomers)), 2.15 (t, 1H, *J* = 2.4 Hz, acetylenic H (minor)), 2.28 (t, 1H, *J* = 2.4 Hz, acetylenic H (major)), 3.56−3.58 (m, 1H, H-cyc (minor)), 3.74−3.89 (m, 3H, -CH_2-NH, H-cyc (major)), 4.07 (d, 1H, *J* = 8.2 Hz, H_a-vinyl (major)), 4.43 (d, 1H, *J* = 12.8Hz, H_a-vinyl (major)), 4.75− 4.80 (m, 1H, NH (minor)), 5.91 (d, 1H, *J* = 8.0 Hz, amidic NH (minor)), 5.98 (d, 1H, *J* = 8.0 Hz, amidic NH (major)), 6.27 (s, 1H, −C(sp^3)−H (major)), 6.29 (s, 1H, −C(sp^3)−H (minor)), 6.51 (dd, 1H, *J*= 8.2,4.2 Hz, NH (major)), 7.13− 7.22 (m, 5H, H−Ar (mixture of two diastereomers)), 7.33 (d, 2H, *J*= 8.1 Hz, H−Ar (mixture of two diastereomers)), 7.99 (d, 2H, *J* = 9.0 Hz, H−Ar (mixture of two diastereomers)), 7.50 (dd, 1H, *J*= 13.5,8.2 Hz, H_b-Vinyl (minor)), 8.45− 8.53 (m, 1H, H_b-Vinyl (major)). **^{13}C NMR** ($CDCl_3$, 75 MHz) δ (ppm) (mixture of two diastereomers)= 24.7, 24.9, 25.5, 32.8, 32.9, 36.9, 48.7, 63.7(major), 64.3(minor), 65.8, 72.5, 72.9, 78.4, 79.2, 85.2, 89.3,123.6, 123.7, 128.5, 128.6, 130.0, 132.0, 132.1, 134.9, 135.0, 146.3, 149.4, 146.9, 147.1, 147.4, 149.8, 168.2(minor), 169.2(minor), 169.3(major), 170.1(major). **IR** *v* (cm^{-1}): 3293, 2241, 1671.

***N*-(2-(*tert*-Butylamino)-1-(4-chlorophenyl)-2-oxoethyl)-*N*-phenyl-3-(prop-2-yn-1-ylamino)acrylamide (6n).**

Colorless solid (192 mg, 91%), m.p. 140-142 ºC. **^{1}H NMR** ($CDCl_3$, 300 MHz) δ (ppm) (mixture of two diastereomers (85:15))= 1.35 (brs, 18H, H-cyc (mixture of two diastereomers)), 2.14 (t, 1H, *J* = 2.4 Hz, acetylenic H (minor)), 2.28 (t, 1H, *J* = 2.4 Hz, acetylenic H (major)), 3.51−3.55 (m, 1H, -CH_2N (minor)), 3.80−3.95 (m, 1H, -CH_2N (major)), 4.17 (d, 1H, *J* = 8.3 Hz, H_a-Vinyl (major)), 4.50 (d, 1H, *J* = 12.9 Hz, H_a-Vinyl (minor)), 4.70− 4.78 (m, 1H, H−NH (minor)), 5.99

(s, 1H, −C(sp^3)−H (major)), 6.07 (s, 1H, −C(sp^3)−H (minor)), 6.40 (brs, 1H, amidic NH (mixture of two diastereomers)), 6.45 (dd, 1H, J= 12.3,8.4, NH (major)), 6.98− 7.27 (m, 18H, H−Ar (mixture of two diastereomers)), 8.44− 8.52 (m, 1H, H_b-Vinyl (mixture of two diastereomers)). ^{13}**C NMR** ($CDCl_3$, 75 MHz) δ (ppm) (mixture of two diastereomers)= 28.7, 36.9, 51.3, 64.4, 72.4, 72.7, 76.6, 78.5, 79.4, 85.9, 127.7, 128.1, 128.2, 128.8, 128.9, 130.4, 130.5, 131.2, 131.5, 133.7, 134.2, 140.8, 149.3, 169.4, 170.8. **IR** *ν* (cm^{-1}): 3289, 2110, 1682.

***N*-(2-(Cyclohexylamino)-2-oxo-1-phenylethyl)-*N* phenylnicotinamie (7a).**

Colorless solid (191 mg, 92%), m.p. 153-155 ºC. ^{1}**H NMR** ($CDCl_3$, 300 MHz) δ (ppm) = 1.02−1.18 (m, 3H, H-cyc), 1.32−1.36 (m, 2H, H-cyc), 1.55−1.70 (m, 3H, H-cyc), 1.87−1.98(m, 2H, H-cyc), 3.86−3.88 (m, 1H, H-cyc), 5.75 (d, 1H, *J* = 7.6 Hz, NH), 6.19 (s, 1H −C(sp^3)−H), 7.01−7.06 (m, 6H, H−Ar), 7.22-7.27 (m, 5H, H−Ar, H−Py), 7.59 (d, 1H, *J* = 7.8 Hz, H−Py), 8.37 (d, 1H, *J* = 3.8 Hz, H−Py), 8.51(s, 1H, H−Py). ^{13}**C NMR** ($CDCl_3$, 75 MHz) δ (ppm) = 24.7, 24.8, 25.4, 32.7, 48.9, 66.4, 122.5, 127.6, 128.5, 128.6, 130.3, 130.5, 132.1, 134.4, 135.8, 140.3, 149.3, 150.0, 168.2, 168.7. **HRMS** (ESI): Calc. for $C_{26}H_{28}N_3O_2$ $[M+H]^+$ 414.2208, Found 414.2214; **IR** *ν* (cm^{-1}): 3273, 1645.

***N*-(1-(4-Bromophenyl)-2-(cyclohexylamino)-2-oxoethyl)-*N*-phenyl-nicotinamide (7b).**

Colorless solid (222 mg, 90%), m.p. 218-221 ºC. ^{1}**H NMR** ($CDCl_3$, 300 MHz) δ (ppm) = 0.99−1.24 (m, 3H, H-cyc), 1.32−1.34 (m, 2H, H-cyc), 1.56−1.66 (m, 3H, H-cyc), 1.86−1.97 (m, 2H, H-cyc), 3.83−3.86 (m, 1H, H-cyc), 5.84 (d, 1H, *J* = 7.5 Hz, NH), 6.13 (s, 1H −C(sp^3)−H), 7.05−7.12 (m, 8H, H−Ar, H−Py), 7.35 (d, 2H, *J* = 8.0 Hz, H−Ar), 7.58 (d, 1H, *J* = 7.7 Hz, H−Py), 8.39 (d, 1H, *J* = 3.8 Hz, H−Py), 8.50 (s, 1H, H−Py). ^{13}**C NMR** ($CDCl_3$, 75 MHz) δ (ppm) = 24.7, 24.8, 25.4, 32.8, 48.9, 65.6, 122.5, 122.9, 127.9, 128.8, 130.5, 131.7, 131.8, 131.9,133.4, 135.8, 140.1, 149.2, 150.2, 167.8, 168.8. **HRMS** (ESI): Calc. for $C_{26}H_{27}BrN_3O_2$ $[M+H]^+$ 492.1062, Found 492.1069. **IR** *ν* (cm^{-1}): 3273, 1645.

***N*-(1-(4-Chlorophenyl)-2-(cyclohexylamino)-2-oxoethyl)-*N*-phenyln icotinamide (7c).**
Colorless solid (210 mg, 89%), m.p. 216-219 ºC. ^{1}**H NMR** ($CDCl_3$, 300 MHz) δ (ppm) = 1.05−1.16 (m, 3H, H-cyc), 1.30−1.37 (m, 2H, H-cyc), 1.55−1.65 (m, 3H, H-cyc), 1.84−1.97(m, 2H, H-cyc), 3.82−3.86 (m, 1H, H-cyc), 5.91 (d, 1H, *J* = 8.0 Hz, NH), 6.16 (s, 1H −C(sp^3)−H), 6.95−7.06 (m, 6H, H−Ar), 7.13−7.20 (m, 4H, H−Ar, H−Py), 7.56 (dt, 1H, *J* = 7.9,1.8 Hz, H−Py), 8.37 (d, 1H, *J* = 3.8 Hz, H−Py), 8.49 (s, 1H, H−Py). ^{13}**C NMR** ($CDCl_3$, 75 MHz) δ (ppm) = 24.7, 24.8, 25.4, 32.7, 48.9, 65.4, 122.5, 127.9, 128.7, 128.8, 130.5, 131.6, 131.9, 132.9, 134.7, 135.8, 140.0, 149.2, 150.2, 167.9, 168.8. **HRMS** (ESI): Calc. for $C_{26}H_{26}ClN_3O_2Na$ $[M+Na]^+$ 470.1616, Found 470.1609. **IR** *ν* (cm^{-1}): 3267, 1643.

***N*-(2-(Cyclohexylamino)-2-oxo-1-(*p*-tolyl)ethyl)-*N*-phenylnicotinam ide (7d).**

Colorless solid (200 mg, 90%), m.p. 171-173 ºC. ^{1}**H NMR** ($CDCl_3$, 300 MHz) δ (ppm) = 1.06−1.15 (m, 3H, H-cyc), 1.31−1.37 (m, 2H, H-cyc), 1.59−1.66 (m, 3H, H-cyc), 1.90−2.05 (m, 2H, H-cyc), 3.74 (s, 3H, −Me), 3.84−3.87 (m, 1H, H-cyc), 5.69 (d, 1H, *J* = 8.0 Hz, NH), 6.15 (s, 1H −C(sp3)−H), 6.74 (d, 2H, *J* = 8.7 Hz, H−Ar), 7.01− 7.06 (m, 6H, H−Ar, H−Py), 7.12 (d, 2H, *J* = 8.7 Hz, H−Ar), 7.58 (dt, 1H, *J* = 7.9,1.8 Hz, H−Py) 8.36 (d, 1H, *J* = 4.2, Hz, H−Py), 8.50(s,

1H, H−Py). **^{13}C NMR** ($CDCl_3$, 75 MHz) δ (ppm) = 24.7, 24.8, 25.4, 32.8, 48.8, 55.1, 65.6, 113.8. 122.4, 126.3, 127.6, 128.6, 130.7, 131.7, 132.2, 135.8, 140.3, 149.2, 149.3, 150.0, 159.7, 168.4, 168.6. **HRMS** (ESI): Calc. for $C_{27}H_{30}N_3O_3$ $[M+H]^+$ 444.2320, Found 444.2326; **IR** *v* (cm^{-1}): 3277, 1649.

***N*-(2-(Cyclohexylamino)-1-(4-methoxyphenyl)-2-oxoethyl)-*N*-phenylnicotinamide (7e).**

Colorless solid (172 mg, 80%), m.p. 205-207 ºC. **^{1}H NMR** ($CDCl_3$, 300 MHz) δ (ppm) = 1.05−1.15 (m, 3H, H-cyc), 1.32−1.34 (m, 2H, H-cyc), 1.60−1.66 (m, 3H, H-cyc), 1.91−1.92(m, 2H, H-cyc), 2.28 (s, 3H, −Me), 3.84−3.88 (m, 1H, H-cyc), 5.69 (d, 1H, *J* = 7.0 Hz, NH), 6.14 (s, 1H −C(sp^3)−H), 7.02−7.06 (m, 8H, H−Ar), 7.09− 7.12 (m, 2H, H−Ar, H−Py), 7.57−7.61 (dt, 1H, *J* = 7.8,1.8 HZ, H−Py), 8.37 (dd, 1H, *J* = 8.3,1.5 Hz, H−Py), 8.51(d, 1H, *J* = 1.5 Hz, H−Py). **^{13}C NMR** ($CDCl_3$, 75 MHz) δ (ppm) = 21.1, 24.7, 24.8, 25.4, 32.8, 48.8, 66.2, 122.4, 127.6, 128.6, 129.2, 130.2, 132.2, 135.8, 138.5, 140.5, 149.5, 149.3, 150.0, 168.3, 168.6. **HRMS** (ESI): Calc. for $C_{27}H_{30}N_3O_2$ $[M+H]^+$ 428.2360, Found 428.2365; **IR** *v* (cm^{-1}): 3267, 1645.

***N*-(2-(Cyclohexylamino)-1-(3-nitrophenyl)-2-oxoethyl)-*N*-phenylnic otinamide (7f).**

Colorless solid (189 mg, 82%), m.p. 146-149 ºC. **^{1}H NMR** ($CDCl_3$, 300 MHz) δ (ppm) = 1.08−1.25 (m, 3H, H-cyc), 1.33−1.36 (m, 2H, H-cyc), 1.57−1.68 (m, 3H, H-cyc), 1.88−1.97(m, 2H, H-cyc), 3.86−3.89 (m, 1H, H-cyc), 6.25 (d, 1H, *J* = 7.2 Hz, NH), 6.34 (s, 1H −C(sp^3)−H), 7.05−7.06 (m, 6H, H−Ar), 7.38 (t, 1H, *J* = 7.8, H−Ar), 7.59 (d, 5H, *J* = 6.8 Hz, H−Ar), 8.08 (d, 1H, *J* = 7.8 Hz, H−Py) 8.15(s, 1H, H−Py), 8.41 (d, 1H, *J* = 3.5 Hz, H−Py), 8.51(s, 1H, H−Py). **^{13}C NMR** ($CDCl_3$, 75 MHz) δ (ppm) = 24.6, 24.7, 25.3, 32.7, 49.0, 65.1, 122.6, 123.4, 125.2, 128.2, 129.0, 129.3, 130.3, 131.5, 135.9, 136.1, 136.4, 139.6, 147.9, 149.3, 150.4, 167.3, 168.9. **HRMS** (ESI): Calc. for $C_{26}H_{27}N_4O_4$ $[M+H]^+$ 459.2115, Found 459.2123; **IR** *v* (cm^{-1}): 3281, 1649.

***N*-(2-(Cyclohexylamino)-2-oxo-1-phenylethyl)-*N*-(2-iodophenyl)nic otinamide (7g).**

Colorless solid (202 mg, 75%), m.p. 198-201 ºC. **^{1}H NMR** ($CDCl_3$, 300 MHz) (mixture of two rotamers (57:43)) δ (ppm) = 1.03−1.18 (m, 7H, H-cyc (mixture of two rotamers)), 1.24−1.34 (m, 4H, H-cyc (mixture of two rotamers)), 1.54−1.64 (m, 6H, H-cyc(mixture of two rotamers)), 1.83−1.84 (m, 2H, H-cyc (mixture of two rotamers)), 1.91−1.99 (m, 1H, H-cyc(mixture of two rotamers)), 3.82−3.85 (m, 2H, H-cyc (mixture of two rotamers)), 5.94 (d, 1H, *J* = 7.8 Hz, NH (minor rotamer)), 6.09 (d, 1H, *J* = 7.6 Hz, NH (major rotamer)), 6.23 (s, 1H −C(sp^3)−H (minor rotamer)), 6.28 (s, 1H −C(sp^3)−H (major rotamer)), 6.69−6.72 (m, 2H, H−Ar (mixture of two rotamers)), 7.04−7.12 (m, 7H, H−Ar), 7.20−7.38 (m, 9H, H−Ar, H−Py (mixture of two rotamers)), 7.70−7.73 (m, 2H, H−Ar (mixture of two rotamers)), 8.02−8.05 (m, 2H, H−Py(mixture of two rotamers)), 8.38 (s , 2H, H−Py (mixture of two rotamers)), 8.64 (d, 2H, *J* =7.4 Hz, H−Py (mixture of two rotamers)). **^{13}C NMR** ($CDCl_3$, 75 MHz) (mixture of two rotamers) δ (ppm) =18.8,, 24.7, 24.8, 25.4, 29.6, 32.5, 32.6, 32.7, 49.1, 64.7, 65.7, 68.2, 103.6, 122.1, 128.0, 128.2, 128.5, 128.6, 128.7, 129.6, 129.9, 130.9, 131.2, 132.3, 132.6, 133.5,134.8, 136.1, 139.3, 141.1, 141.3, 149.4, 150.1, 168.3, 168.5. 168.7, 168.8. **HRMS** (ESI): Calc. for $C_{26}H_{26}IN_3O_2$ $[M+H]^+$ 540.1170, Found 540.1174. **IR** *v* (cm^{-1}): 3264, 1653.

***N*-(2-(Cyclohexylamino)-2-oxo-1-phenylethyl)-*N*-(4-nitrophenyl)nic otinamide (7h).** Colorless solid (211 mg, 92%), m.p. 169-172 °C. **^{1}H NMR** ($CDCl_3$, 300 MHz) δ (ppm) = 0.96−1.20 (m, 3H, H-cyc), 1.24−1.39 (m, 2H, H-cyc), 1.56−1.66 (m, 3H, H-cyc), 1.92−1.93(m, 2H, H-cyc), 3.80−3.90 (m, 1H, H-cyc), 5.80 (d, 1H, *J* = 7.8 Hz, NH), 6.35 (s, 1H −C(sp^3)−H), 7.08−7.24 (m, 8H, H−Ar, H−Py), 7.60 (dt, 1H, *J* =6.1,1.6 Hz, H−Py), 7.82 (d, 2H, *J* = 9.0 Hz, H−Ar), 8.41 (d, 1H, *J* = 4.1 Hz, H−Py), 8.48(s, 1H, H−Py). **^{13}C NMR** ($CDCl_3$, 75 MHz) δ (ppm) = 24.6, 24.8, 25.3, 32.7, 42.6, 49.1, 65.4, 122.8, 123.6, 128.9, 129.1, 130.2, 131.4, 131.6, 133.6, 135.8, 146.1, 146.2, 149.2, 150.7, 168.0, 168.4. **HRMS** (ESI): Calc. for $C_{26}H_{27}N_4O_4$ $[M+H]^+$ 459.2054, Found 459.2128; Calc. for $C_{52}H_{53}N_8O_8$ $[2M+H]^+$ 917.4140, Found 917.4147. **IR** *v* (cm^{-1}): 3277, 1651.

***N*-(2-(Cyclohexylamino)-2-oxo-1-phenylethyl)-*N*-(4-nitrophenyl)ni cotinamide (7i).**

Colorless solid (190 mg, 86%), m.p. 137-138 °C. **^{1}H NMR** ($CDCl_3$, 300 MHz) δ (ppm) = 1.06−1.15 (m, 3H, H-cyc), 1.35−1.36 (m, 2H, H-cyc), 1.59−1.64 (m, 3H, H-cyc), 1.91−1.99 (m, 2H, H-cyc), 2.02 (s, 3H, −Me), 2.05 (s, 3H, −Me), 3.85−3.87 (m, 1H, H-cyc), 5.79 (d, 1H, *J* = 8.0 Hz, NH), 6.05 (s, 1H −C(sp^3)−H), 6.65−6.67 (m, 1H, H−Ar), 6.74−6.79 (m, 2H, H−Ar), 7.04−7.08 (m, 1H, H−Ar), 7.25−7.27 (m, 5H, H−Ar, H−Py), 7.64 (dt, 2H, *J* =7.9,1.8 Hz, H−Py), 8.37 (d, 1H, *J* = 4.7 Hz, H−Py), 8.49(s, H, H−Py). **^{13}C NMR** ($CDCl_3$, 75 MHz) δ (ppm) =19.2,19.5, 24.7, 24.8, 25.4, 32.7, 48.7, 67.2, 122.5, 127.5, 128.4, 129.7, 130.1, 130.9, 132.3, 134.7, 136.0, 136.1, 137.1, 138.3, 149.2, 149.3, 149.9, 168.2, 168.7. **HRMS** (ESI): Calc. for $C_{28}H_{32}N_3O_2$ $[M+H]^+$ 442.2525, Found 442.2532; **IR** *v* (cm^{-1}): 3287, 1647.

***N*-Benzyl-*N*-(2-(cyclohexylamino)-2-oxo-1-phenylethyl) amidnicotine (7j).**

Colorless solid (175 mg, 82%), m.p. 80-83 °C. **^{1}H NMR** ($CDCl_3$, 300 MHz) δ (ppm) = 1.02−1.11 (m, 3H, H-cyc), 1.26−1.35 (m, 2H, H-cyc), 1.55−1.66 (m, 3H, H-cyc), 1.84−1.89 (m, 2H, H-cyc), 3.81−3.89 (m, 1H, H-cyc), 4.47 (d, 1H, *J* = 16.7 Hz, -CH_2 Ph), 4.71 (d, 1H, *J* = 16.7 Hz, -CH_2 Ph), 5.63 (s, 1H −C(sp^3)−H), 5.67 (d, 1H, *J* = 7.9 Hz, NH), 6.97− 6.99 (m, 2H, H−Ar), 7.09− 7.18 (m, 3H, H−Ar), 7.18− 7.21 (m, 1H, H−Ar), 7.31− 7.32 (m, 3H, H−Ar, H−Py), 7.36− 7.39 (m, 1H, H−Ar), 7.69 (d, 1H, *J* = 7.4 Hz, H−Py), 8.56 (d, 1H, *J* = 4.8 Hz, H−Py), 8.67 (s, 1H, H−Py). **^{13}C NMR** ($CDCl_3$, 75 MHz) δ (ppm) = 24.6, 24.7, 25.4, 32.7, 48.7, 52.2, 64.4, 123.1, 126.8, 127.1, 128.4, 128.9, 129.0, 129.7, 132.3, 134.3, 134.7, 137.1, 147.4, 150.6, 167.9, 170.7. **HRMS** (ESI): Calc. for $C_{27}H_{30}N_3O_2$ $[M+H]^+$ 428.2366, Found 428.2374; **IR** *v* (cm^{-1}): 3296, 1679.

***N*-Benzyl-*N*-(1-(4-bromophenyl)-2-(cyclohexylamino)-2-oxoethyl) nicotinamide (7k).** Colorless solid (228 mg, 90%), m.p. 93-96 °C. **^{1}H NMR** ($CDCl_3$, 300 MHz) δ (ppm) = 1.03−1.15 (m, 3H, H-cyc), 1.25−1.40 (m, 2H, H-cyc), 1.56−1.67 (m, 3H, H-cyc), 1.84−1.92 (m, 2H, H-cyc), 3.79−3.82 (m, 1H, H-cyc), 4.46 (d, 1H, *J* = 16.7 Hz, -CH_2 Ph), 4.72 (d, 1H, *J* = 16.7 Hz, -CH_2 Ph), 5.53(s, 1H −C(sp^3)−H), 5.72 (d, 1H, *J* = 7.0 Hz, NH), 6.98− 7.00 (m, 2H, H−Ar), 7.15− 7.17 (m, 3H, H−Ar), 7.21− 7.27 (m, 3H, H−Ar), 7.41− 7.44 (m, 2H, H−Ar, H−Py), 7.71 (d, 1H, *J* = 7.7 Hz, H−Py), 8.58 (d, 1H, *J* = 4.0 Hz, H−Py), 8.69 (s, 1H, H−Py). **^{13}C NMR** ($CDCl_3$, 75 MHz) δ (ppm) = 24.6, 24.7, 25.4, 32.7, 42.6, 48.8, 123.1, 123.2, 126.8, 127.3, 128.5, 131.4, 132.0, 133.8, 134.4, 136.7, 147.5, 150.8, 167.4, 170.7. **HRMS** (ESI): Calc. for $C_{27}H_{29}BrN_3O_2$ $[M+H]^+$ 506.1271, Found 506.1266. **IR** *v* (cm^{-1}): 3059, 1679.

***N*-Benzyl-*N*-(1-(4-chlorophenyl)-2-(cyclohexylamino)-2-oxoethyl) nicotinamide (7l).** Colorless solid (201 mg, 87%), m.p. 88-91 ºC. **^{1}H NMR** ($CDCl_3$, 300 MHz) δ (ppm) = 1.02−1.14 (m, 3H, H-cyc), 1.25−1.39 (m, 2H, H-cyc), 1.55−1.66 (m, 3H, H-cyc), 1.83−1.91 (m, 2H, H-cyc), 3.79−3.86 (m, 1H, H-cyc), 4.46 (d, 1H, *J* = 16.7 Hz, -CH_2 Ph), 4.72 (d, 1H, *J* = 16.7 Hz, -CH_2 Ph), 5.55 (s, 1H −C(sp^3)−H), 5.72 (d, 1H, *J* = 7.2 Hz, NH), 6.97− 6.99 (m, 2H, H−Ar), 7.15− 7.17 (m, 3H, H−Ar), 7.20− 7.31 (m, 5H, H−Ar, H−Py), 7.70 (d, 1H, *J* = 7.7 Hz, H−Py), 8.57 (d, 1H, *J* = 4.2 Hz, H−Py), 8.68 (s, 1H, H−Py). **^{13}C NMR** ($CDCl_3$, 75 MHz) δ (ppm) = 18.8, 24.6, 24.7, 25.3, 32.7, 48.7, 52.3, 68.1, 123.2, 126.8, 127.3, 128.5, 129.1, 131.1, 132.1, 133.2, 133.9, 134.3, 134.9, 136.7, 147.5, 150.8, 167.5, 170.7. **HRMS** (ESI): Calc. for $C_{27}H_{29}ClN_3O_2$ $[M+H]^+$ 462.1970, Found 462.1972; **IR** *v* (cm^{-1}): 3277, 1671.

***N*-(2-(*tert*-Butylamino)-2-oxo-1-phenylethyl)-*N*-phenylnicotinamide (7m).**

Colorless solid (141 mg, 73%), m.p. 95-96 ºC. **^{1}H NMR** ($CDCl_3$, 300 MHz) δ (ppm) = 1.36 (s, 9H, H−*t*Bu), 5.70 (s, 1H, NH), 6.11 (s, 1H −C(sp3)−H), 7.0− 7.03 (m, 6H, H−Ar), 7.22 (brs, 5H, H−Ar), 7.58 (dt, 1H, *J* = 7.9,1.8 Hz, H−Py), 8.38 (d, 1H, *J* = 2.9 Hz, H−Py), 8.52 (s, 1H, H−Py). **^{13}C NMR** ($CDCl_3$, 75 MHz) δ (ppm) = 28.6, 51.7, 66.8, 122.4, 127.6, 128.6, 130.2, 130.4, 132.2, 134.5, 135.9, 140.4, 149.3, 150.0, 168.3, 168.6. **HRMS** (ESI): Calc. for $C_{24}H_{26}N_3O_2$ $[M+H]^+$ 388.2072, Found 388.2078; **IR** *v* (cm^{-1}): 3345, 1685.

***N*-(2-(*tert*-Butylamino)-1-(4-chlorophenyl)-2-oxoethyl)-*N*-phenyl nicotinamide (7n).** Colorless solid (165 mg, 78%), m.p. 167-170 ºC. **^{1}H NMR** ($CDCl_3$, 300 MHz) δ (ppm) = 1.36 (s, 9H, H−*t*Bu), 5.76 (s, 1H, NH), 6.09 (s, 1H −C(sp3)−H), 7.04−7.15 (m, 5H, H−Ar), 7.18-7.27 (brs, 5H, H−Ar, H−Py), 7.57 (d, 1H, *J* = 7.9 Hz, H−Py), 8.39 (brs, 1H, H−Py), 8.51 (brs, 1H, H−Py). **^{13}C NMR** ($CDCl_3$, 75 MHz) δ (ppm) = 28.6, 51.8, 65.9, 122.5, 127.8, 128.7, 128.8, 130.5, 131.6, 131.9, 133.0, 134.6, 135.8, 140.0, 149.3, 150.2, 167.9, 168.6. **HRMS** (ESI): Calc. for $C_{24}H_{25}ClN_3O_2$ $[M+H]^+$ 422.1647, Found 422.1637; **IR** *v* (cm^{-1}): 3303, 1638.

2-(Prop-2-yn-1-yl)isoindoline-1,3-dione.

Colorless solid (85 mg, 92%), **^{1}H NMR** (300 MHz, Chloroform-*d*) δ 7.84 – 7.80 (m, 2H), 7.70 – 7.66 (m, 2H), 4.39 (d, *J* = 2.5 Hz, 2H), 2.16 (t, *J* = 2.5 Hz, 1H).

2-((1*H*-1,2,3-triazol-5-yl)methyl)isoindoline-1,3-dione.

Colorless solid (94 mg, 82%), **^{1}H NMR** (400 MHz, DMSO-d_6) δ 7.93 – 7.84 (m, 4H), 7.78 (s, 1H), 4.87 (s, 2H).

(1*H*-1,2,3-triazol-5-yl)methanamine hydrochloride.

Colorless solid (32 mg, 47%), **^{1}H NMR** (400 MHz, DMSO-d_6) δ 12.17 (br s, 1H), 8.75 (br s, 3H), 8.06 (s, 1H), 4.16 (q, *J* = 5.8 Hz, 2H). **^{13}C NMR** (101 MHz, DMSO-d_6) δ 137.11, 125.86, 44.61.

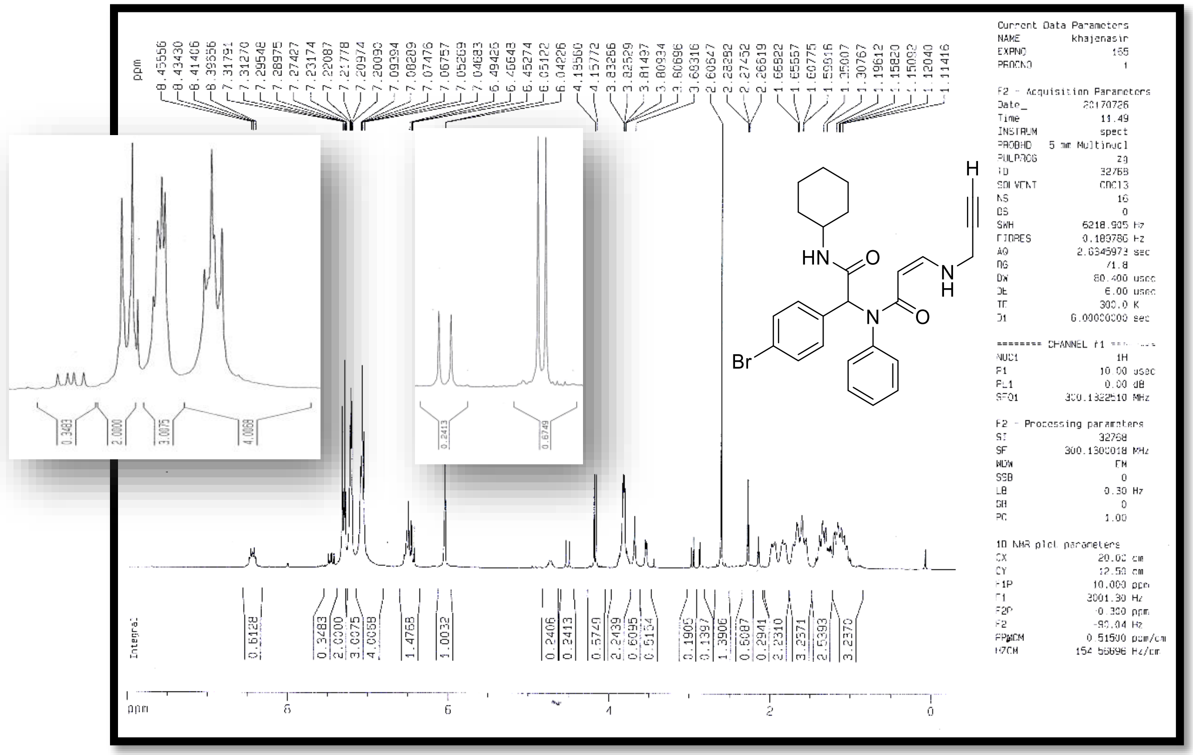

Figure **S1**: ^{1}H-NMR spectra of **6b** (300MHz, $CDCl_3$)

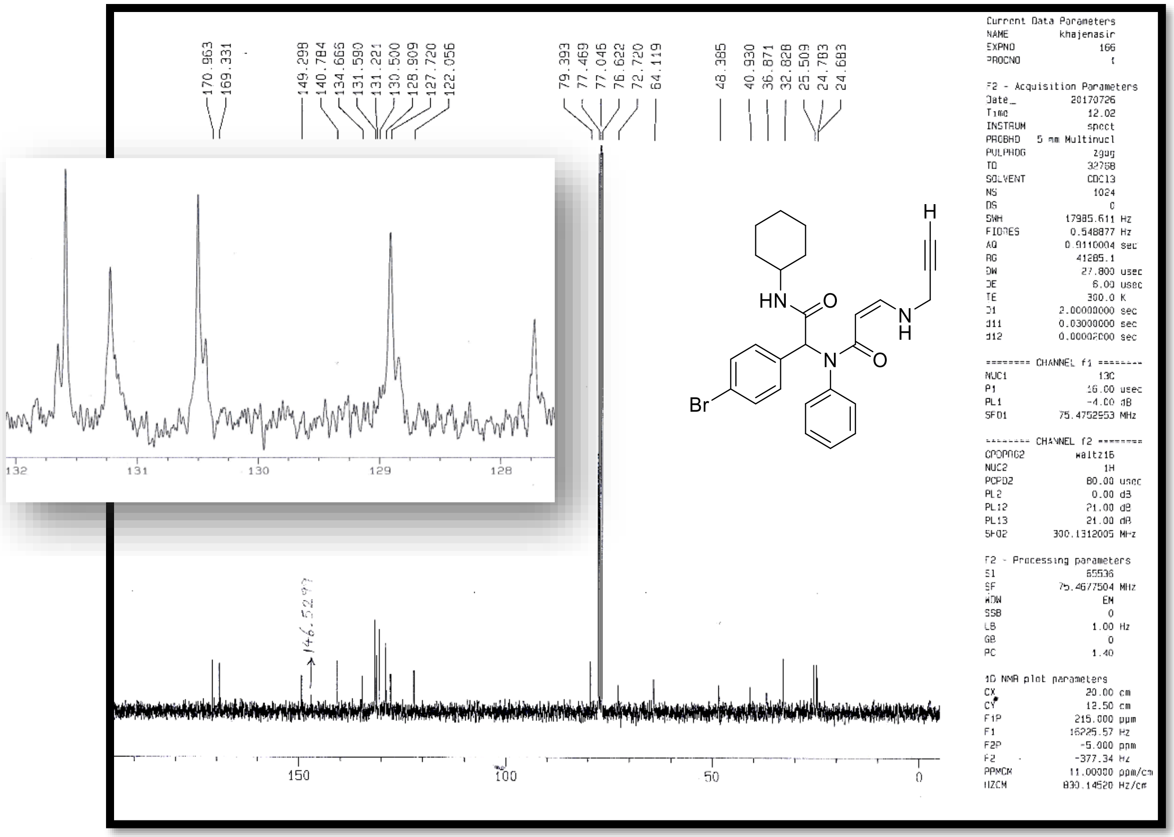

Figure **S2**: ^{13}C-NMR Spectra of **6b** (75 MHz, $CDCl_3$)

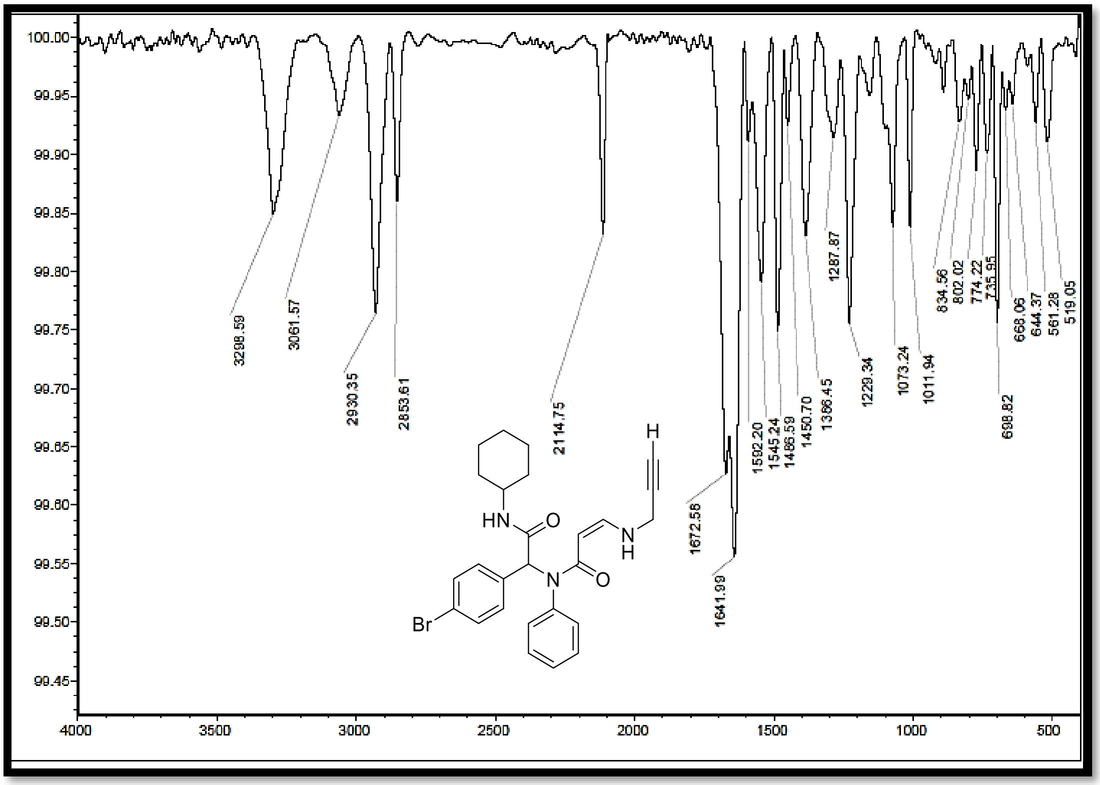

Figure **S3**: IR Spectra of **6b** (KBr, cm^{-1})

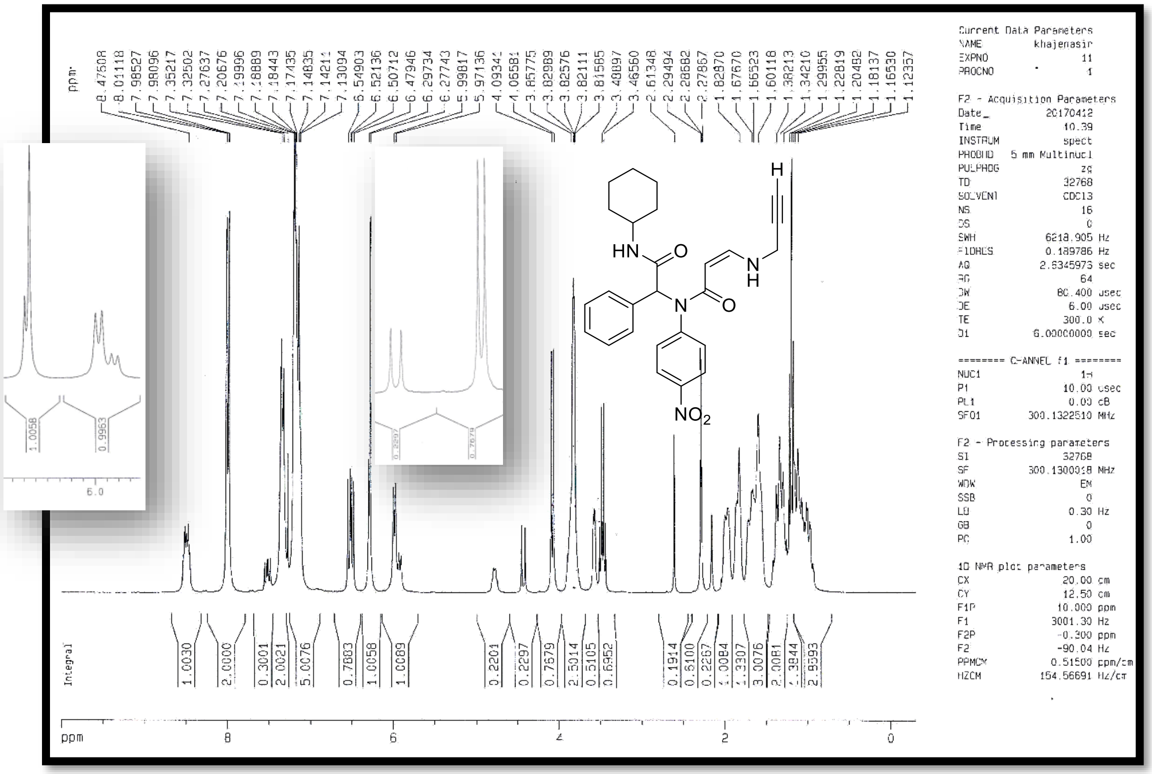

Figure **S4**: ^{1}H-NMR spectra of **6h** (300MHz, $CDCl_3$)

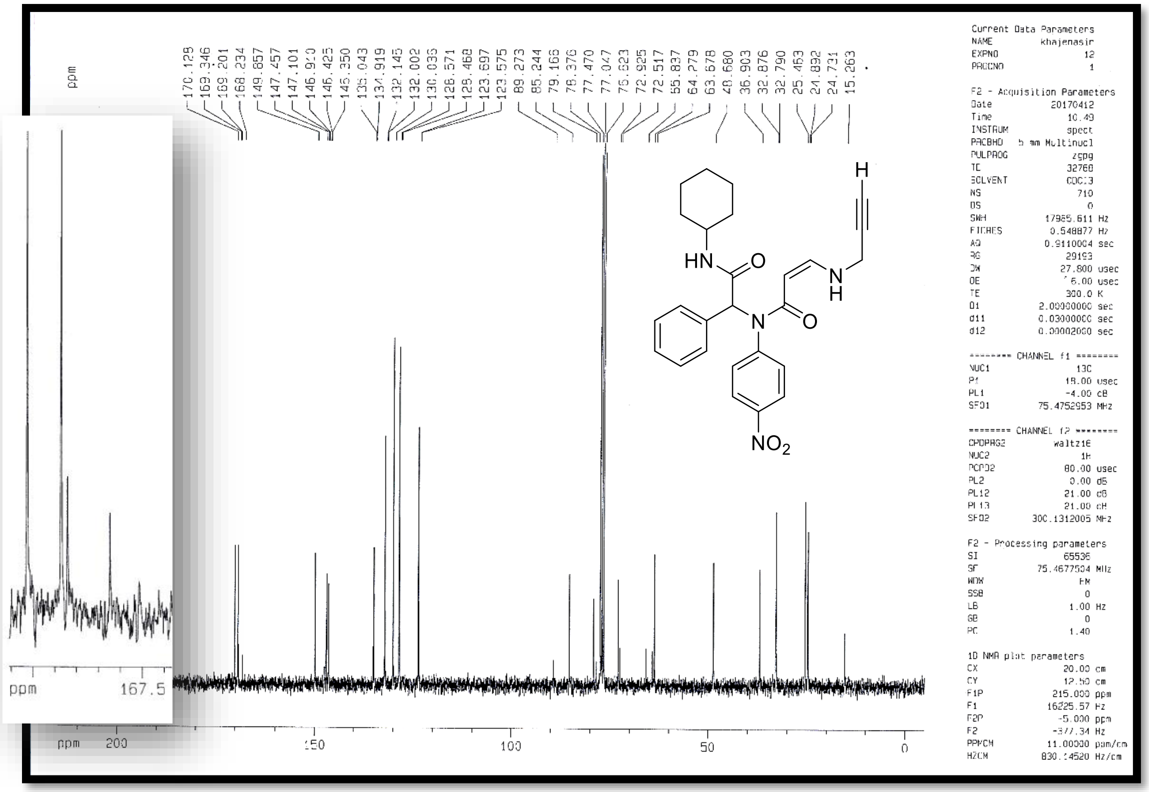

Figure **S5**: ^{13}C-NMR Spectra of **6h** (75 MHz, $CDCl_3$)

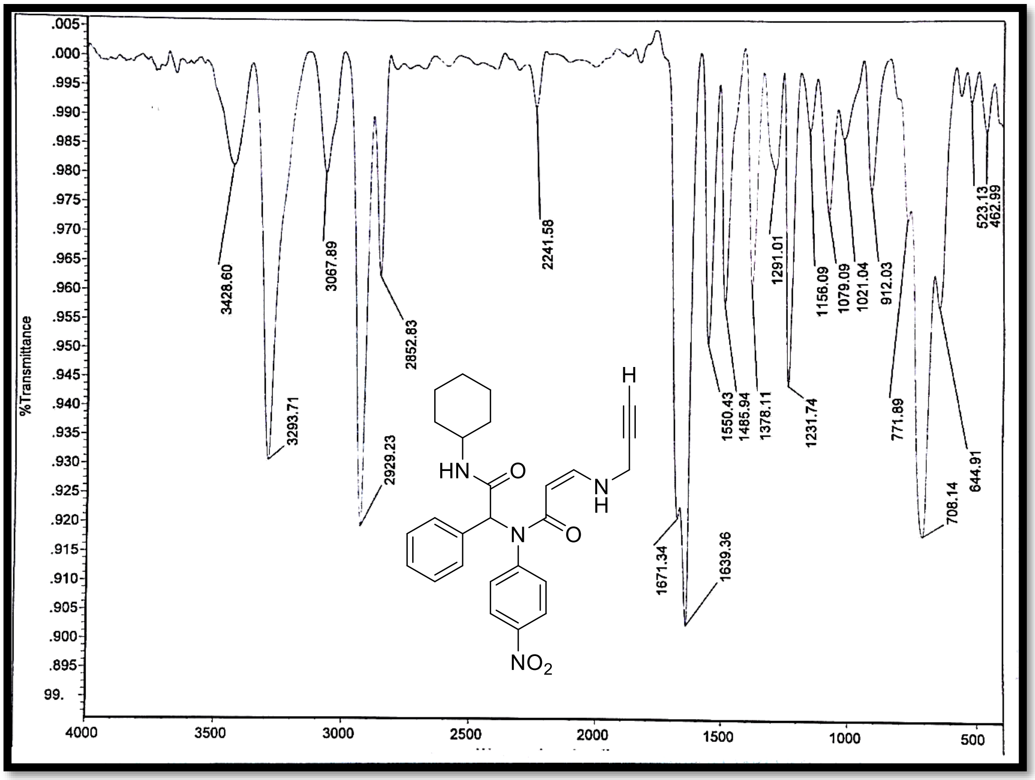

Figure **S6**: IR Spectra of **6h** (KBr, cm^{-1})

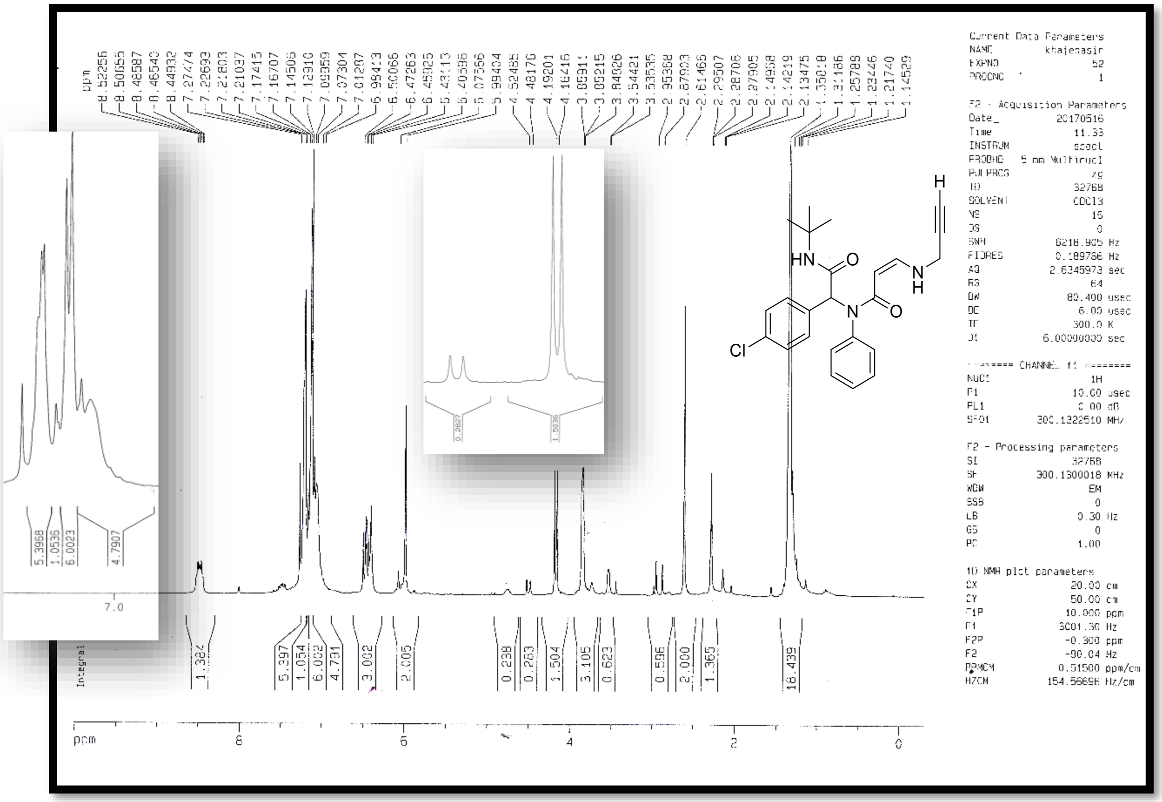

Figure **S7**: ^{1}H-NMR spectra of **6n** (300MHz, $CDCl_3$)

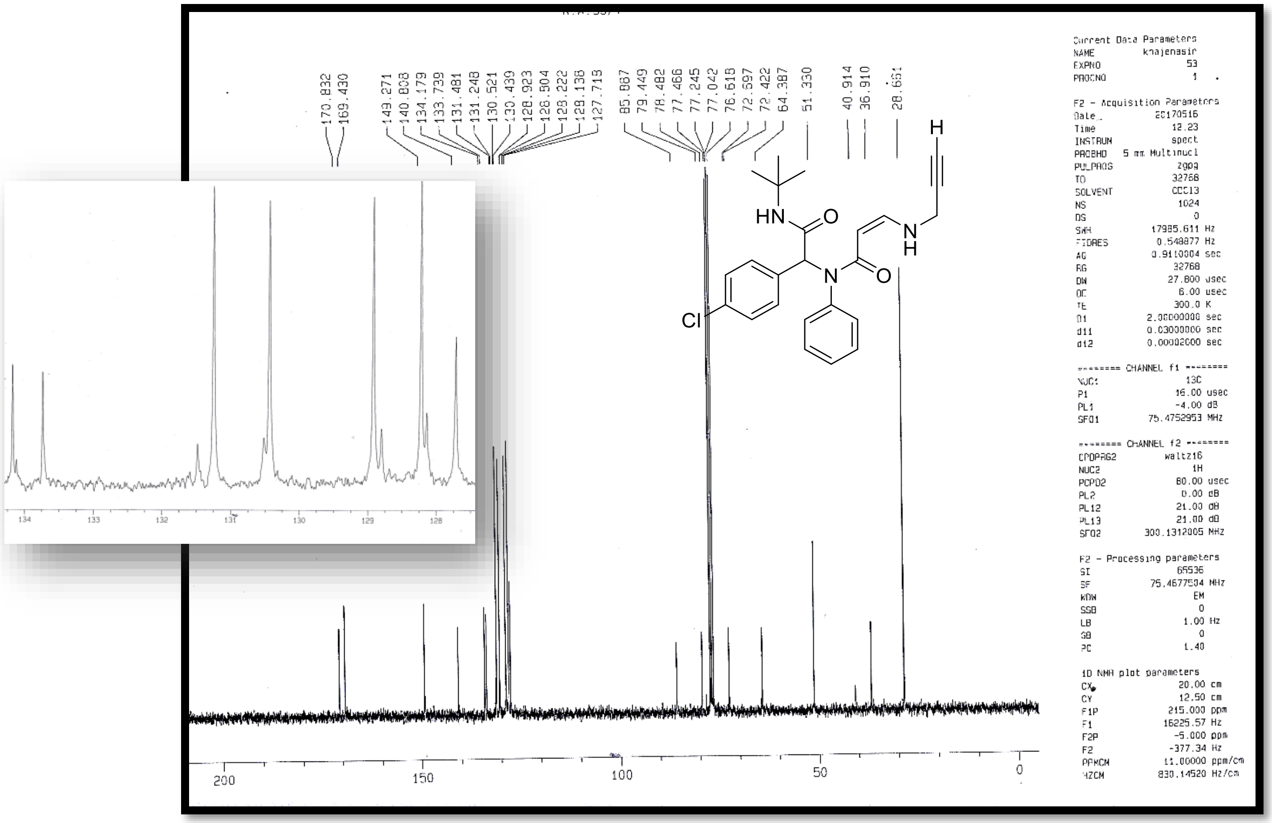

Figure **S8**: ^{13}C-NMR Spectra of **6n** (75 MHz, $CDCl_3$)

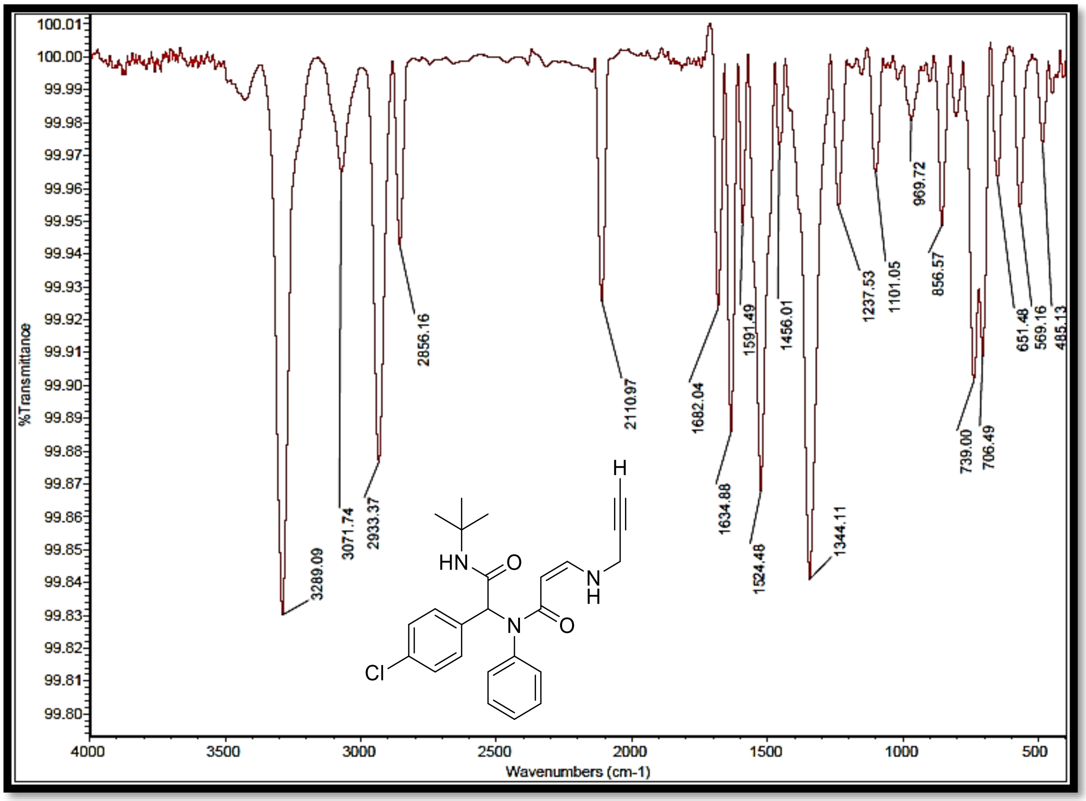

Figure **S9**: IR Spectra of **6n** (KBr, cm^{-1})

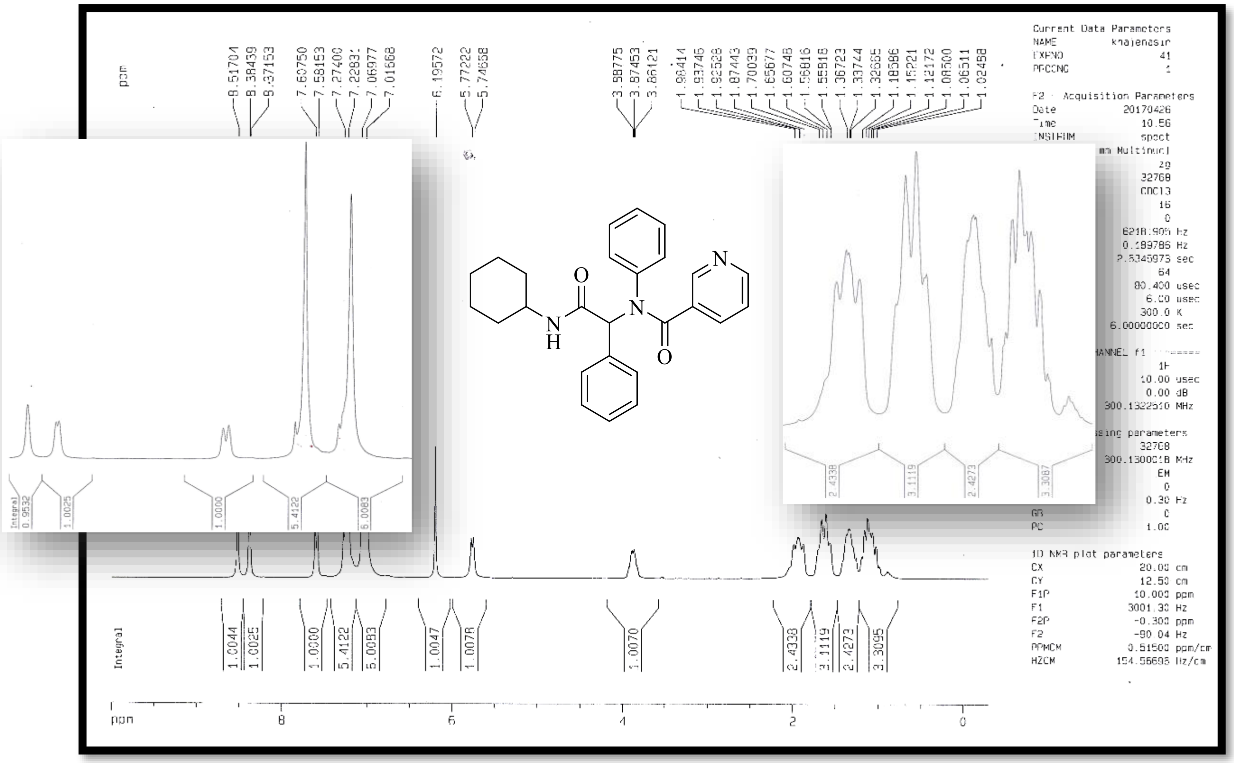

Figure **S10**: ^{1}H-NMR spectra of **7a** (300MHz, $CDCl_3$)

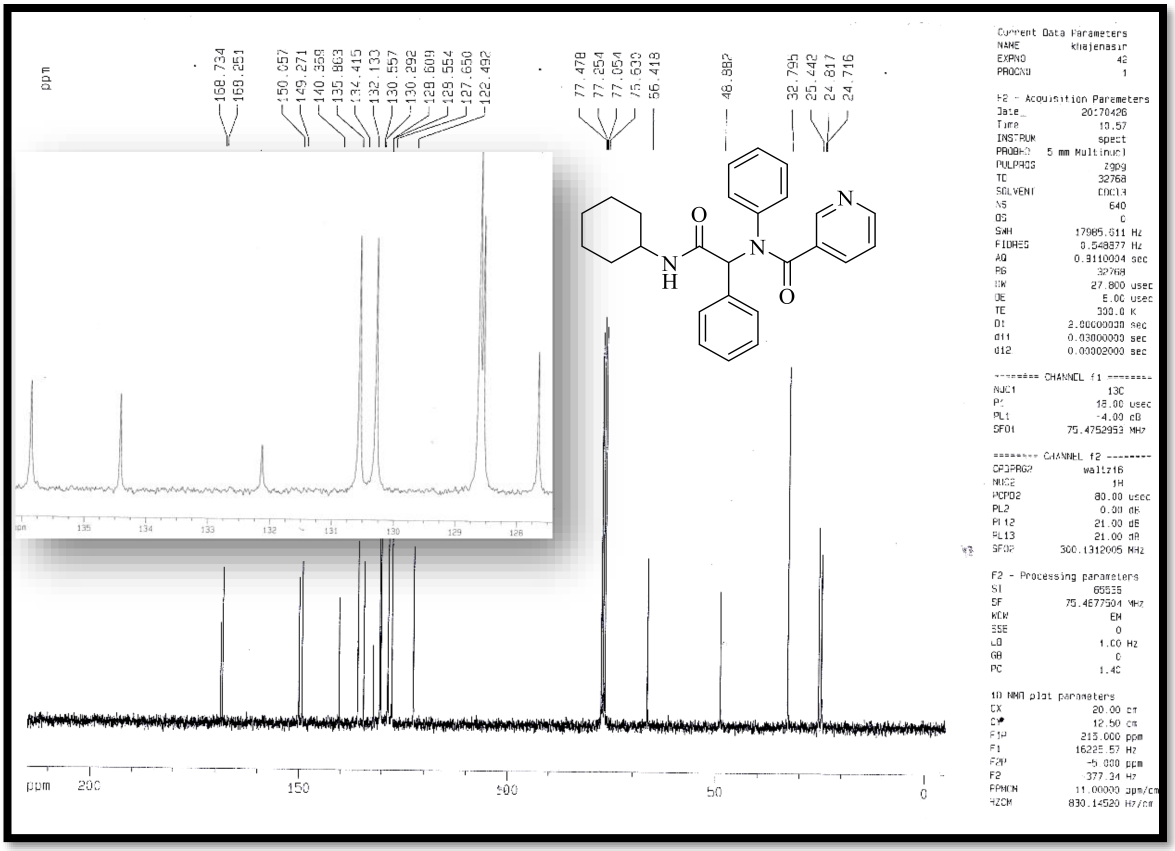

Figure **S11**: ^{13}C-NMR Spectra of **7a** (75 MHz, $CDCl_3$)

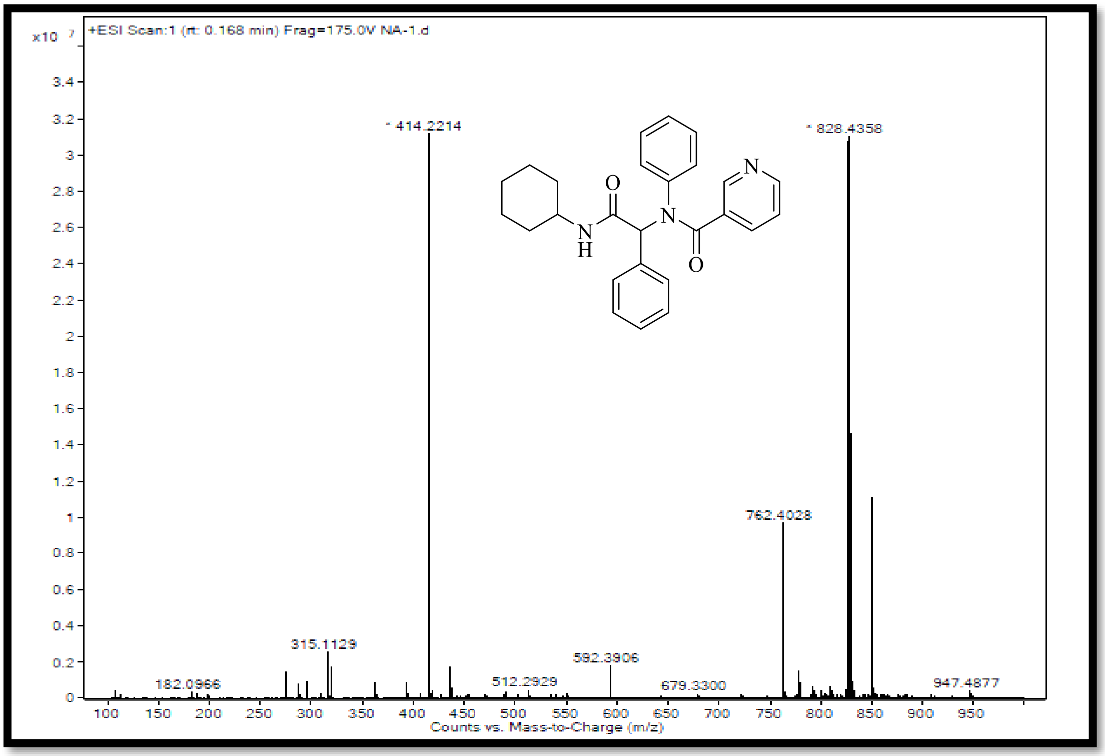

Figure **S12**: HRMS-ESI of **7a** with formula $C_{26}H_{27}N_3O_2$ and molecular weight 413.21 g/mol

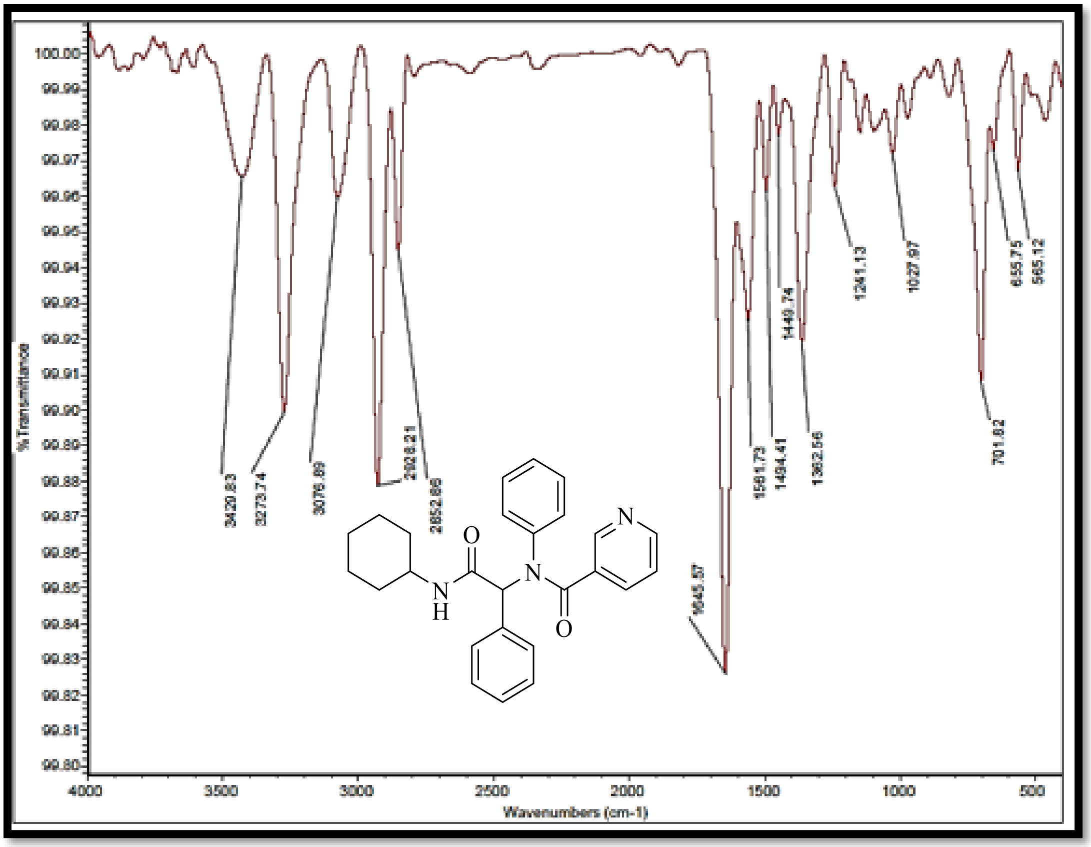

Figure **S13**: IR Spectra of **7a** (KBr, cm-1)

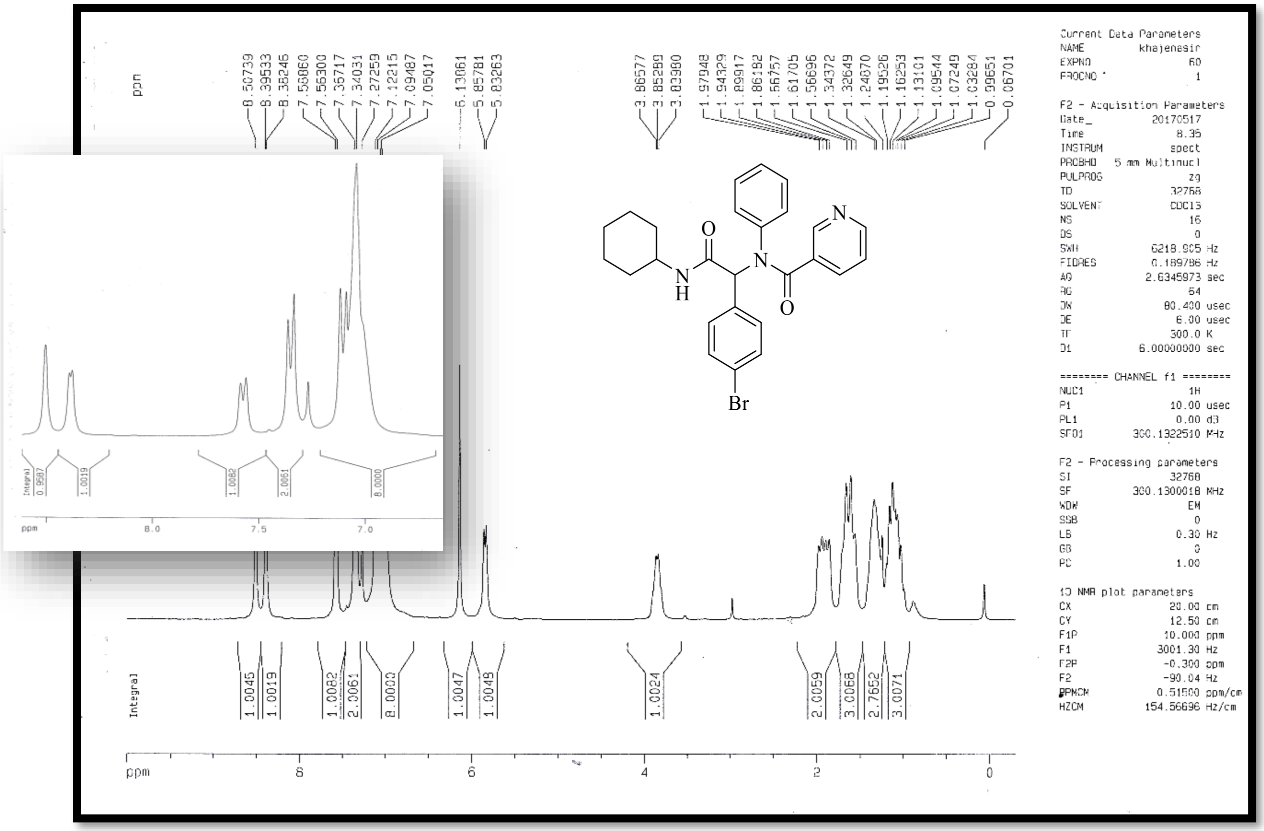

Figure **S14**: ^{1}H-NMR spectra of **7b** (300MHz, $CDCl_3$)

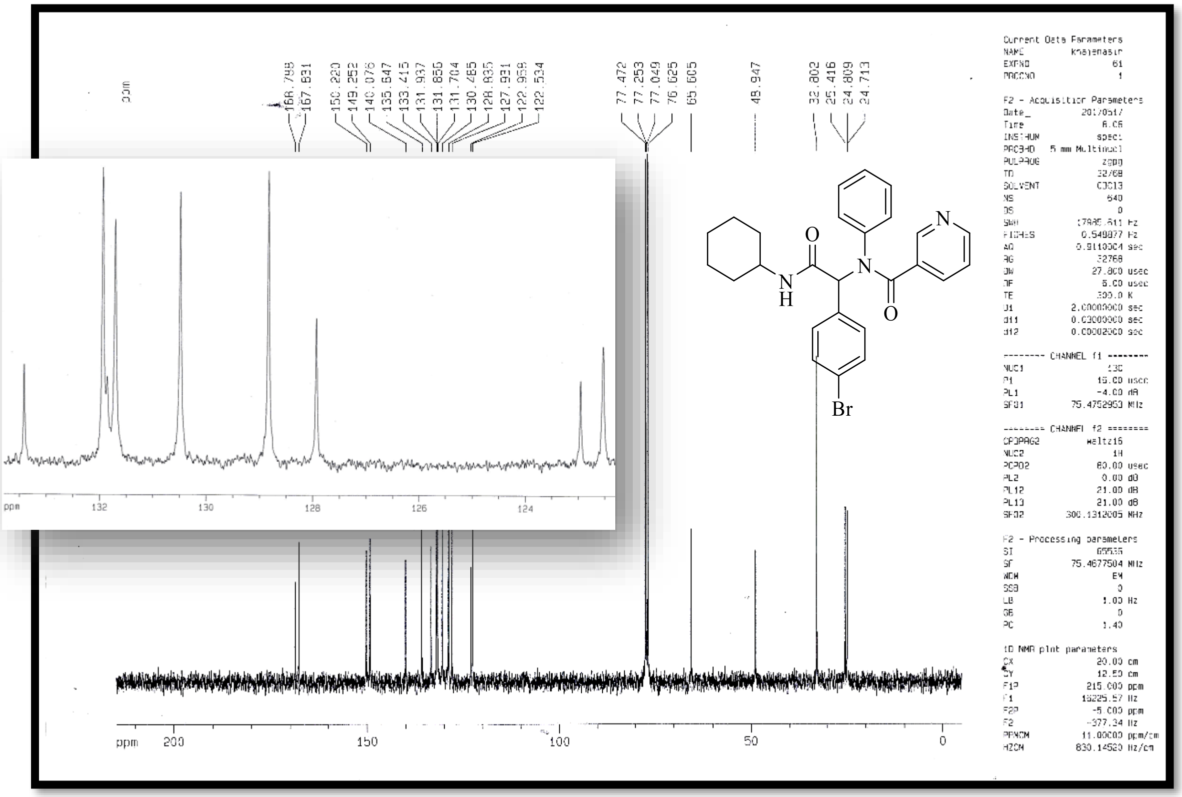

Figure **S15**: ^{13}C-NMR Spectra of **7b** (75 MHz, $CDCl_3$)

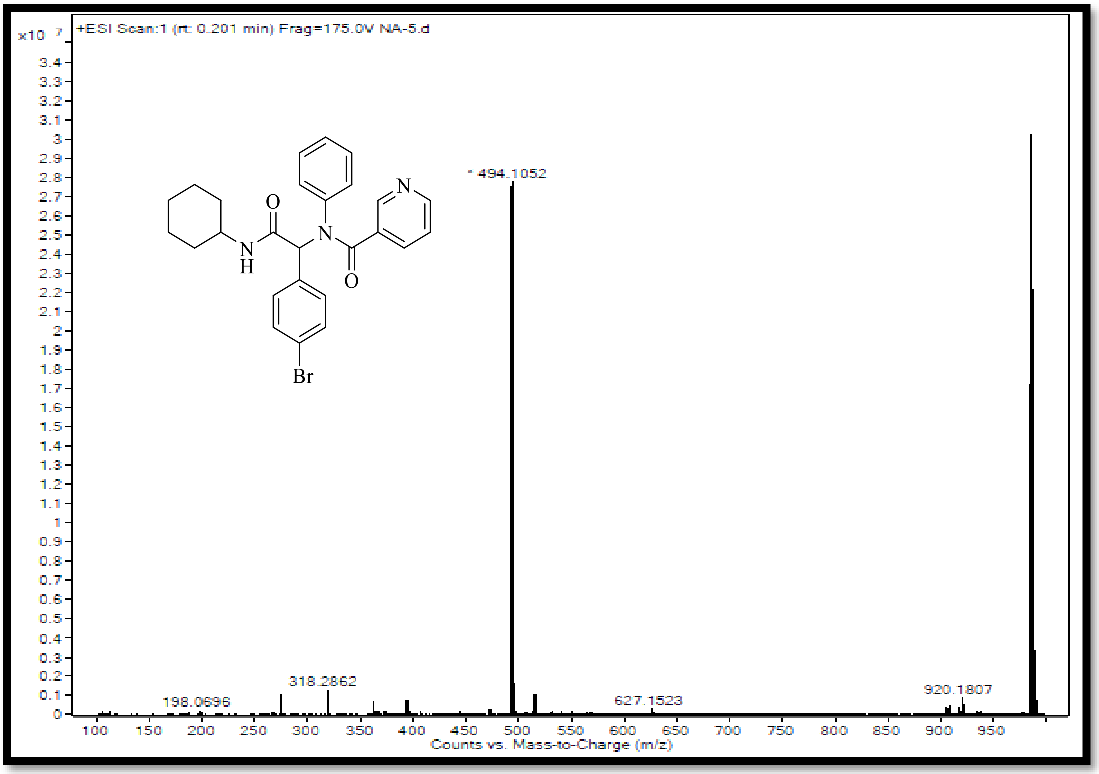

Figure **S16**: HRMS-ESI of **7b** with formula $C_{26}H_{26}BrN_3O_2$ and molecular weight 491.12 g/mol

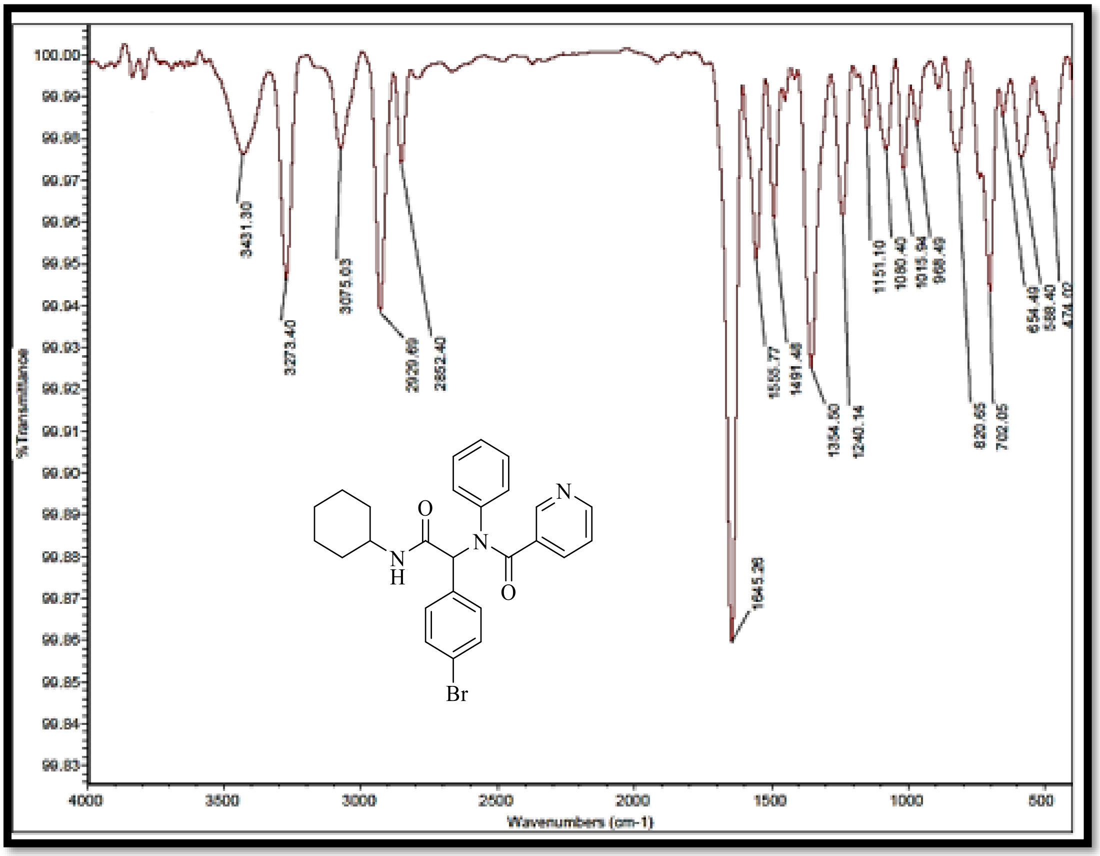

Figure **S17**: IR Spectra of **7b** (KBr, cm-1)

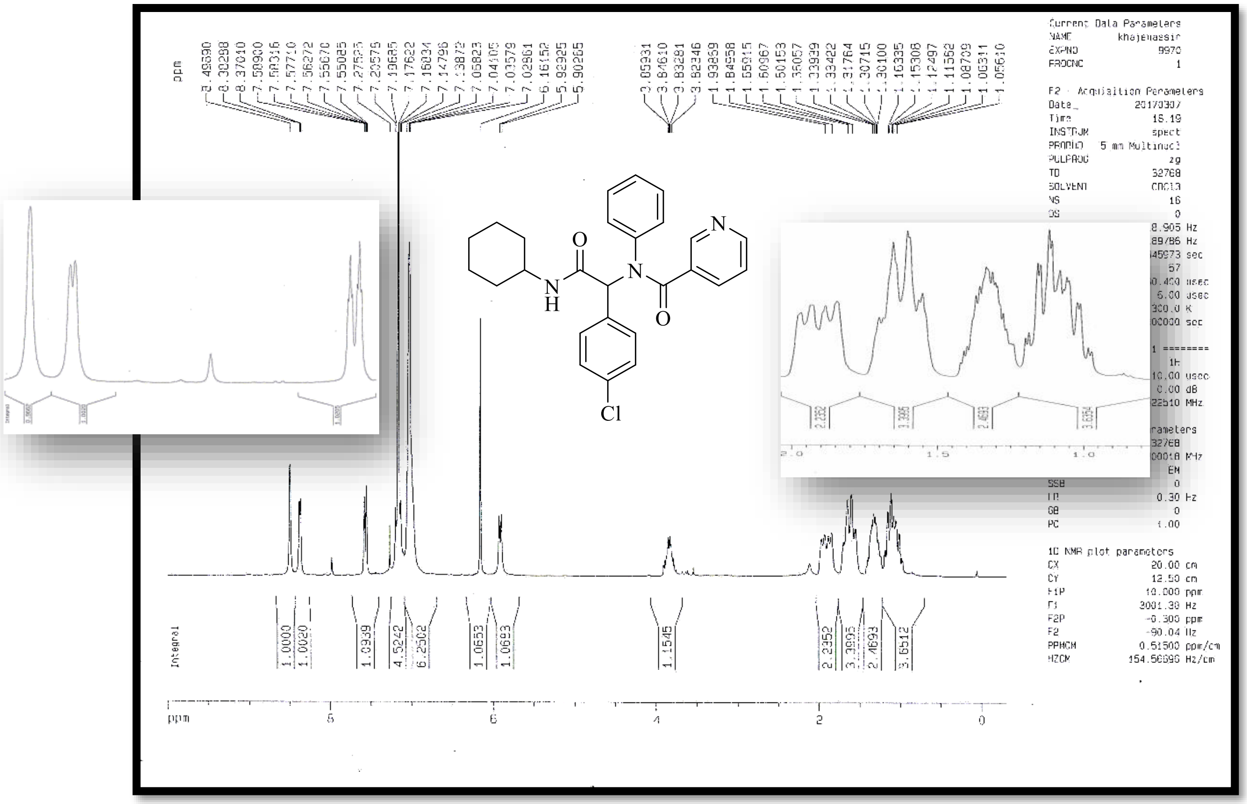

Figure **S18**: ^{1}H-NMR spectra of **7c** (300MHz, $CDCl_3$)

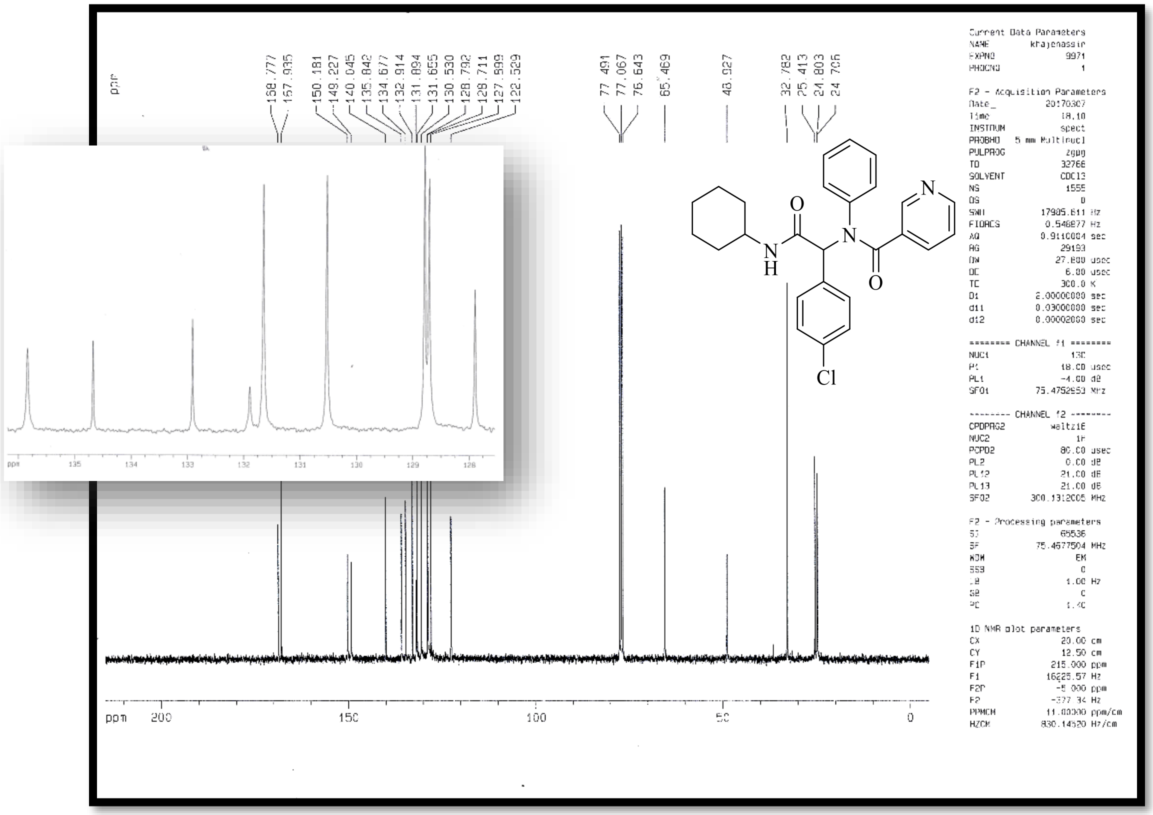

Figure **S19**: ^{13}C-NMR Spectra of **7c** (75 MHz, $CDCl_3$)

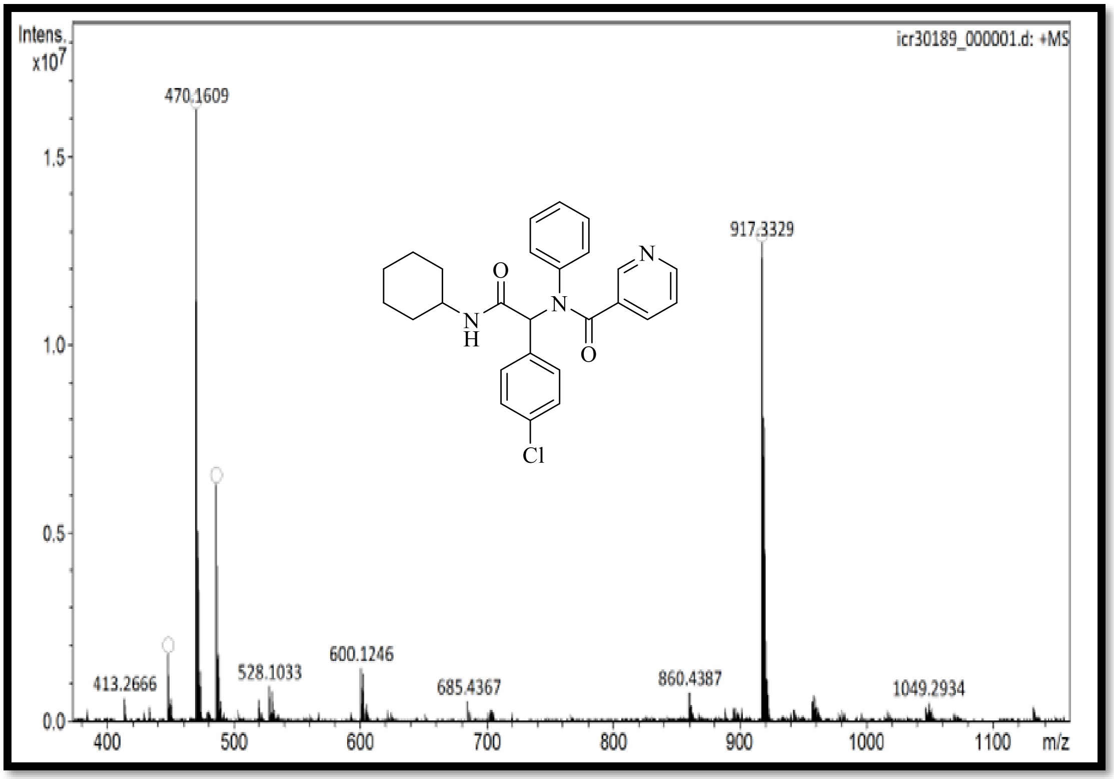

Figure **S20**: HRMS-ESI of **7c** with formula $C_{26}H_{26}ClN_3O_2$ and molecular weight 447.17 g/mol

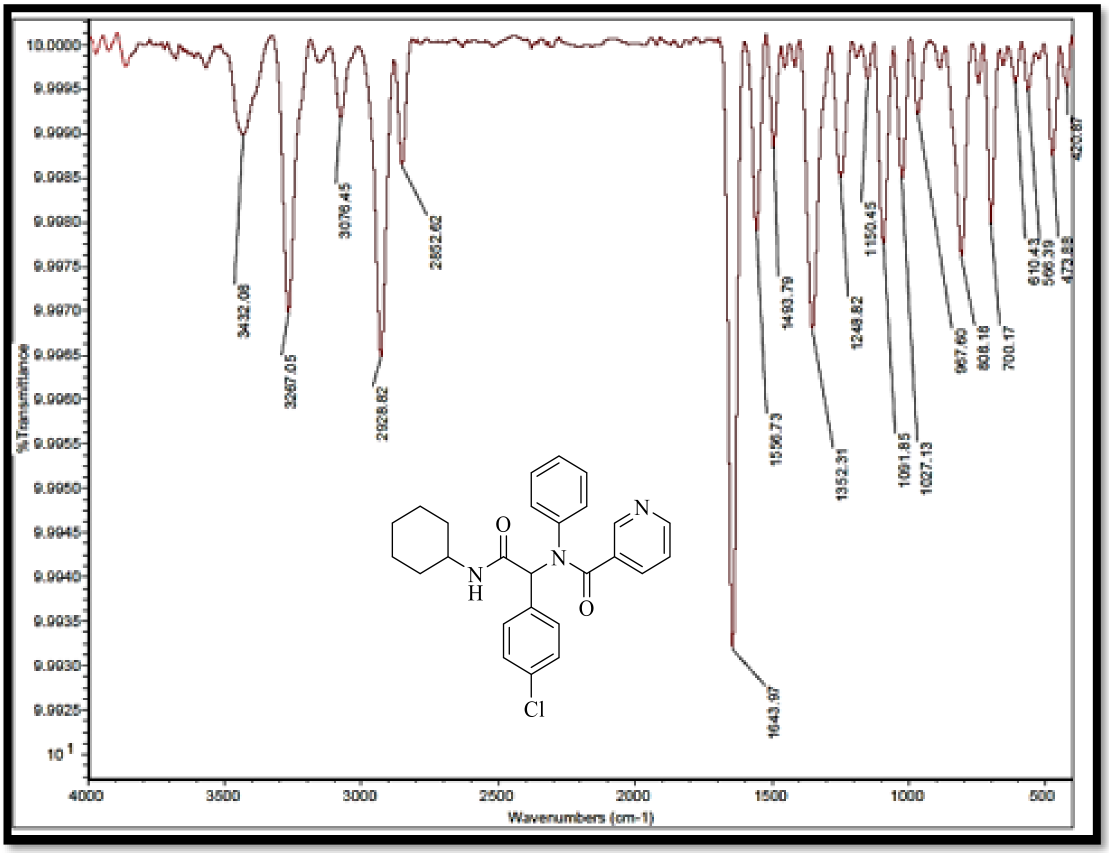

Figure **S21**: IR Spectra of **7c** (KBr, cm-1)

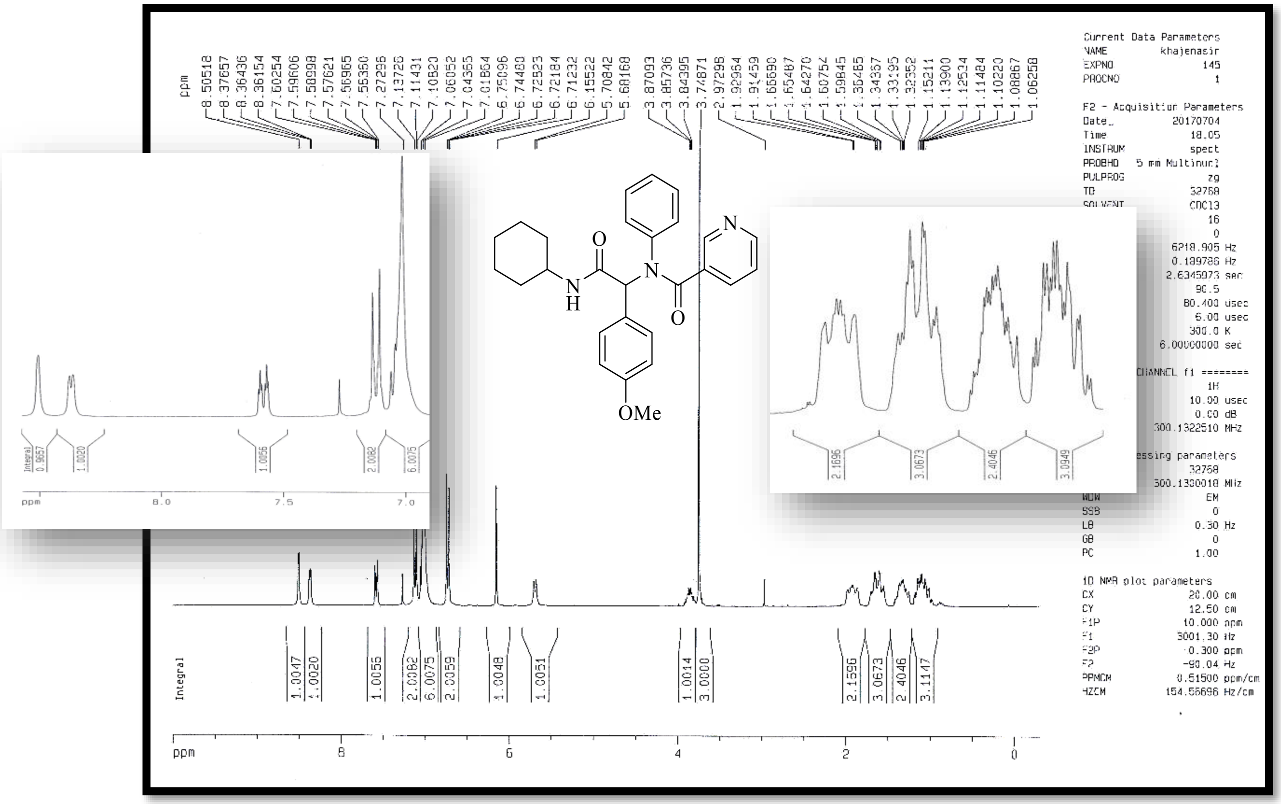

Figure **S22**: ^{1}H-NMR spectra of **7d** (300MHz, $CDCl_3$)

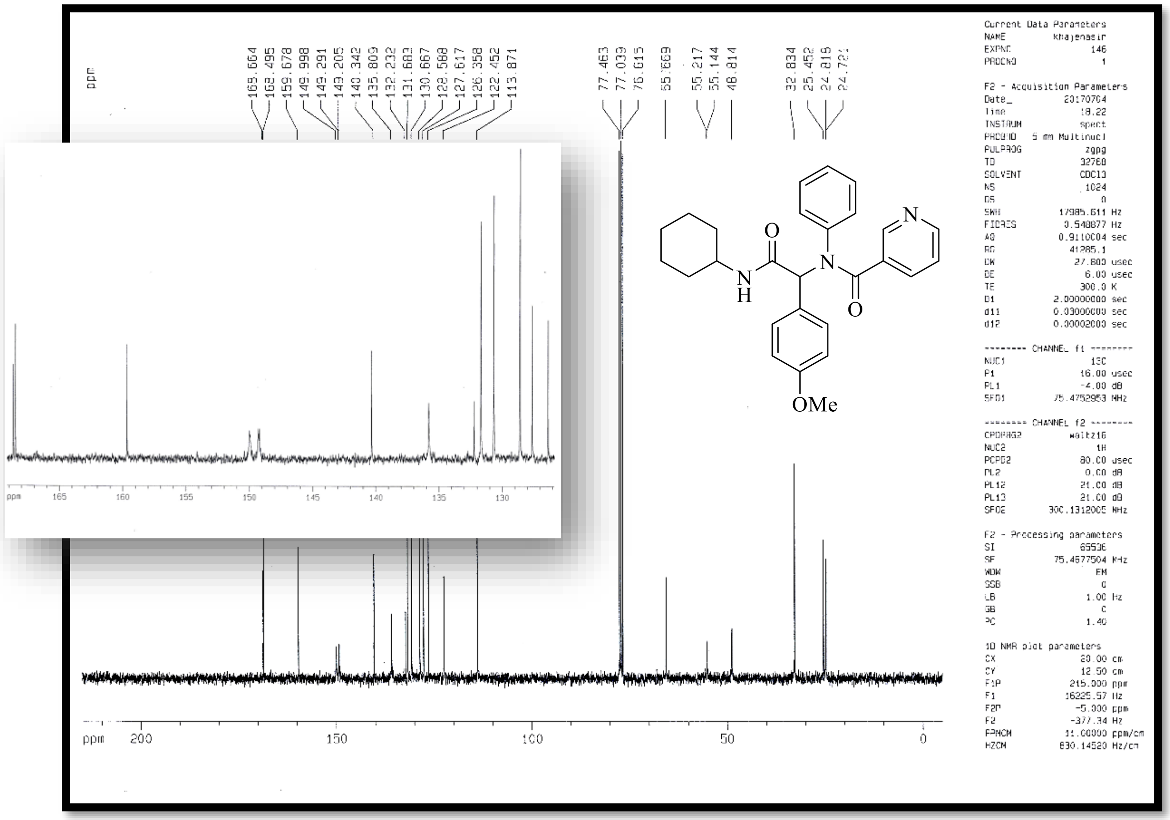

Figure **S23**: ^{13}C-NMR Spectra of **7d** (75 MHz, $CDCl_3$)

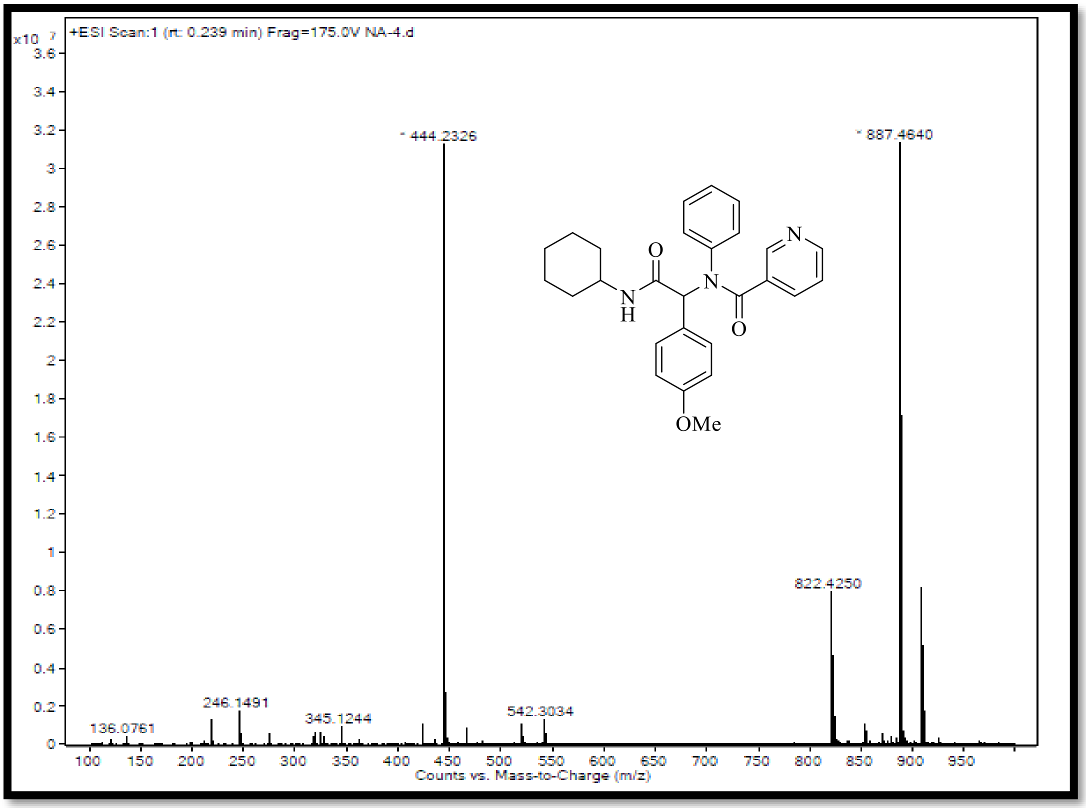

Figure **S24**: HRMS-ESI of **7d** with formula $C_{27}H_{29}N_3O_3$ and molecular weight 443.22 g/mol

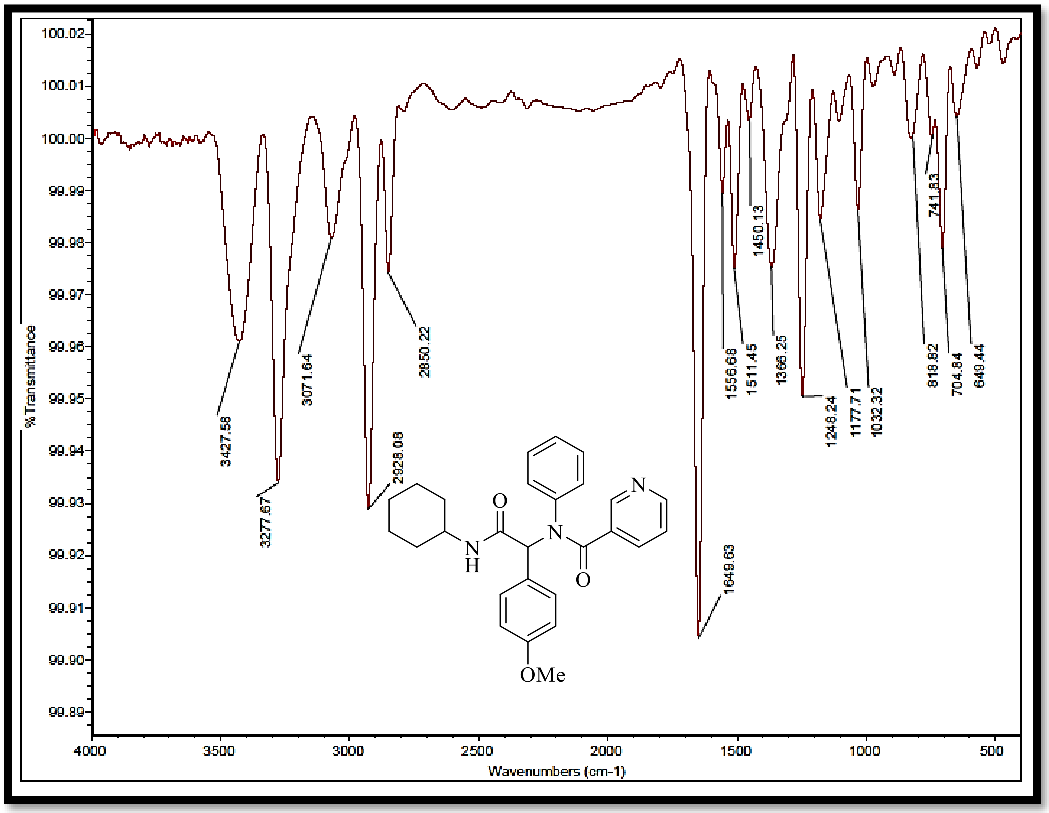

Figure **S25**: IR Spectra of **7d** (KBr, cm^{-1})

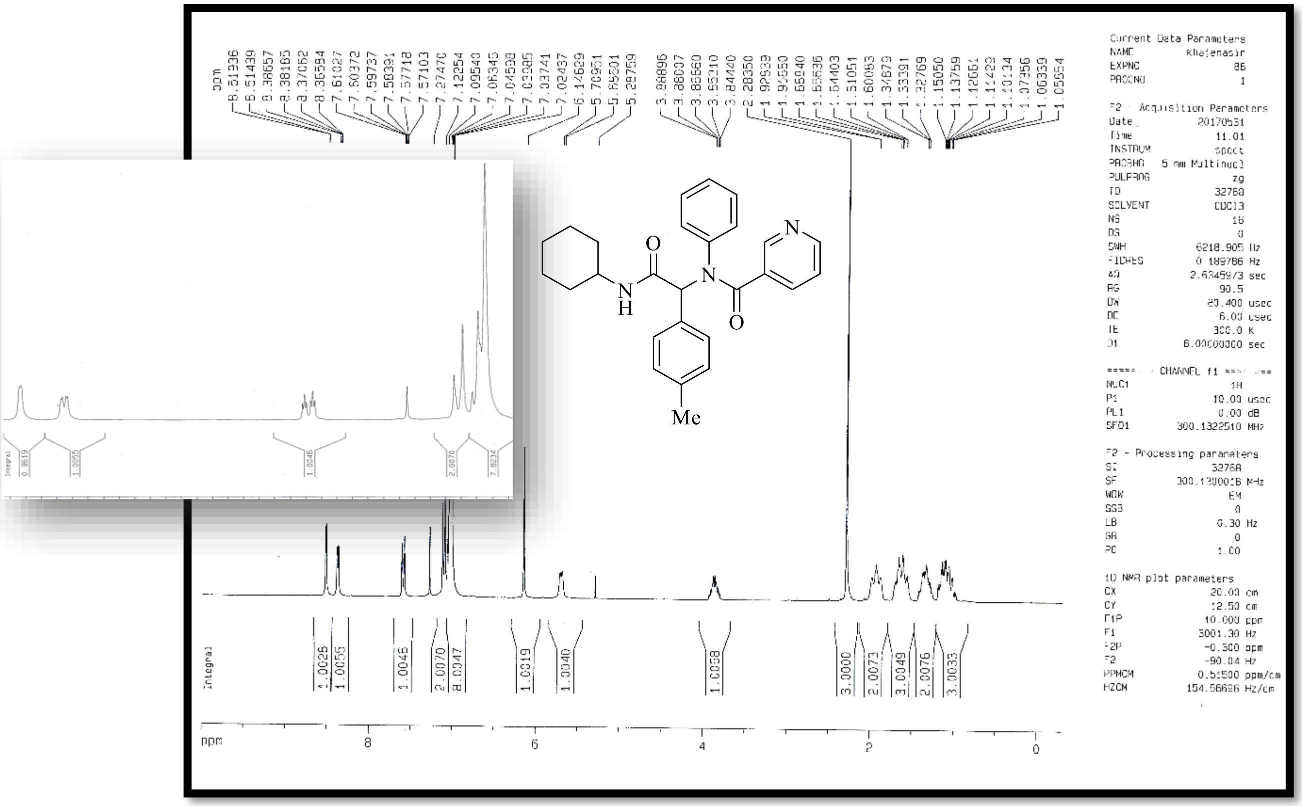

Figure **S26**: ^{1}H-NMR spectra of **7e** (300MHz, $CDCl_3$)

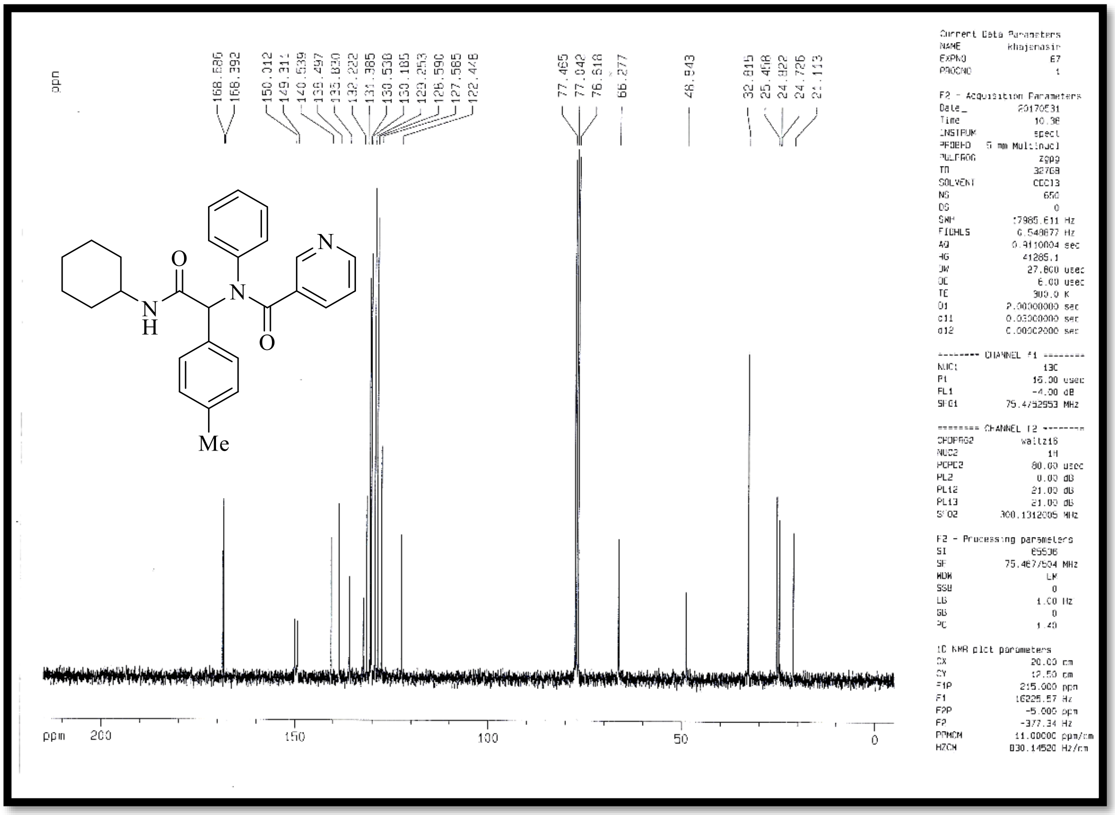

Figure **S27**: ^{13}C-NMR Spectra of **7e** (75 MHz, $CDCl_3$)

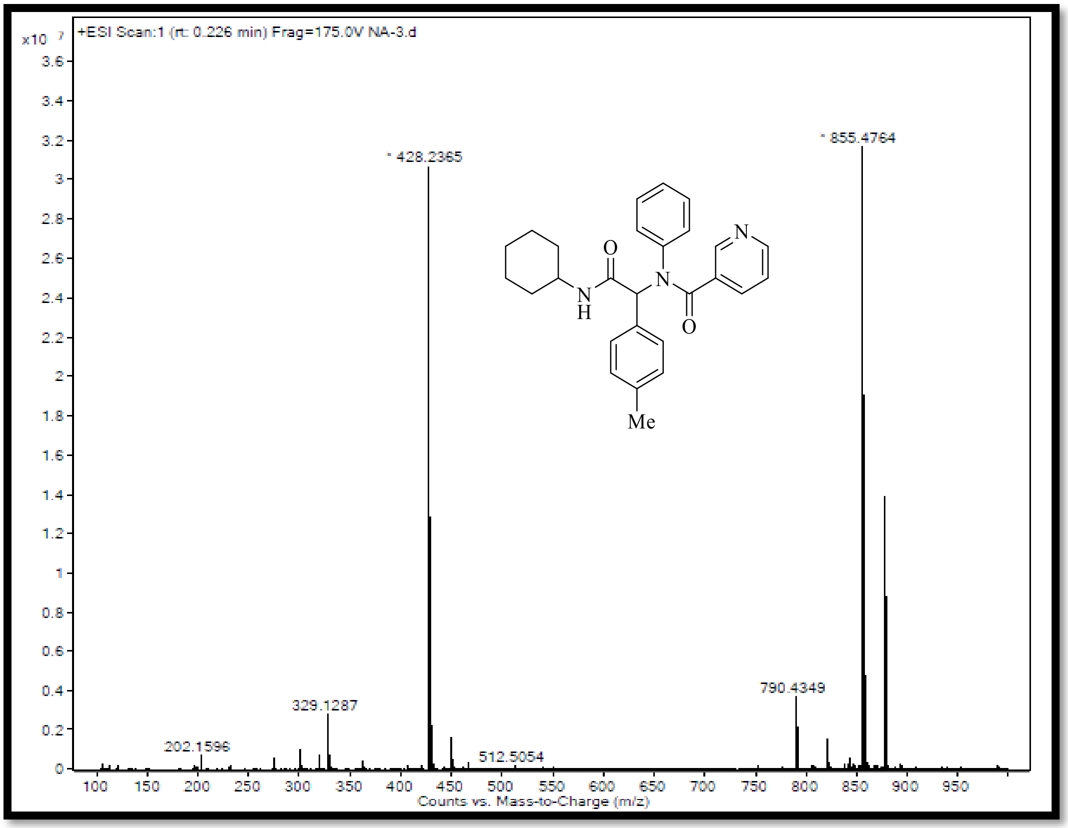

Figure **S28**: HRMS-ESI of **7e** with formula $C_{27}H_{29}N_3O_2$ and molecular weight 427.22 g/mol

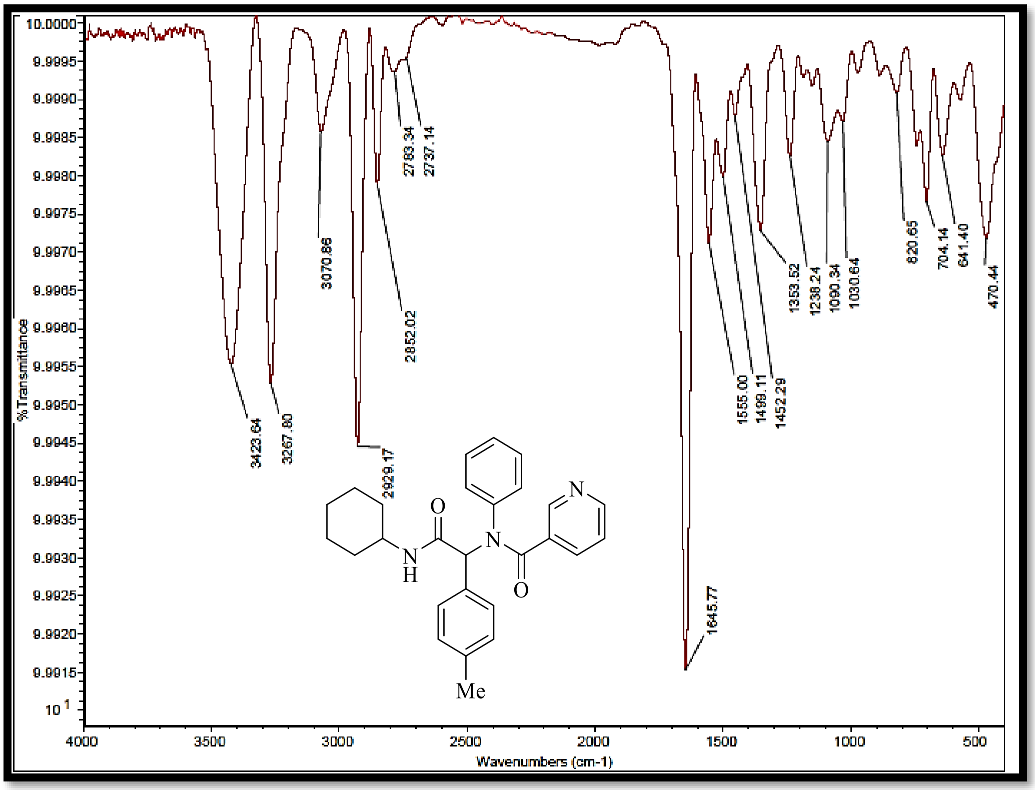

Figure **S29**: IR Spectra of **7e** (KBr, cm^{-1})

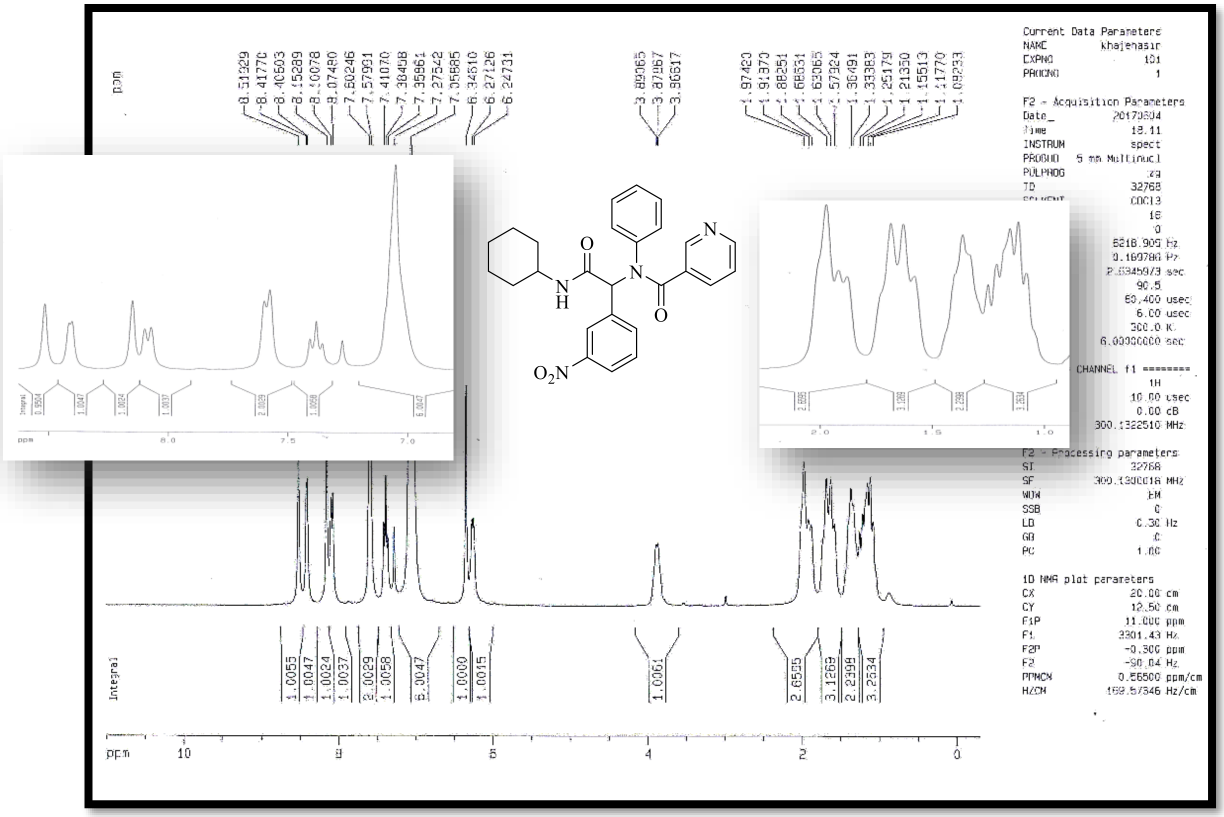

Figure **S30**: ^{1}H-NMR spectra of **7f** (300MHz, CDCl3)

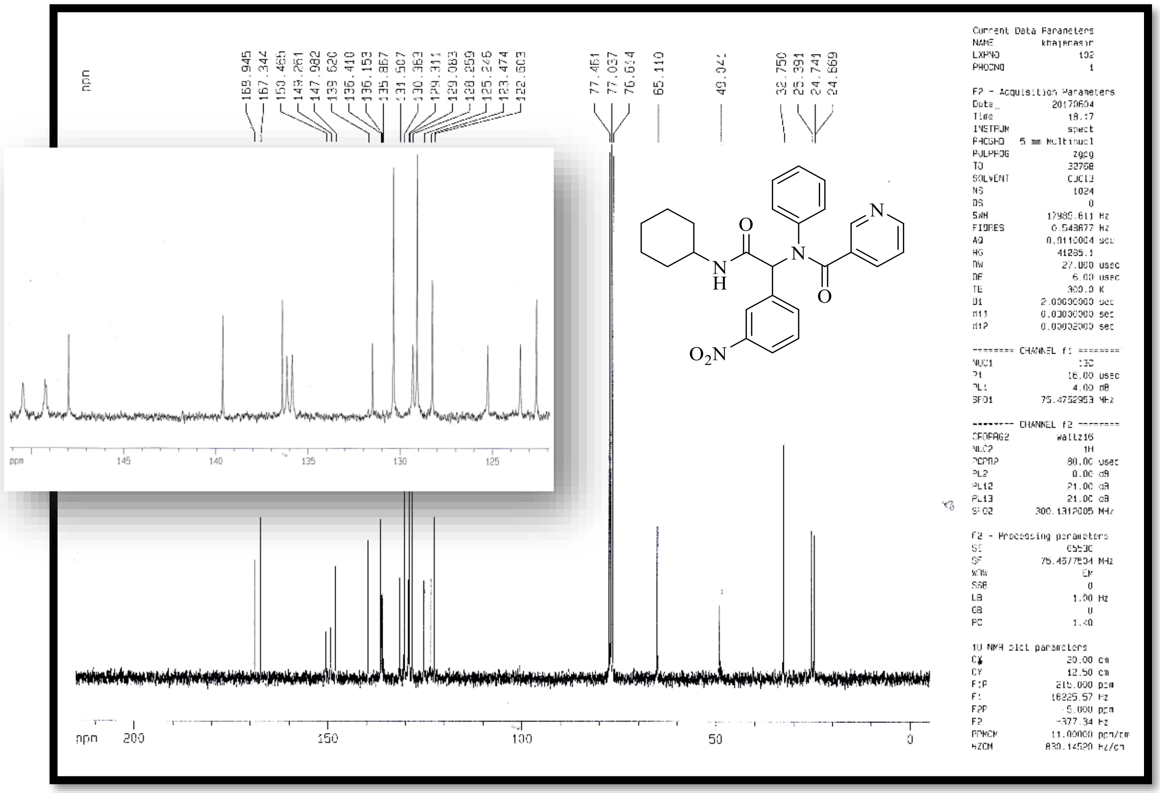

Figure **S31**: ^{13}C-NMR Spectra of **7f** (75 MHz, CDCl3)

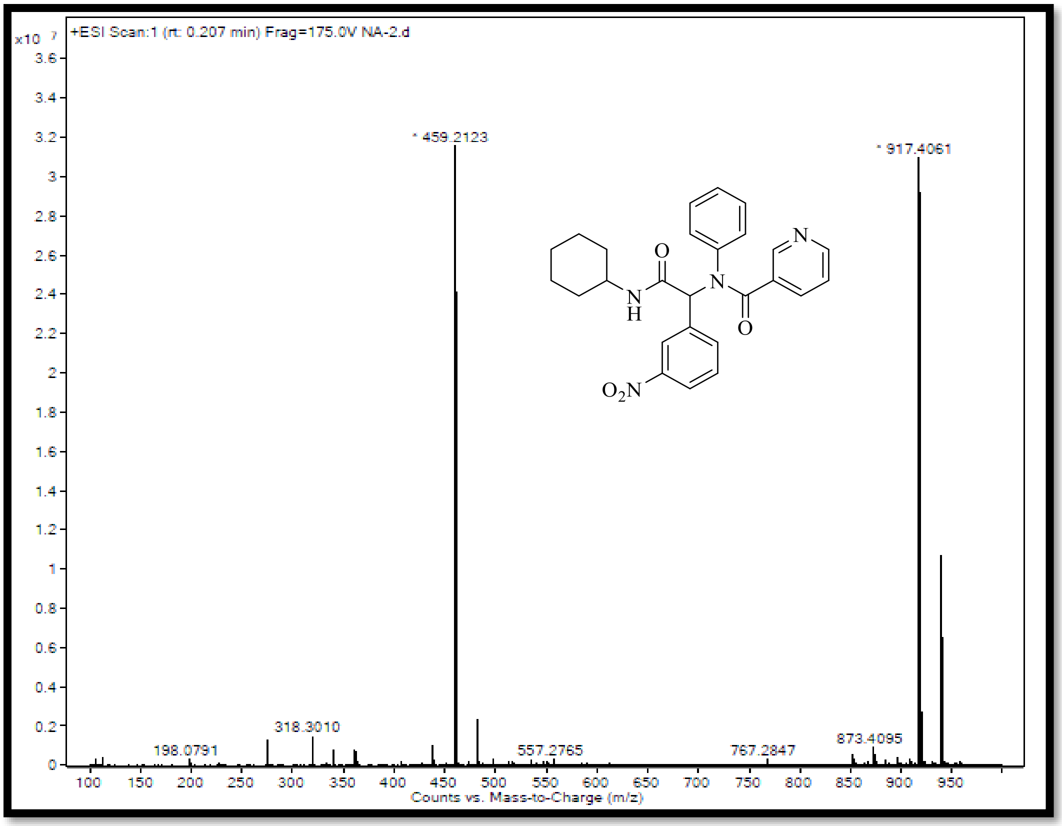

Figure **S32**: HRMS-ESI of **7f** with formula $C_{26}H_{26}N_4O_4$ and molecular weight 458.19 g/mol

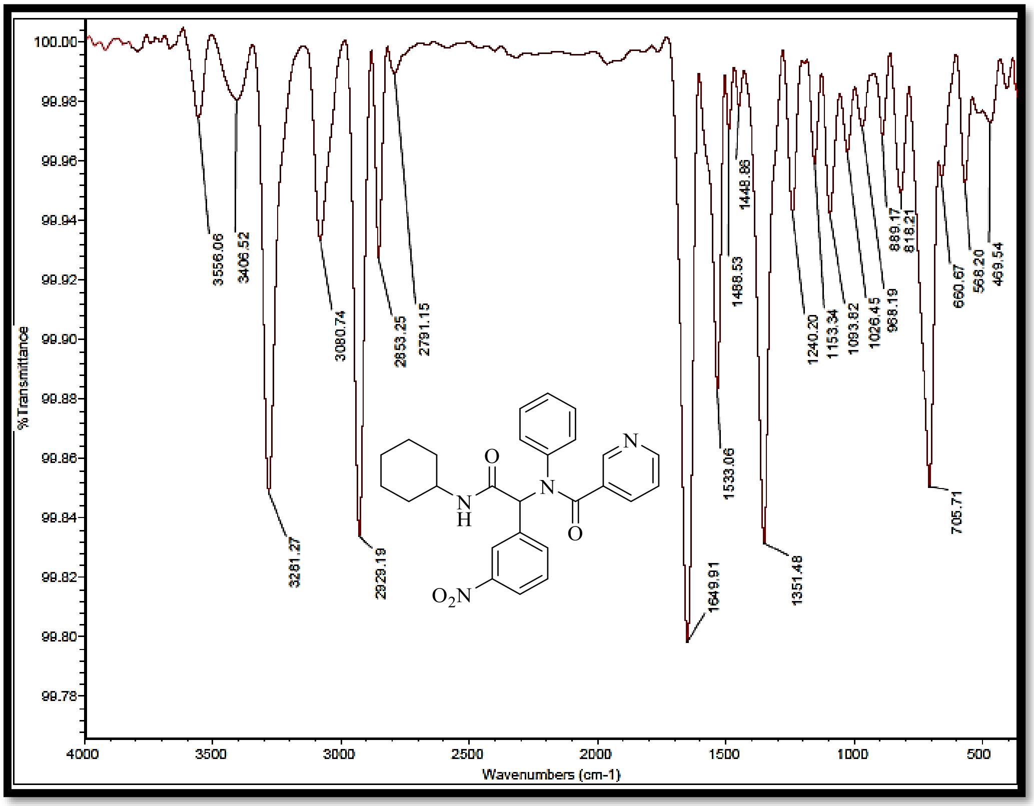

Figure **S33**: IR Spectra of **7f** (KBr, cm-1)

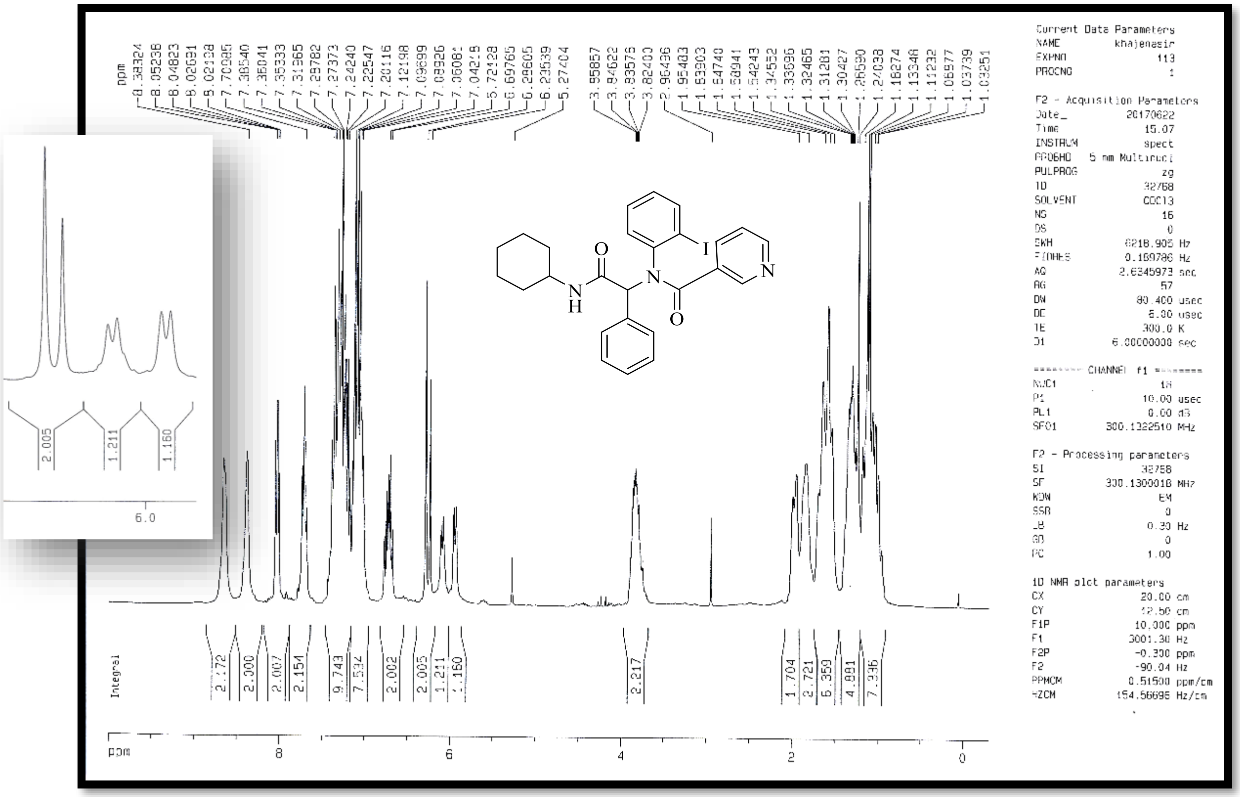

Figure **S34**: ^{1}H-NMR spectra of **7g** (300MHz, $CDCl_3$)

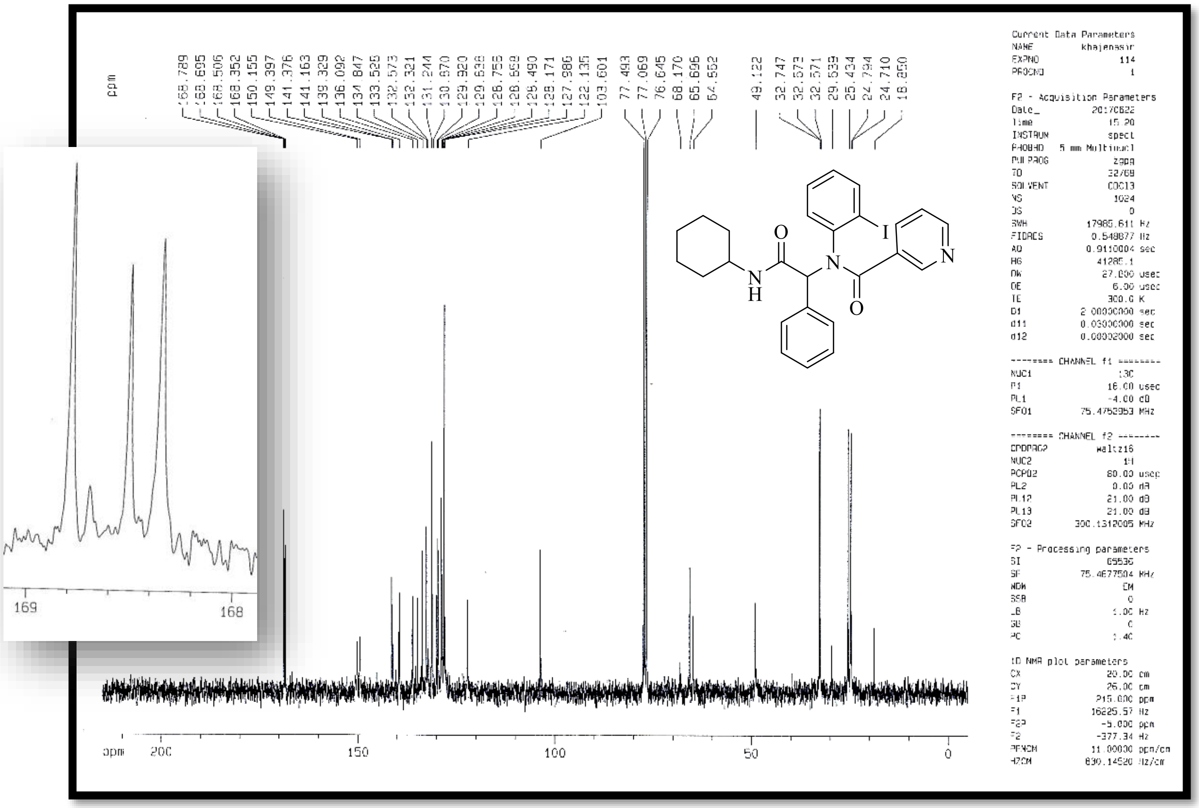

Figure **S35**: ^{13}C-NMR Spectra of **7g** (75 MHz, $CDCl_3$)

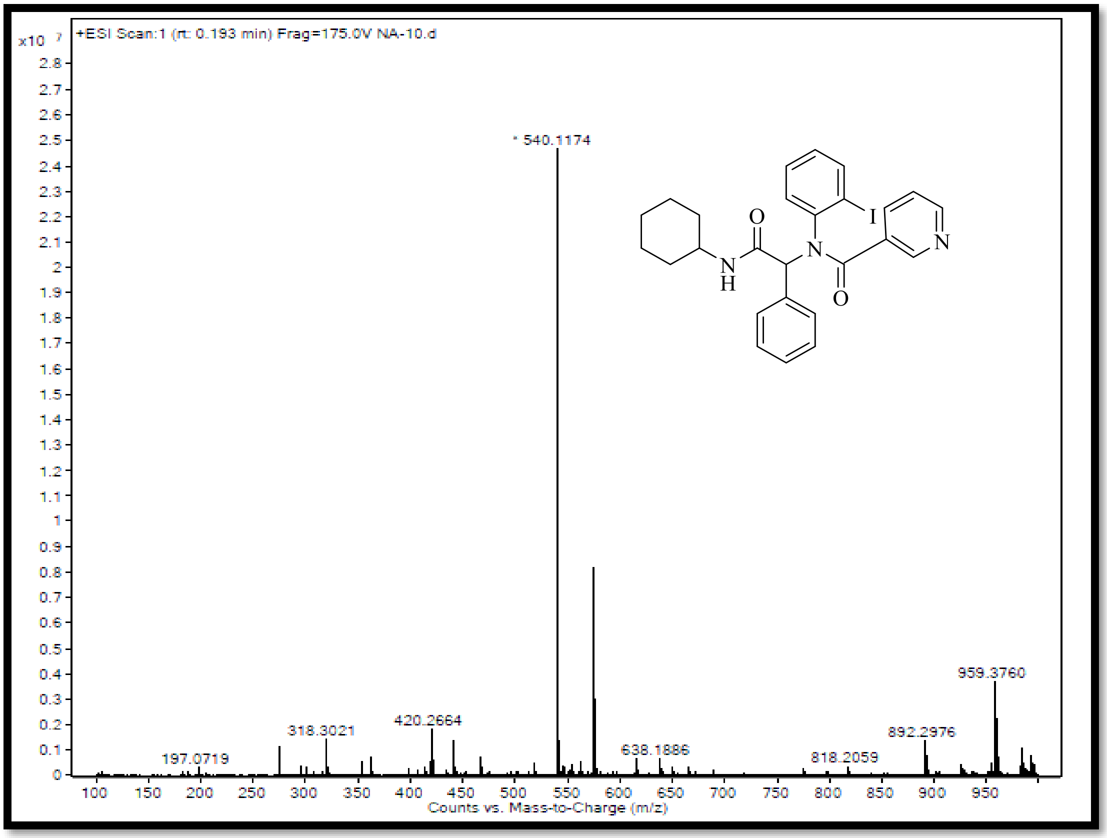

Figure **S36**: HRMS-ESI of **7g** with formula $C_{26}H_{26}IN_3O_2$ and molecular weight 539.10 g/mol

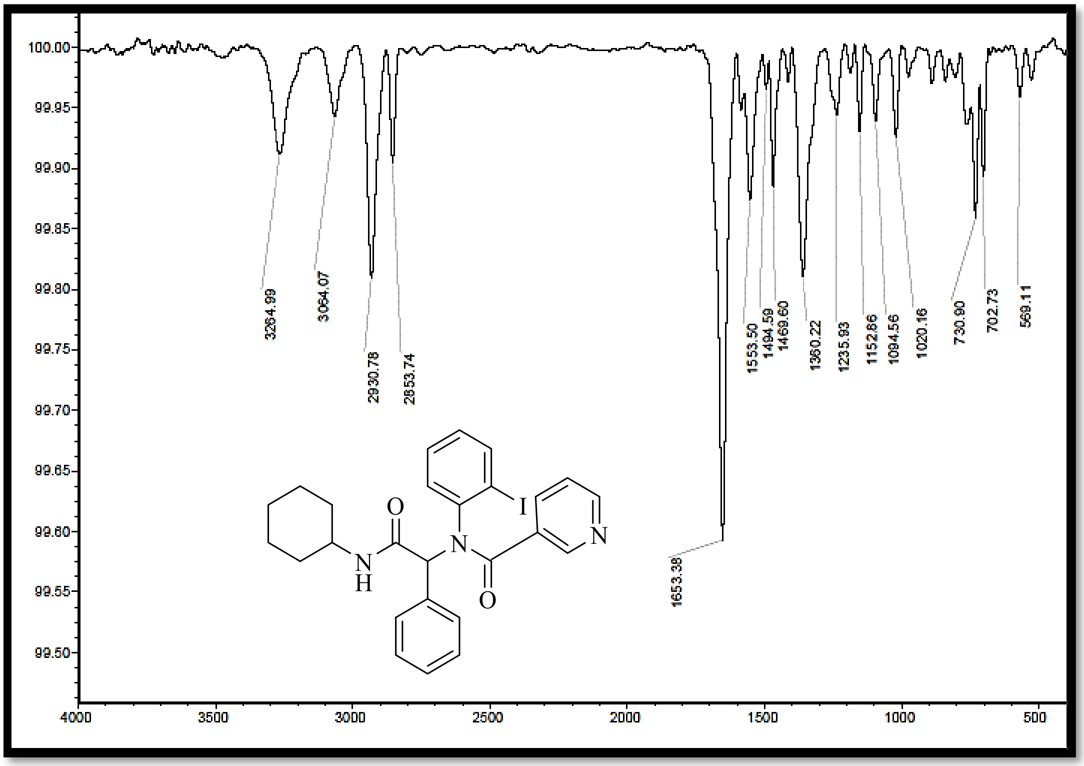

Figure **S37**: IR Spectra of **7g** (KBr, cm-1)

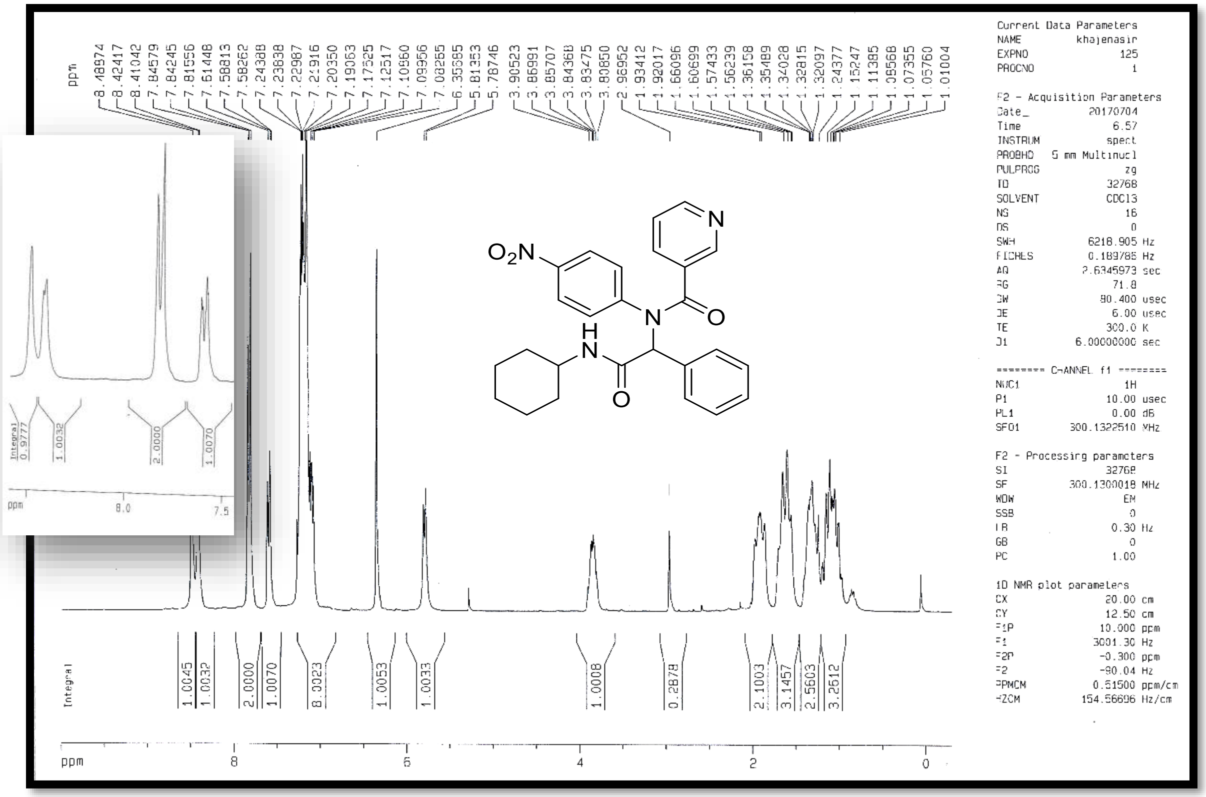

Figure **S38**: ^{1}H-NMR spectra of **7h** (300MHz, CDCl3)

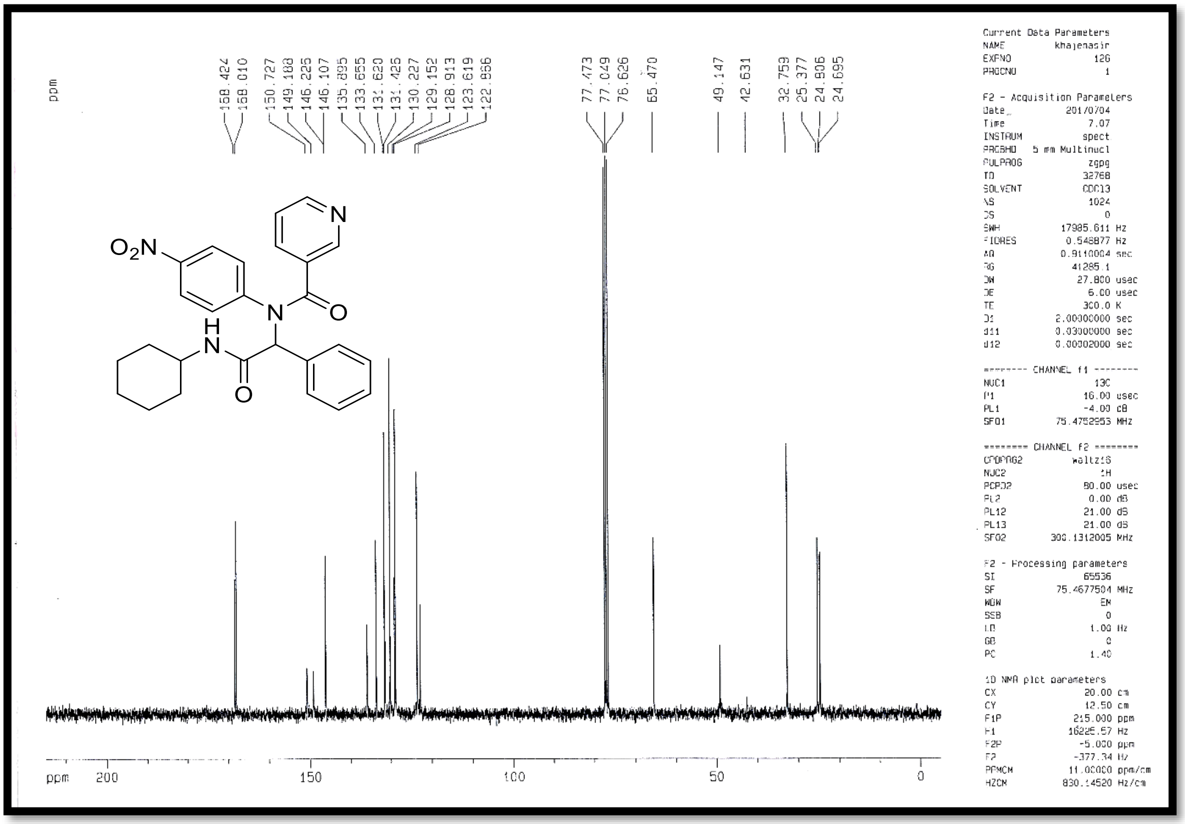

Figure **S39**: ^{13}C-NMR Spectra of **7h** (75 MHz, CDCl3)

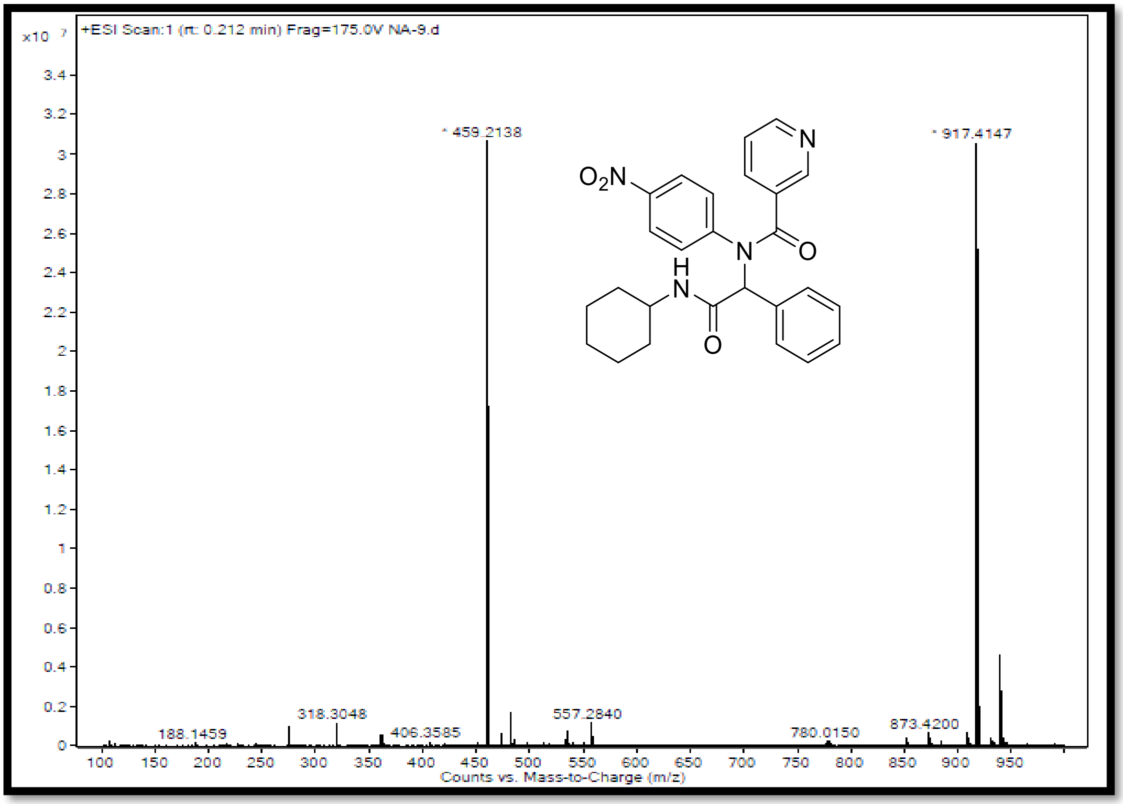

Figure **S40**: HRMS-ESI of **7h** with formula $C_{26}H_{26}N_4O_4$ and molecular weight 458.19 g/mol

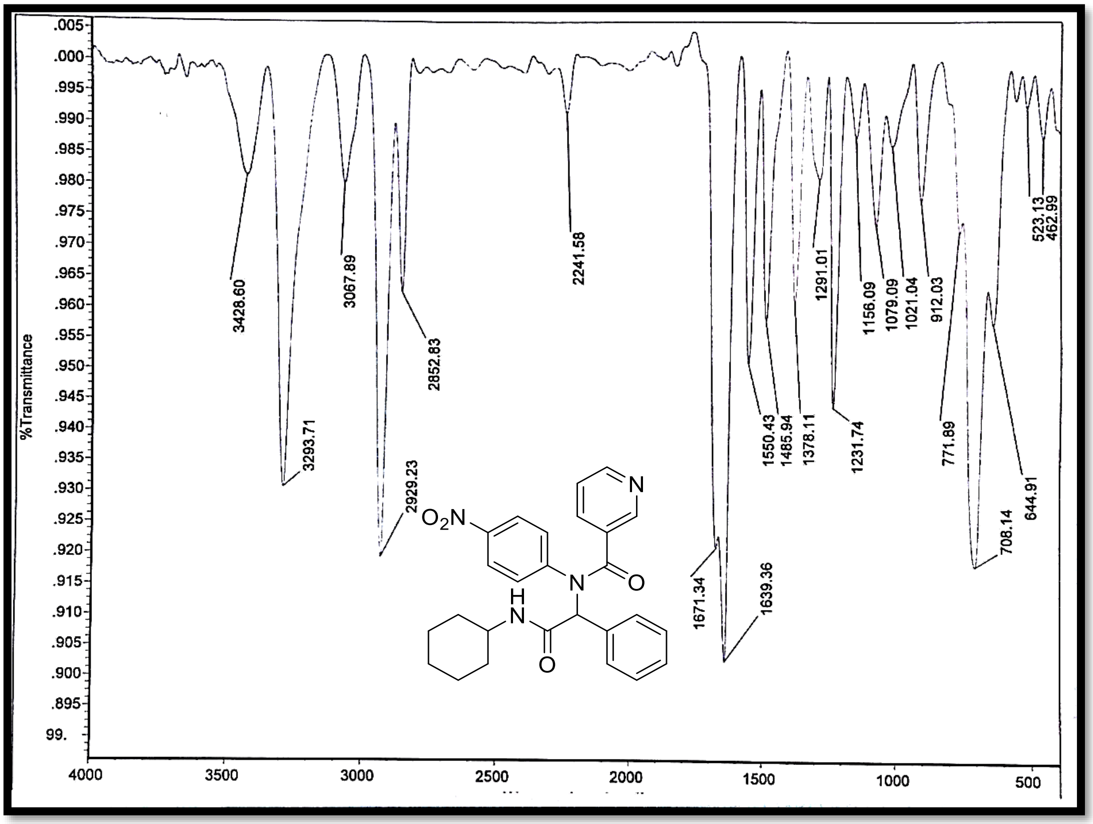

Figure **S41**: IR Spectra of **7h** (KBr, cm^{-1})

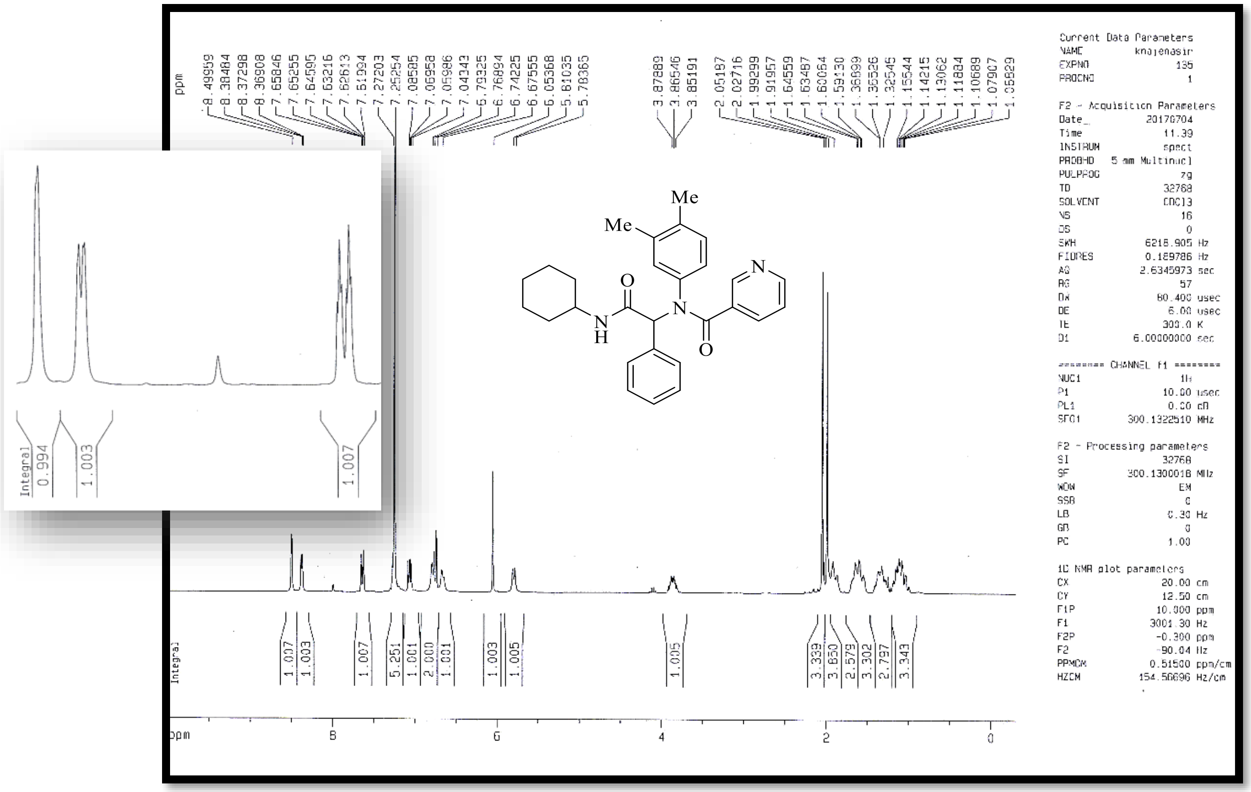

Figure **S42**: ^{1}H-NMR spectra of **7i** (300MHz, $CDCl_3$)

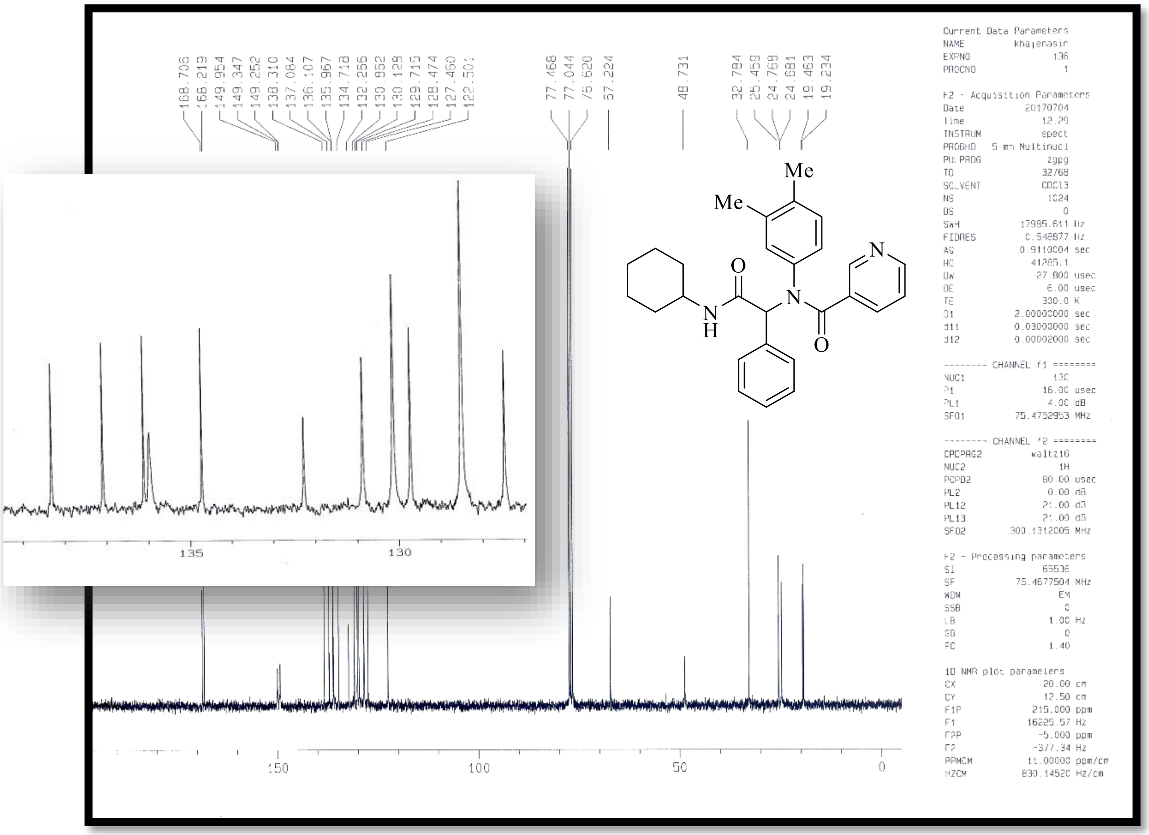

Figure **S43**: ^{13}C-NMR Spectra of **7i** (75 MHz, $CDCl_3$)

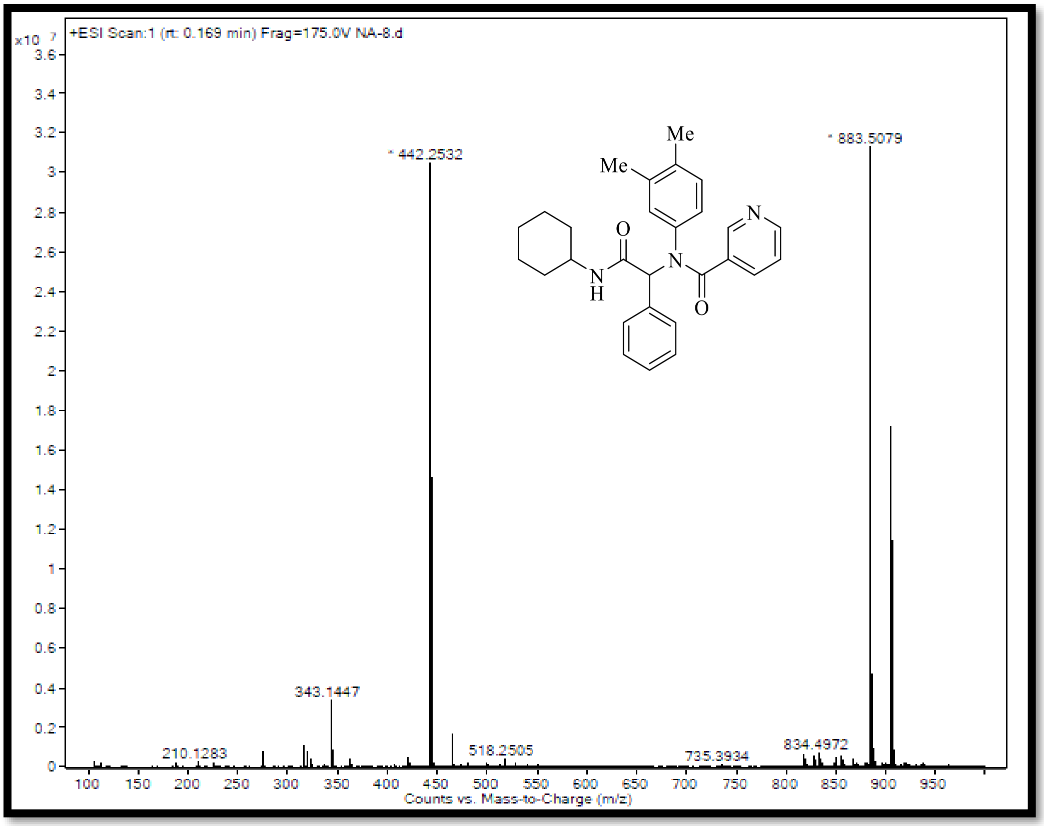

Figure **S44**: HRMS-ESI of **7i** with formula $C_{28}H_{31}N_3O_2$ and molecular weight 441.24 g/mol

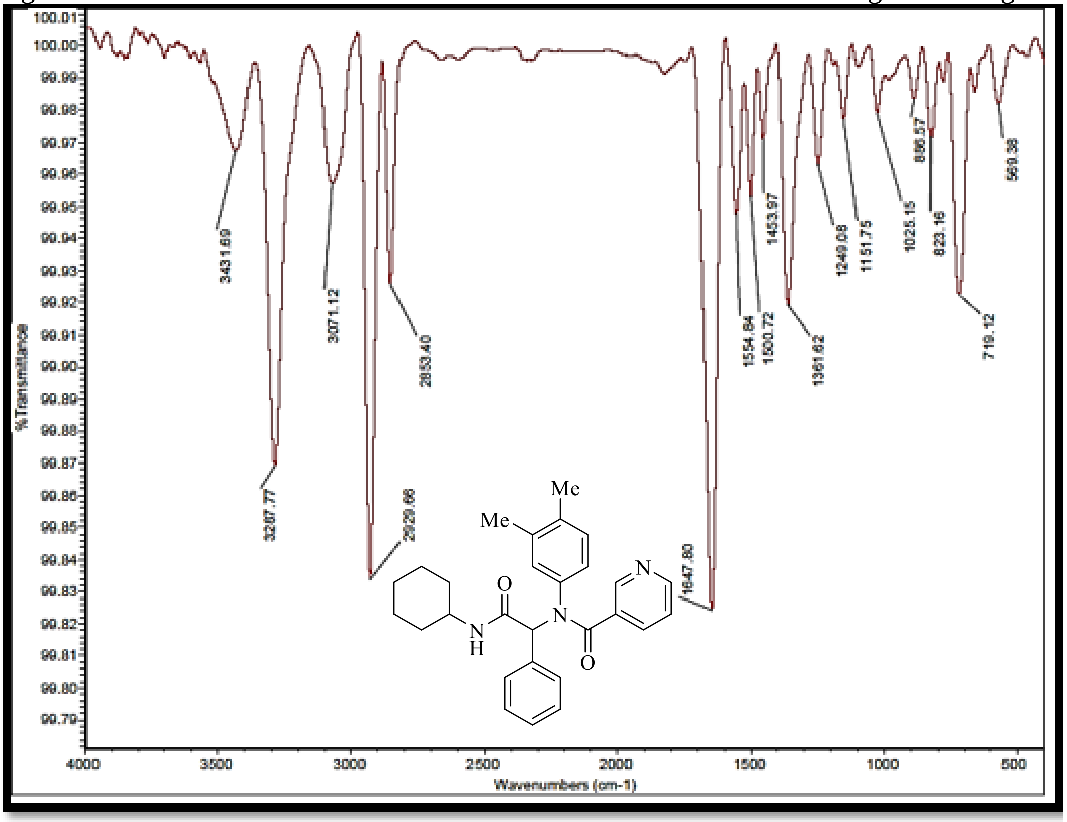

Figure **S45**: IR Spectra of **7i** (KBr, cm-1)

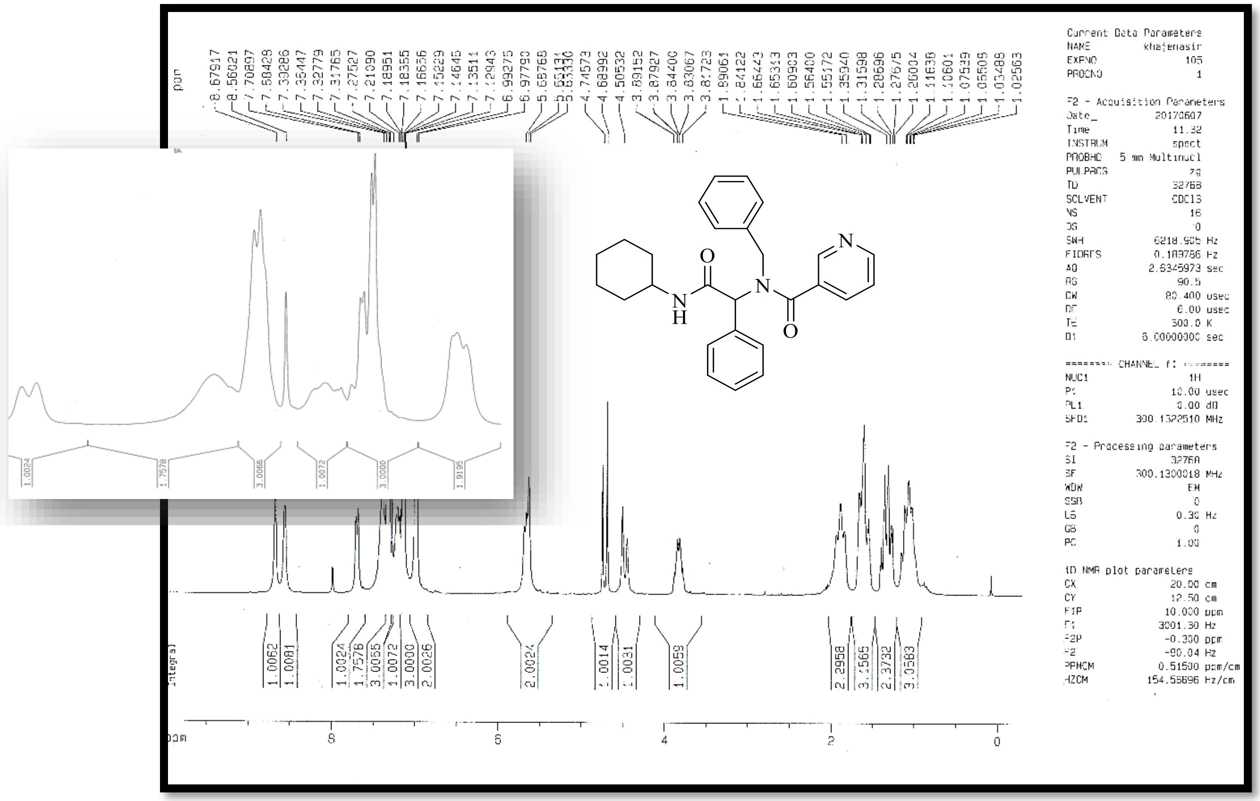

Figure **S46**: ^{1}H-NMR spectra of **7j** (300MHz, $CDCl_3$)

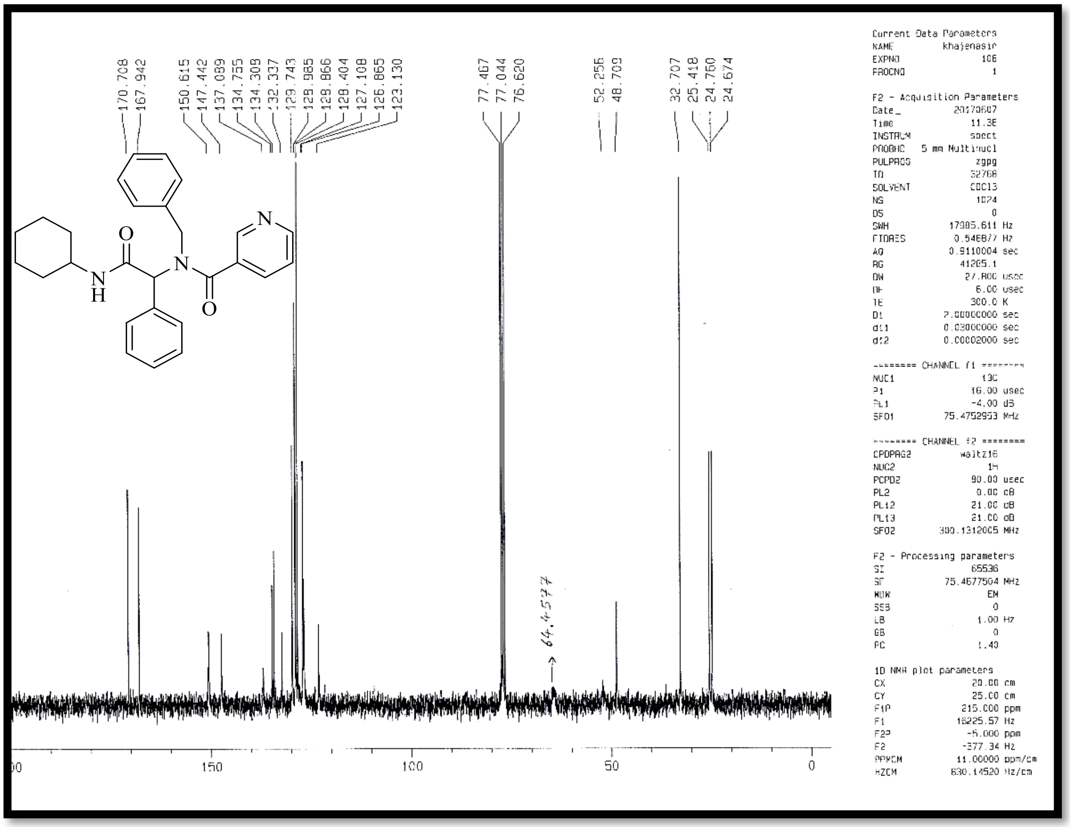

Figure **S47**: ^{13}C-NMR Spectra of **7j** (75 MHz, $CDCl_3$)

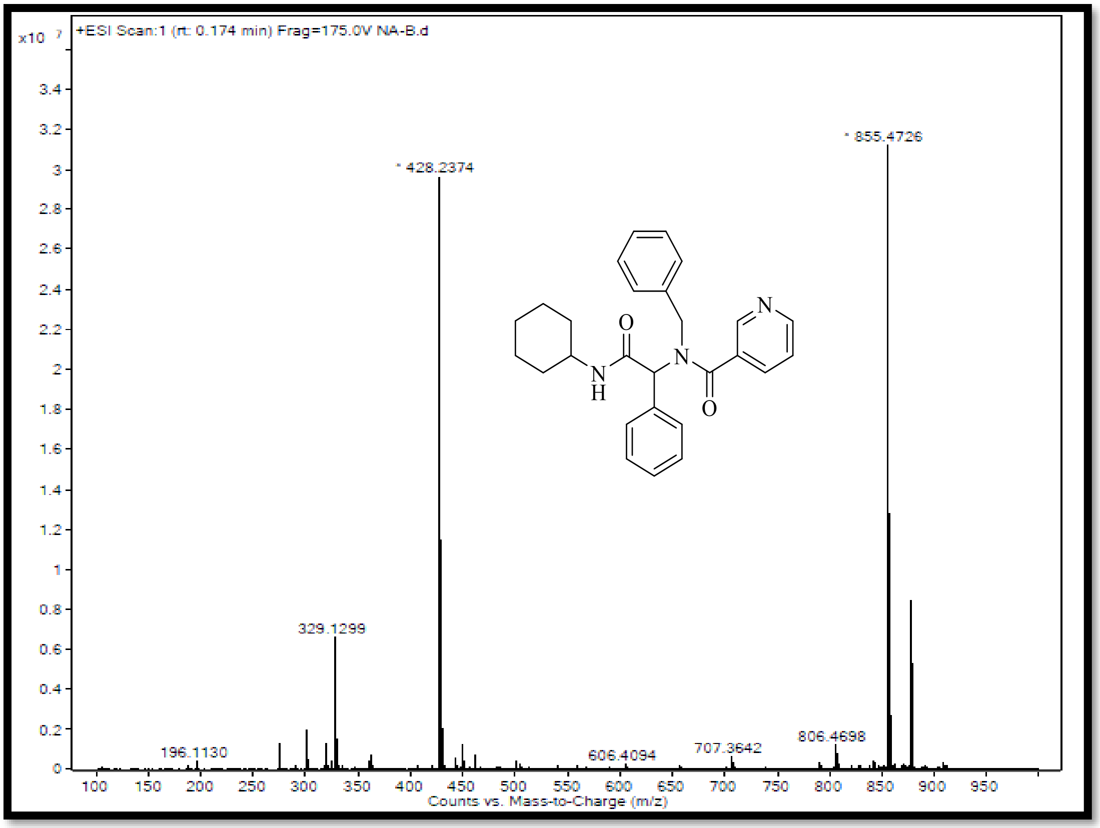

Figure **S48**: HRMS-ESI of **7j** with formula $C_{27}H_{29}N_3O_2$ and molecular weight 427.22 g/mol

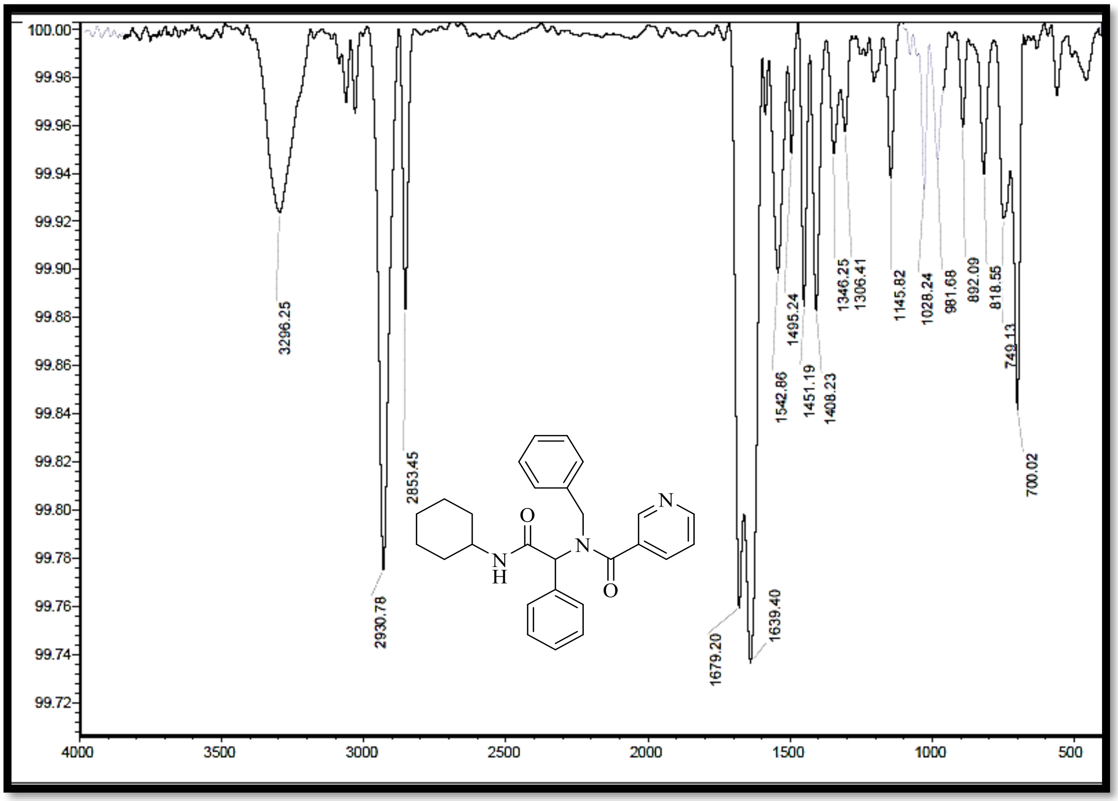

Figure **S49**: IR Spectra of **7j** (KBr, cm^{-1})

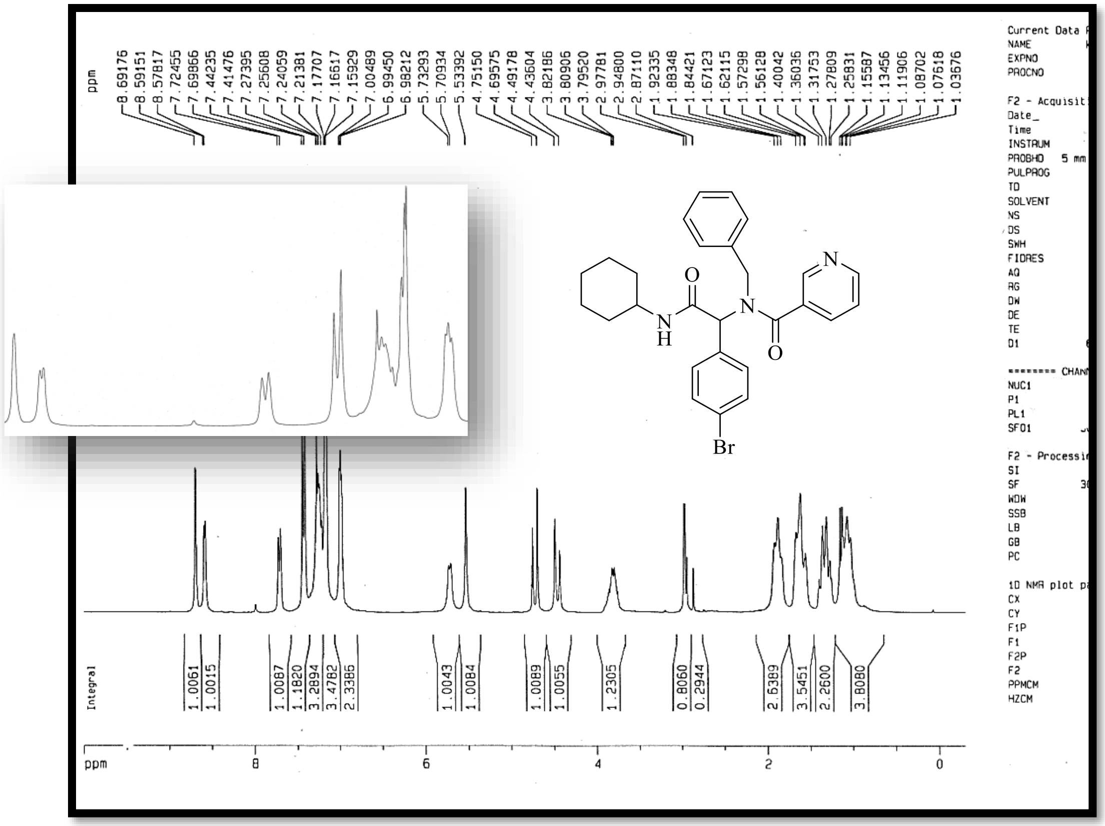

Figure **S50**: ^{1}H-NMR spectra of **7k** (300MHz, $CDCl_3$)

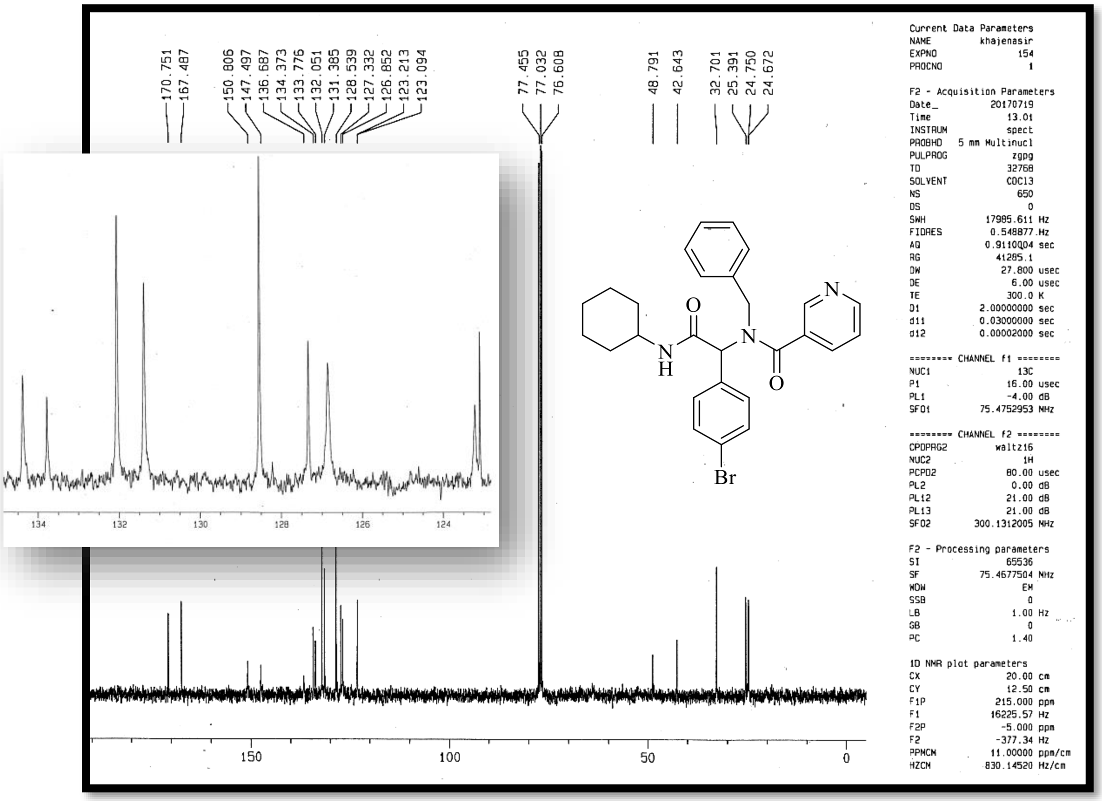

Figure **S51**: ^{13}C-NMR Spectra of **7k** (75 MHz, $CDCl_3$)

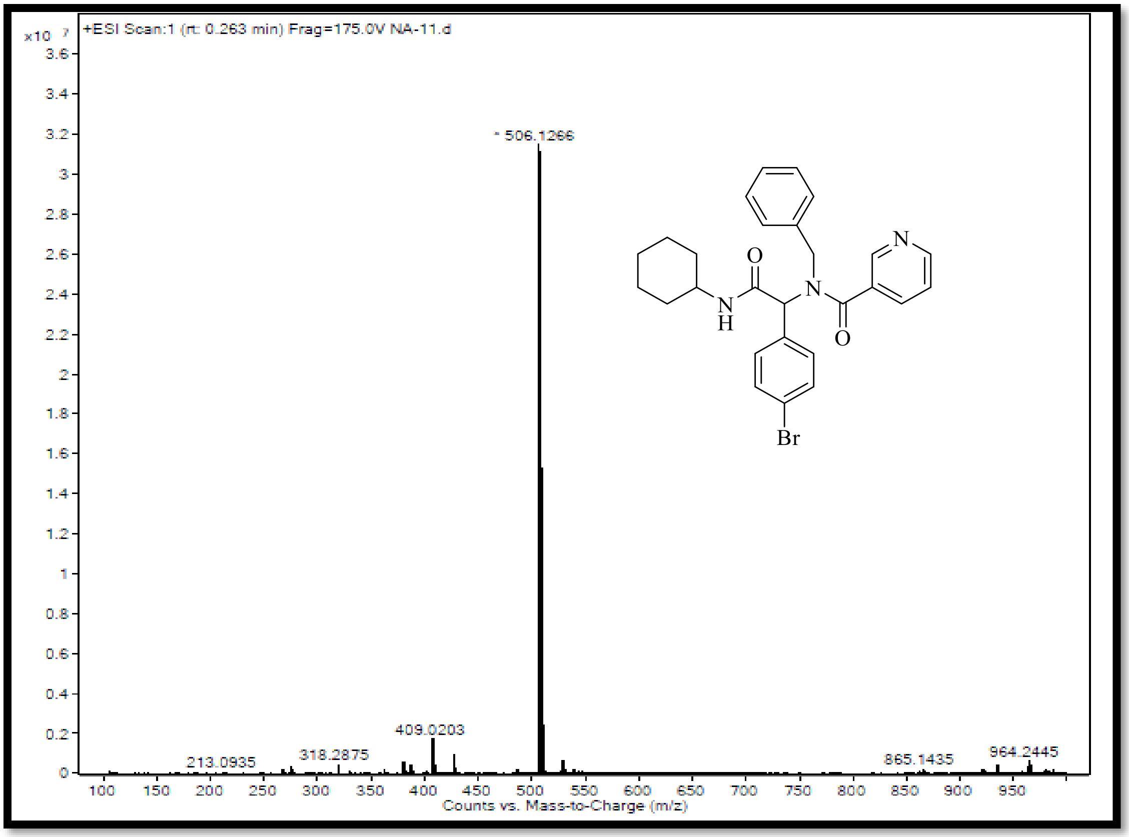

Figure **S52**: HRMS-ESI of **7k** with formula $C_{27}H_{28}BrN_3O_2$ and molecular weight 505.13 g/mol

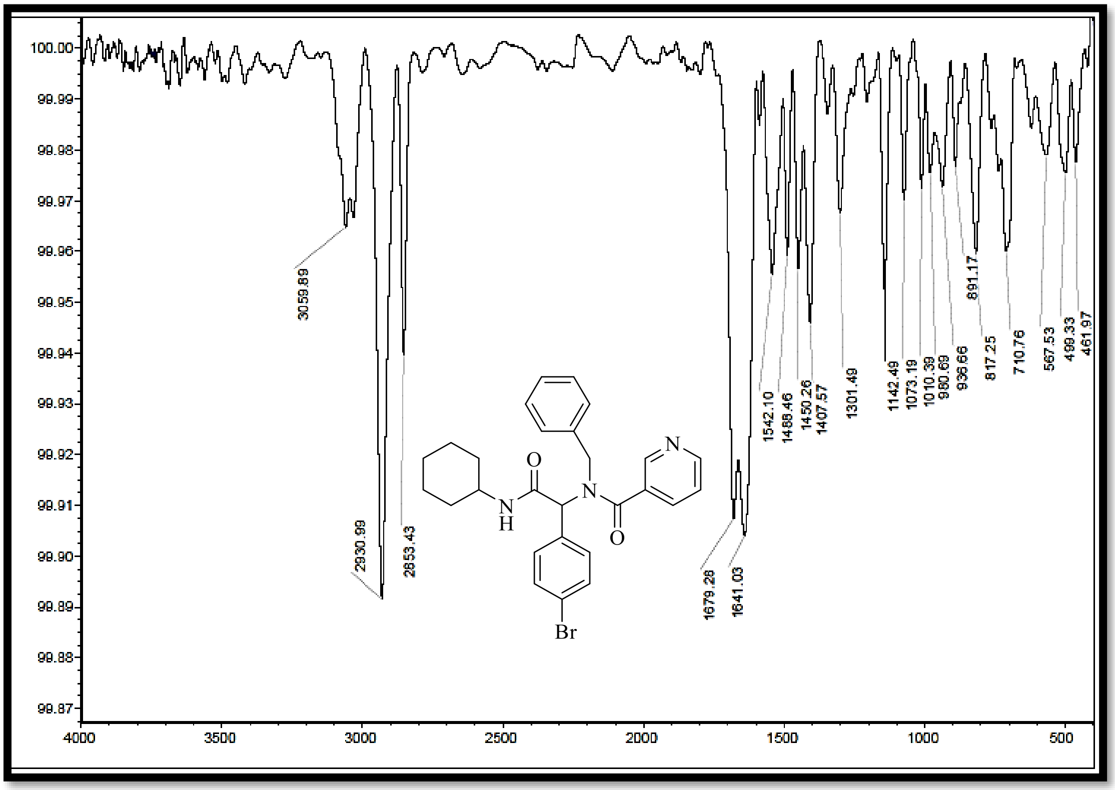

Figure **S53**: IR Spectra of **7k** (KBr, cm-1)

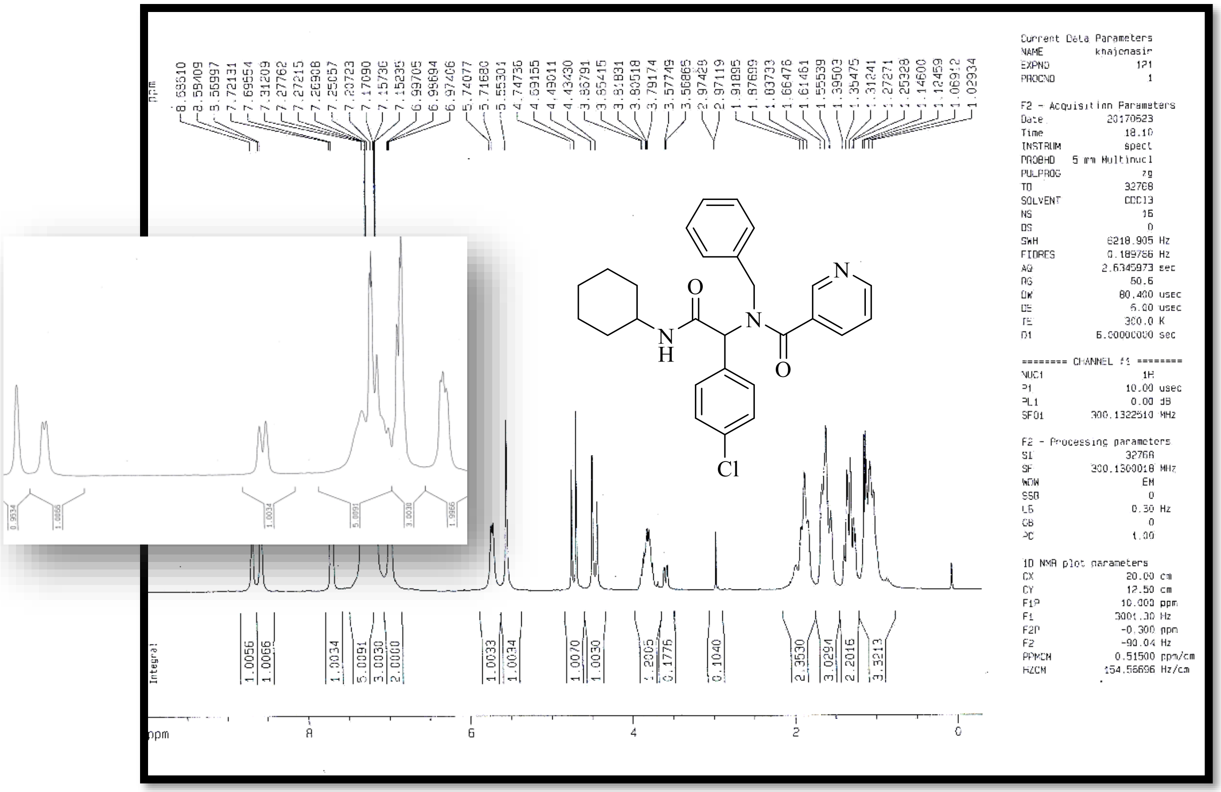

Figure **S54**: ^{1}H-NMR spectra of **7l** (300MHz, $CDCl_3$)

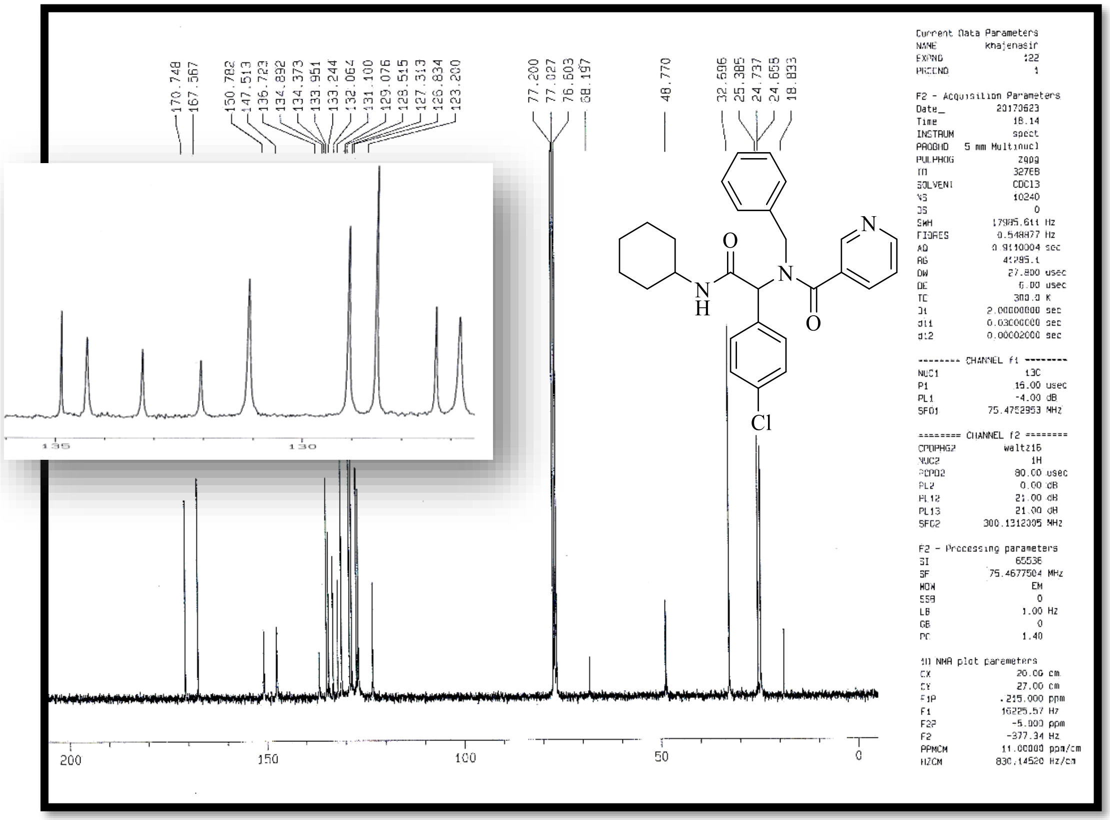

Figure **S55**: ^{13}C-NMR Spectra of **7l** (75 MHz, $CDCl_3$)

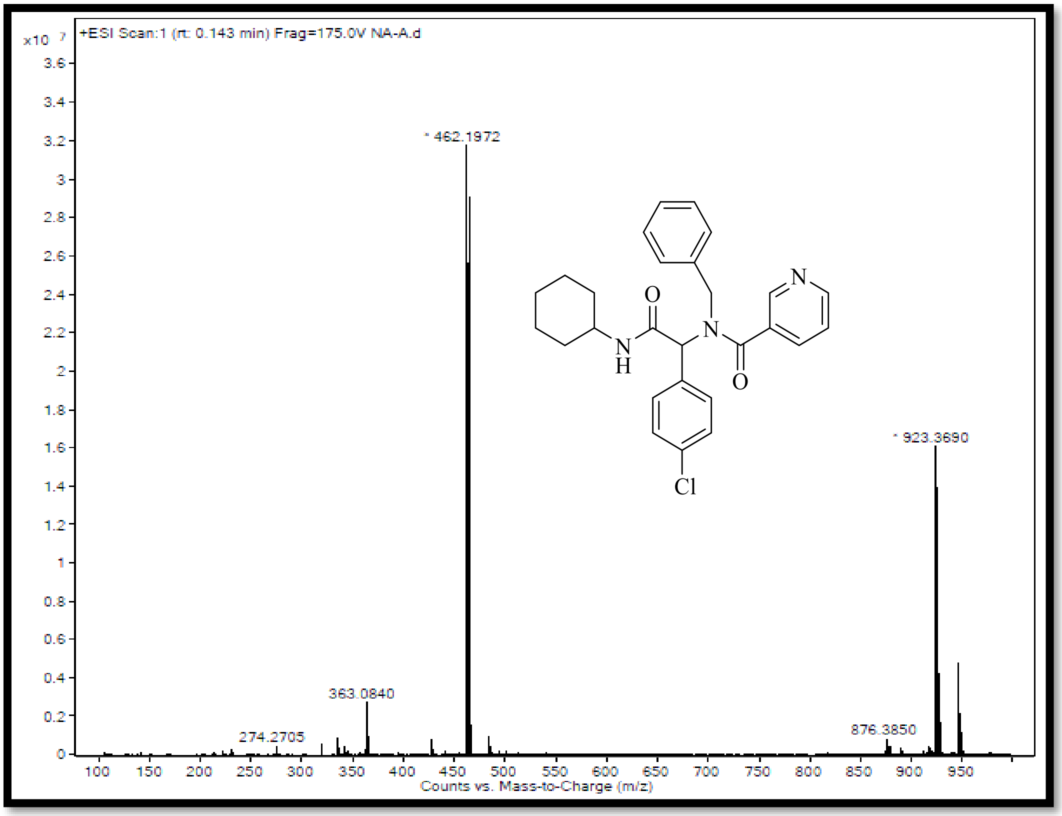

Figure **S46**: HRMS-ESI of **7l** with formula $C_{27}H_{28}ClN_3O_2$ and molecular weight 461.18 g/mol

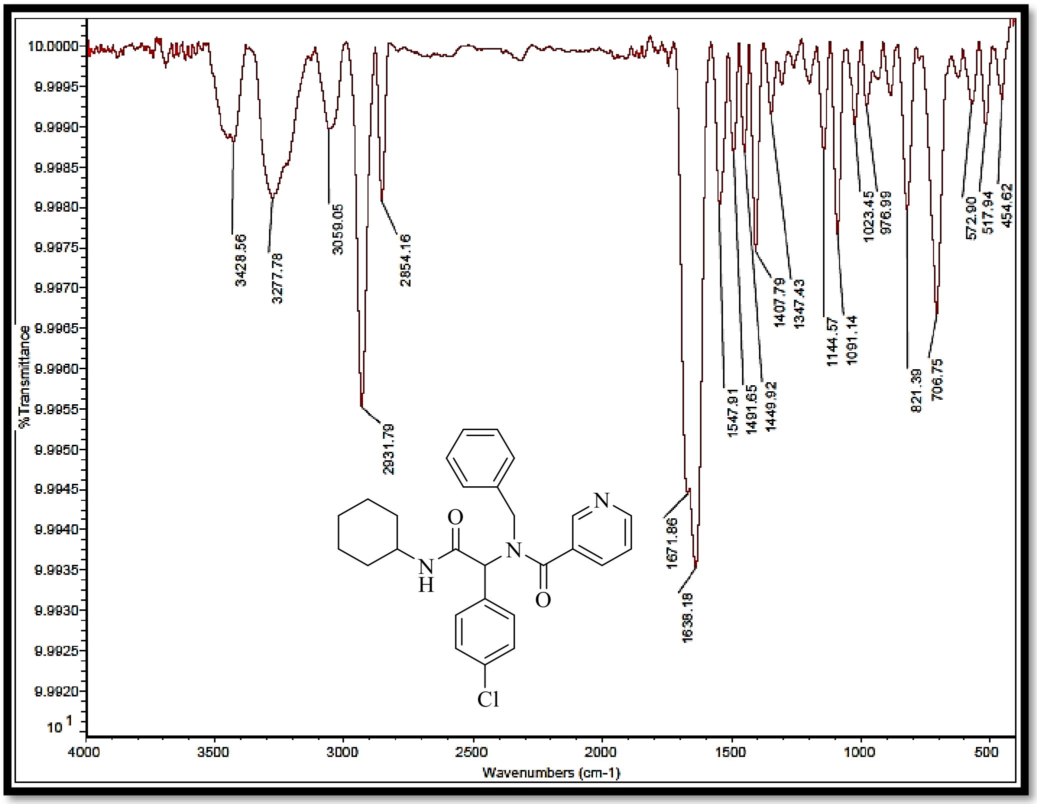

Figure **S57**: IR Spectra of **7l** (KBr, cm-1)

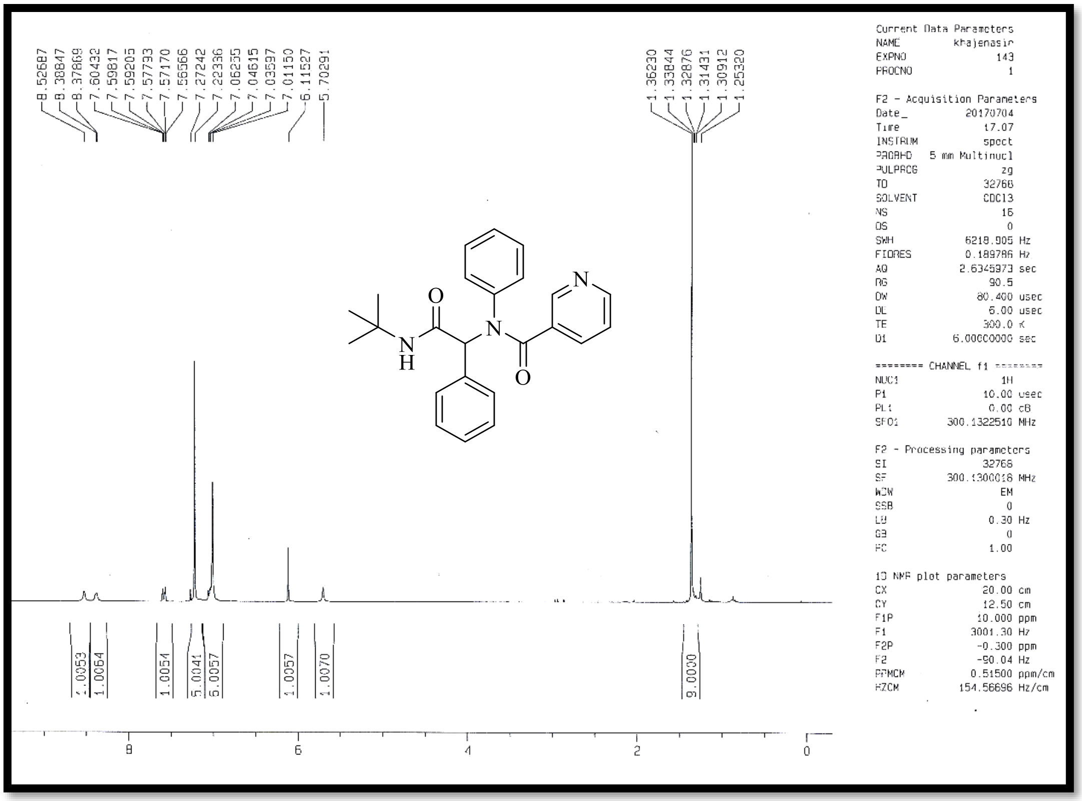

Figure **S58**: ^{1}H-NMR spectra of **7m** (300MHz, $CDCl_3$)

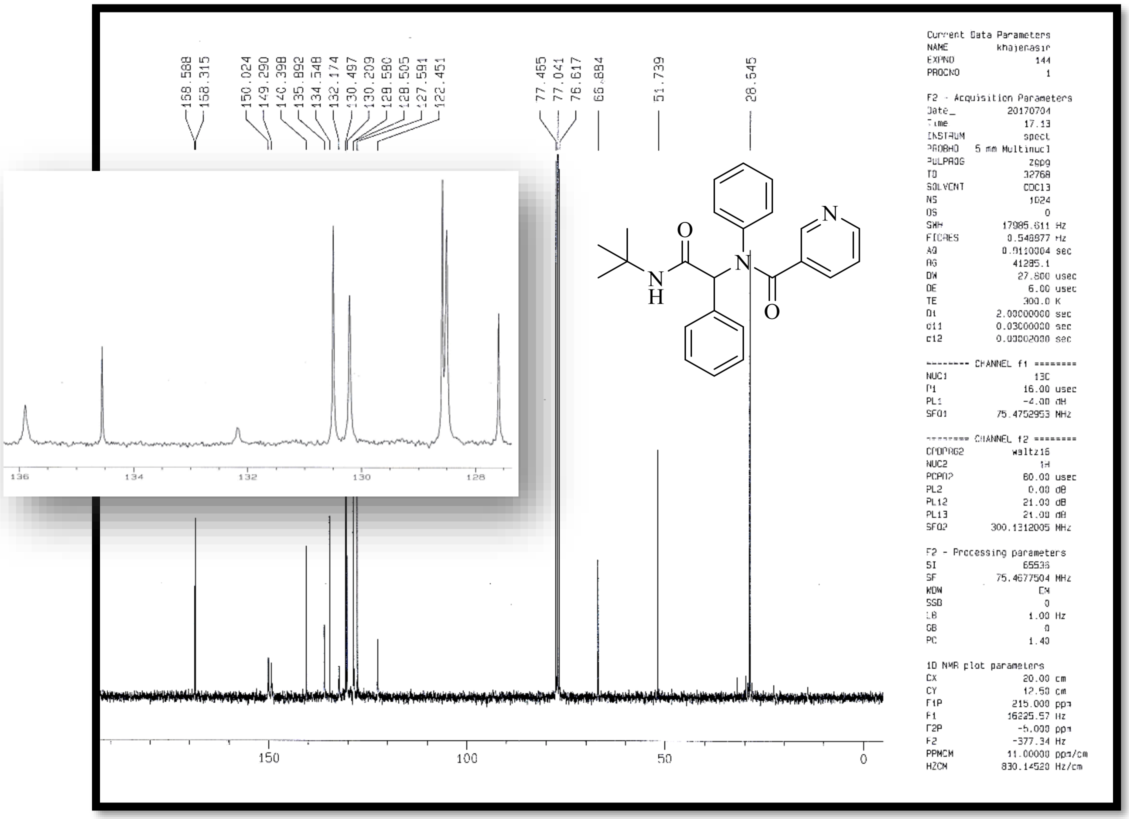

Figure **S59**: ^{13}C-NMR Spectra of **7m** (75 MHz, $CDCl_3$)

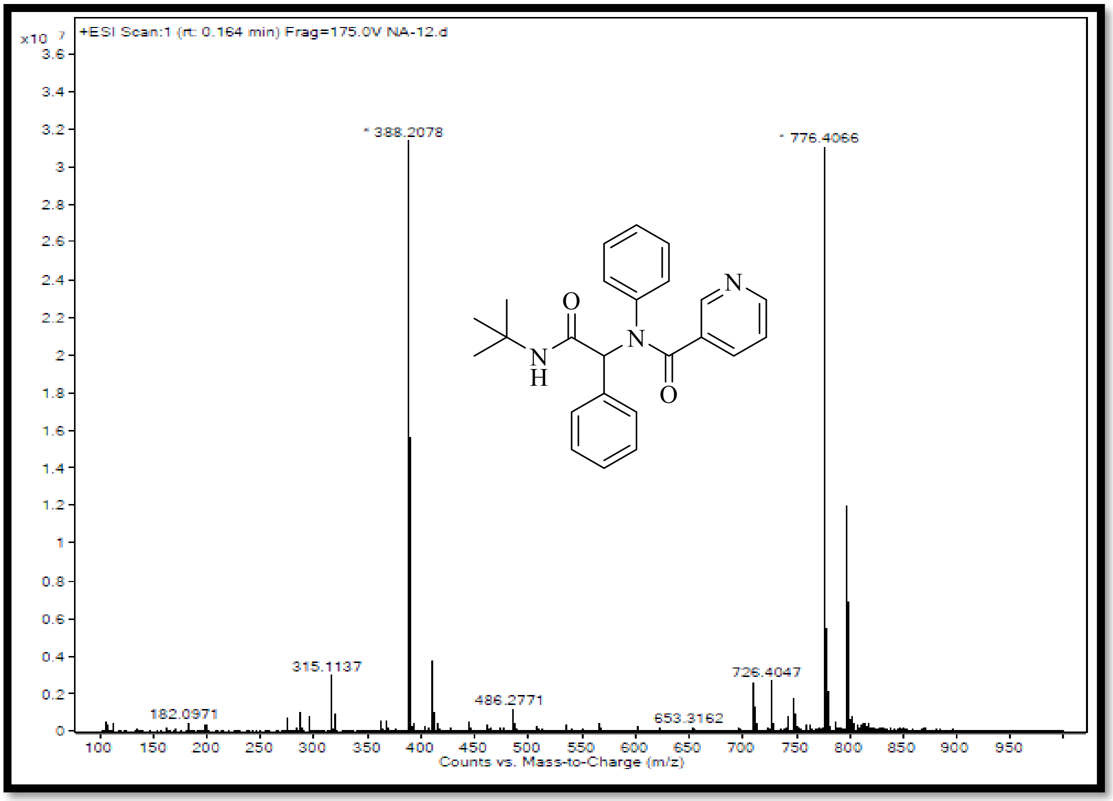

Figure **S60**: HRMS-ESI of **7m** with formula $C_{24}H_{25}N_2O_3$ and molecular weight 387.19 g/mol

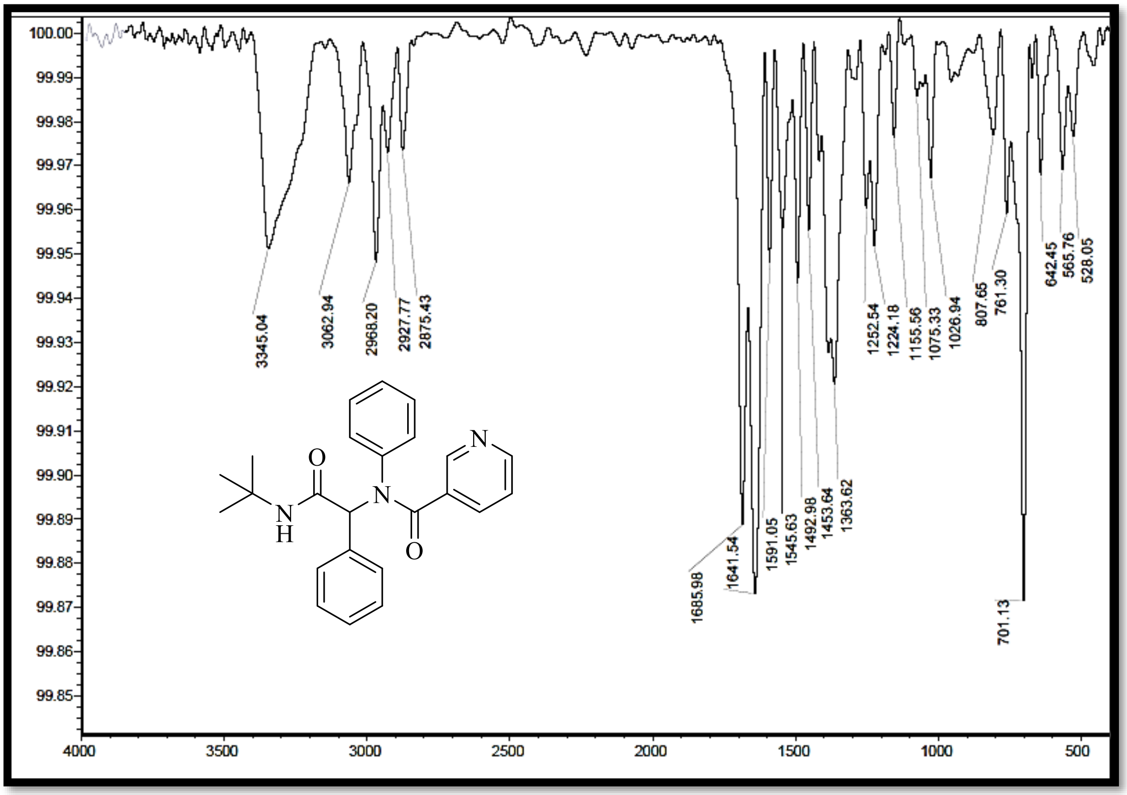

Figure **S61**: IR Spectra of **7m** (KBr, cm^{-1})

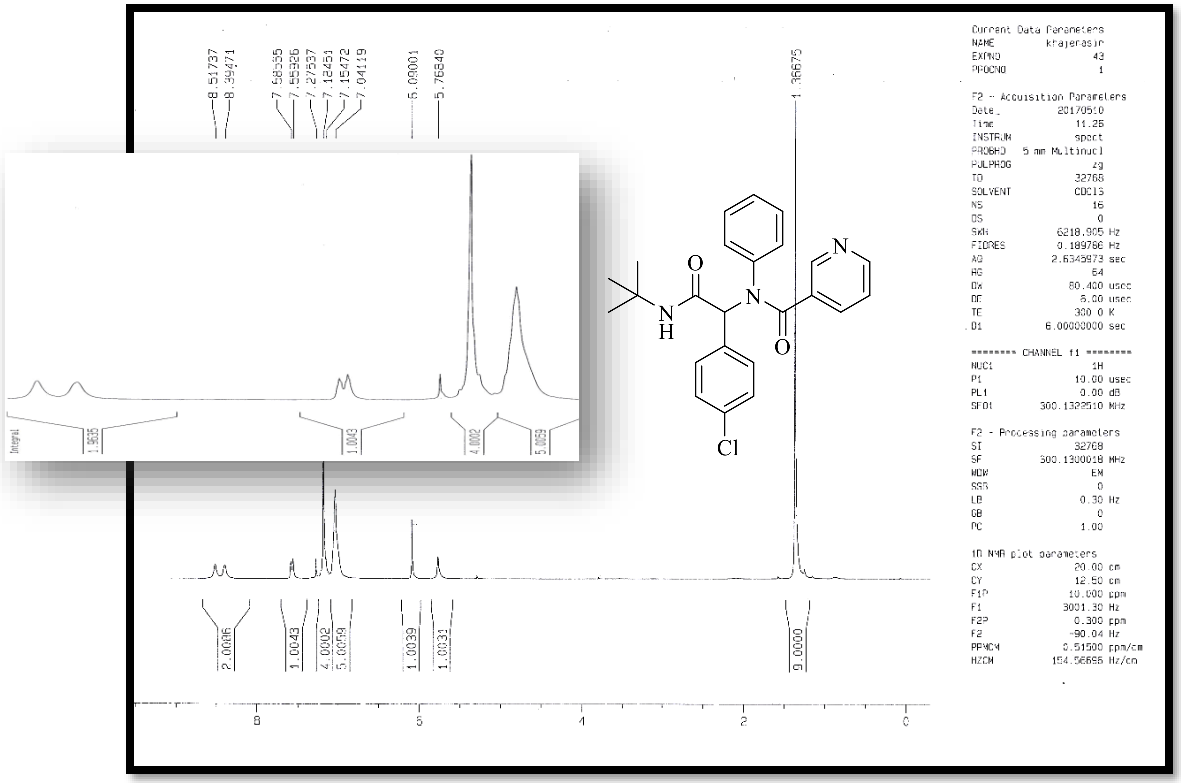

Figure **S62**: ^{1}H-NMR spectra of **7n** (300MHz, $CDCl_3$)

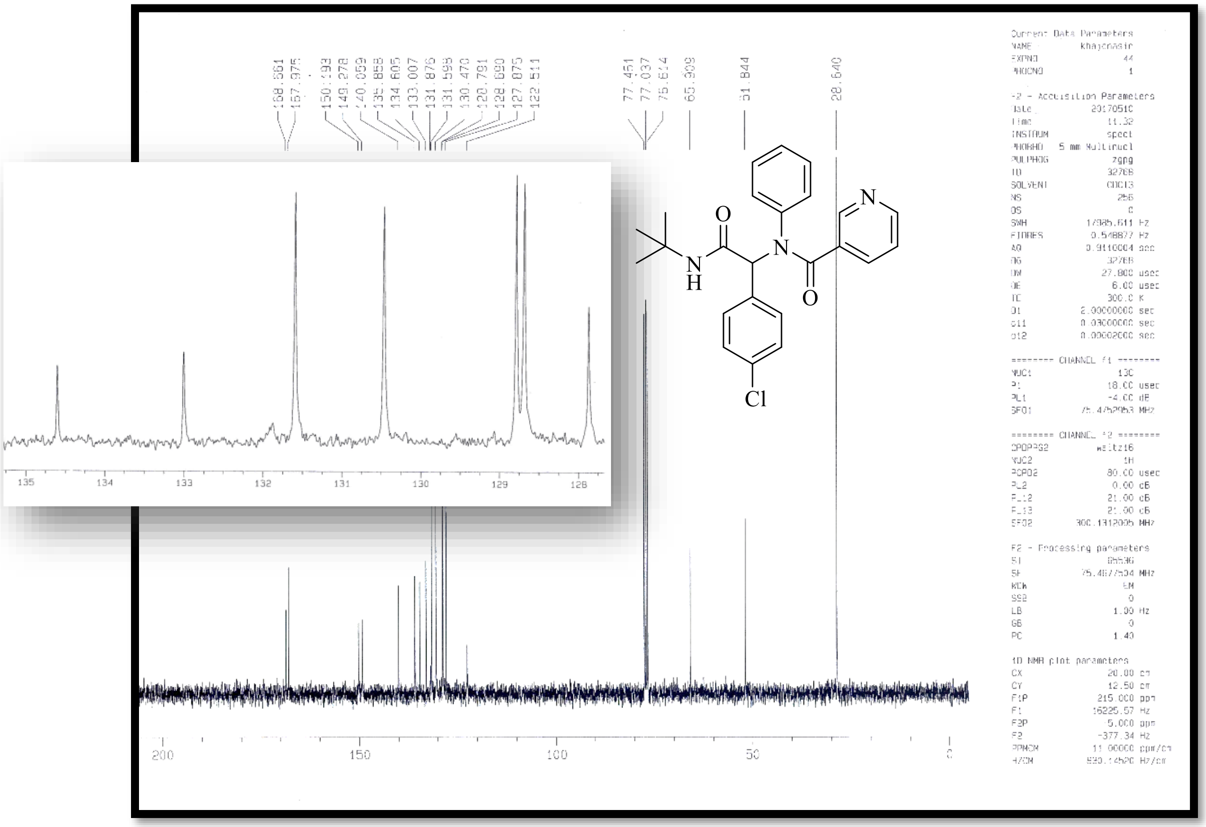

Figure **S63**: ^{13}C-NMR Spectra of **7n** (75 MHz, $CDCl_3$)

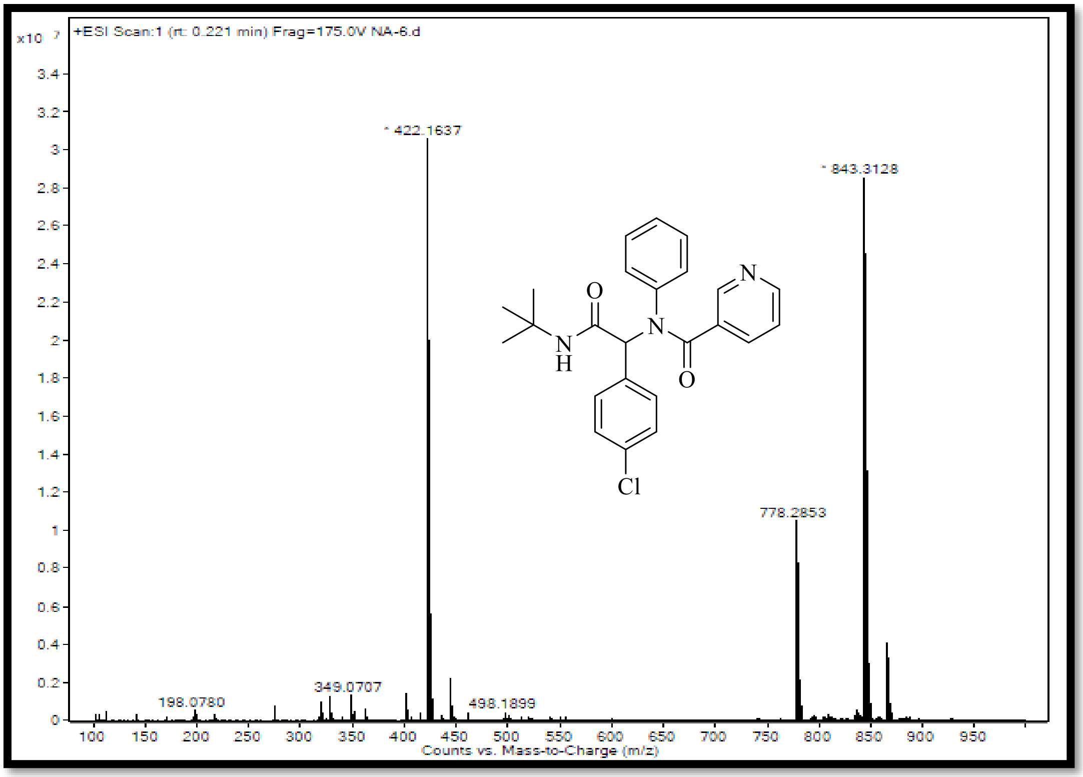

Figure **S64**: HRMS-ESI of **7n** with formula $C_{24}H_{24}ClN_3O_2$ and molecular weight 421.15 g/mol

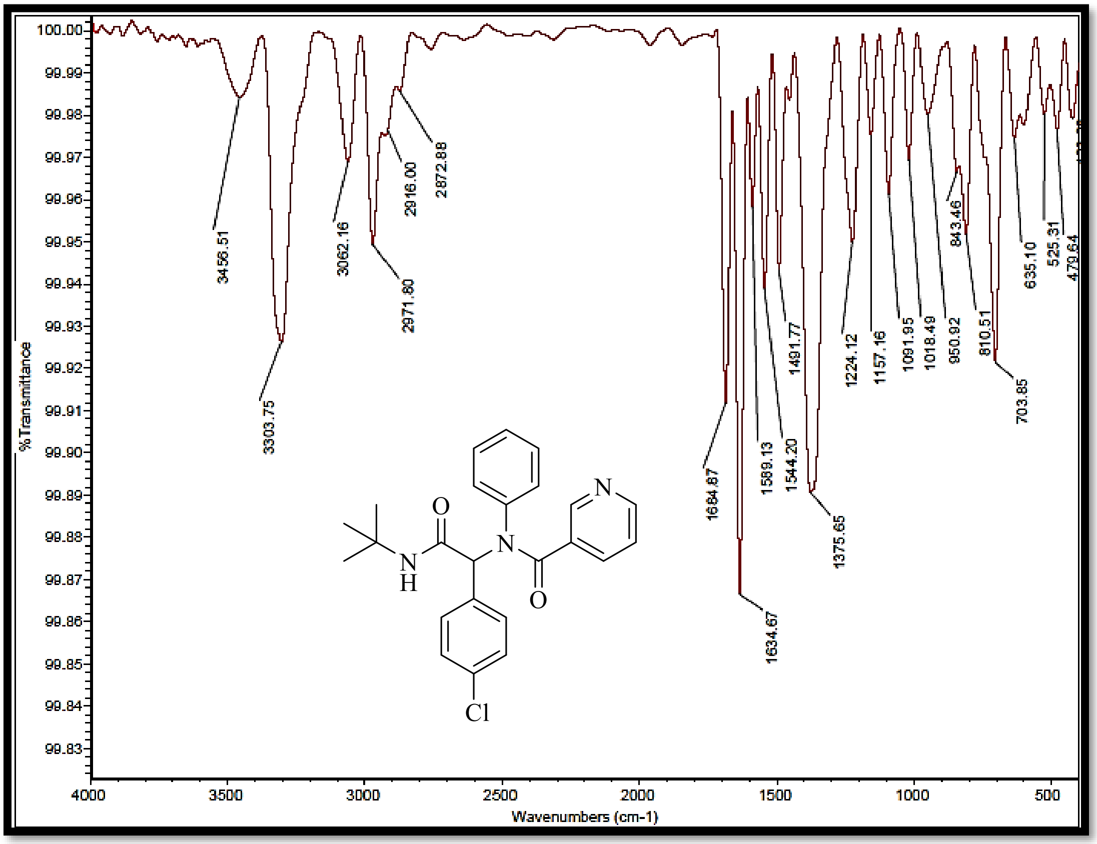

Figure **S65**: IR Spectra of **7n** (KBr, cm^{-1})

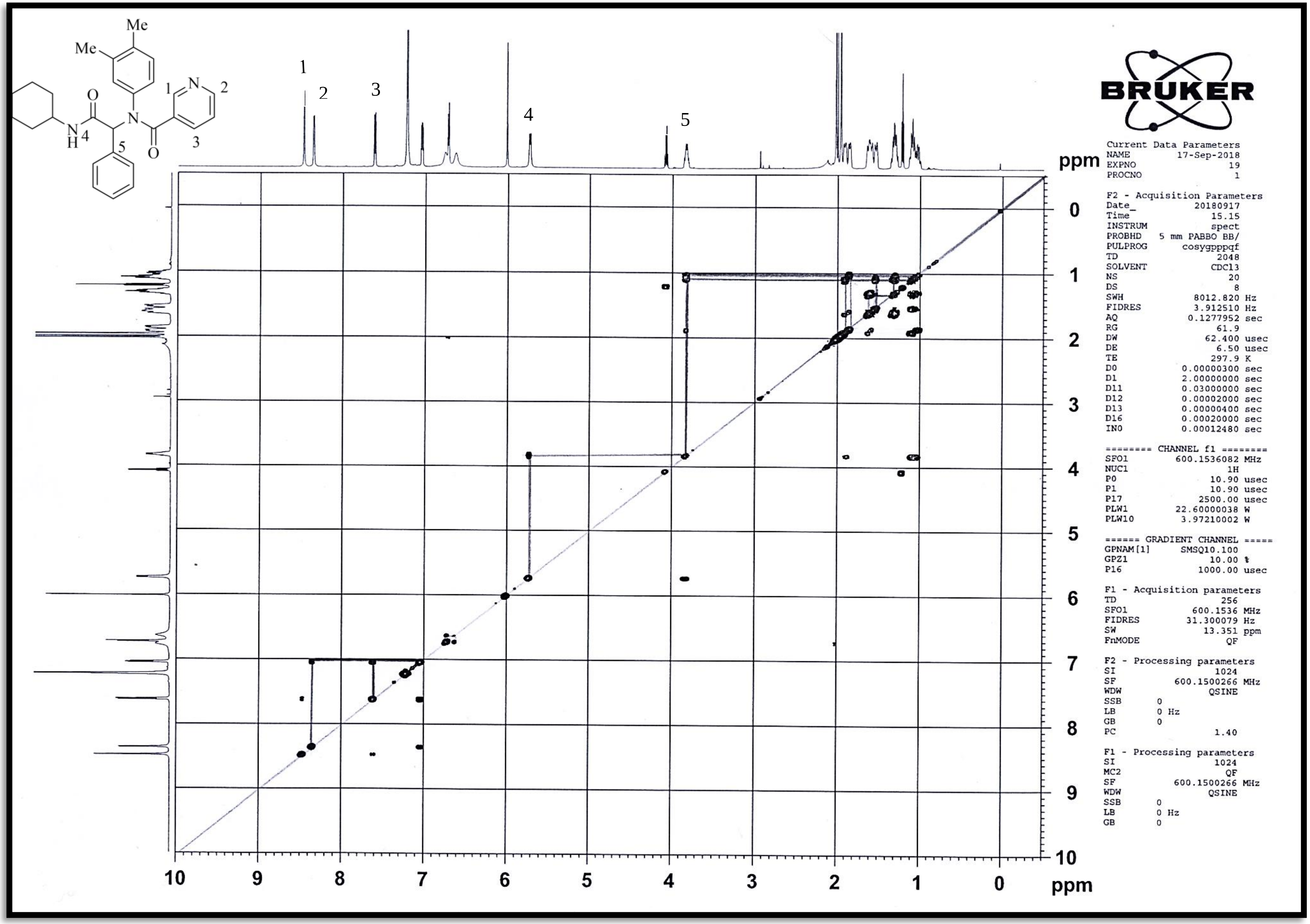

Figure **S66**: GOSYGPSW spectra of **8n** (600MHz, $CDCl_3$)

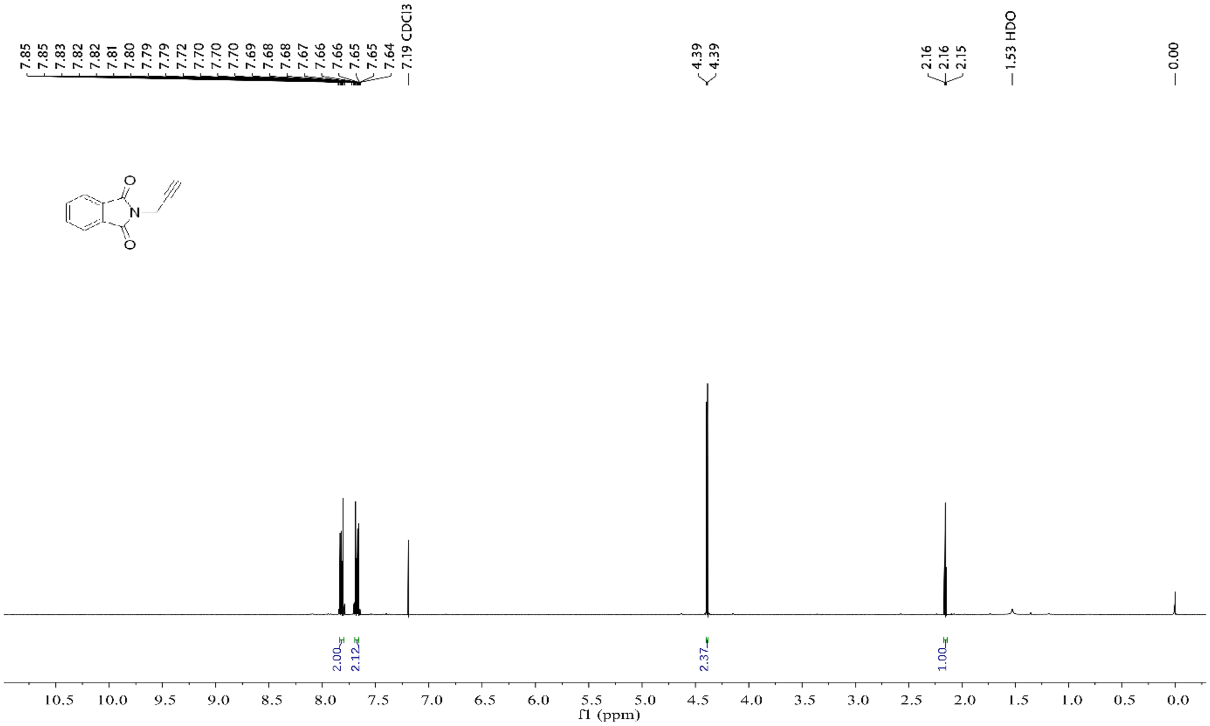

Figure **S67**: ^{1}H NMR spectra (300MHz, $CDCl_3$)

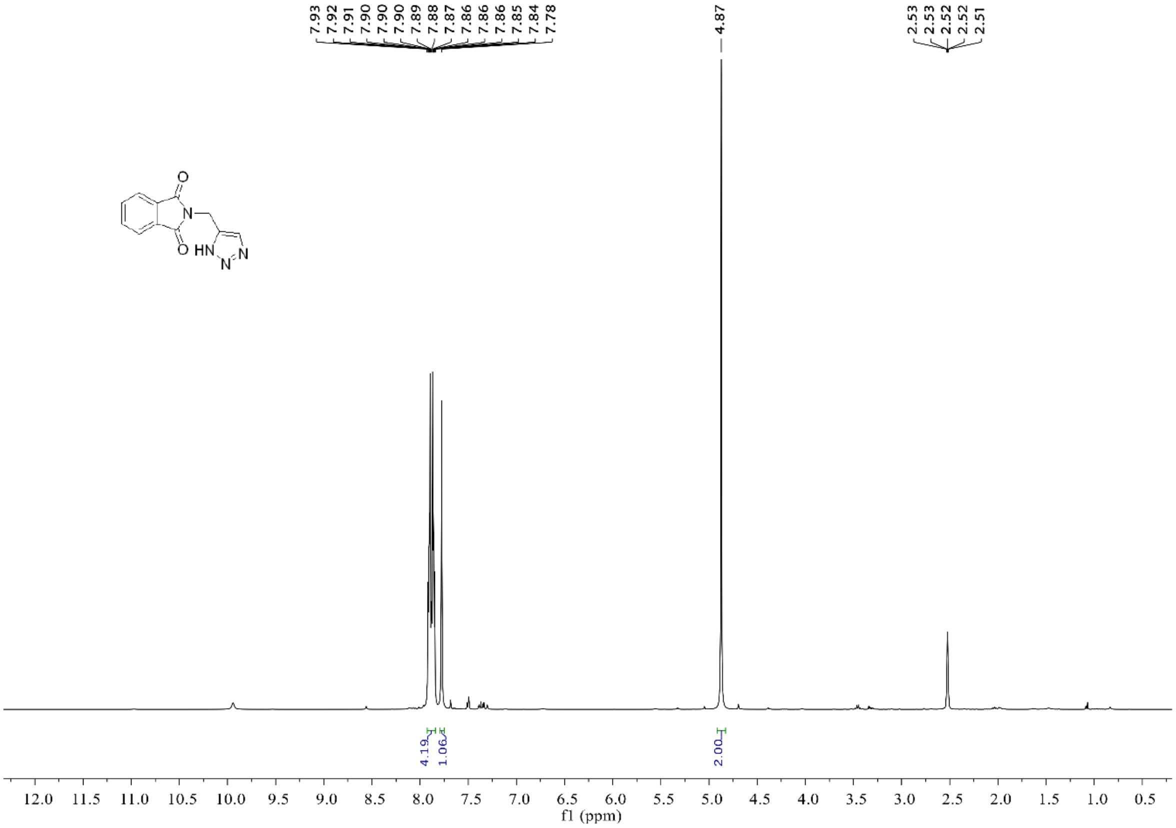

Figure **S68**: ^{1}H-NMR spectra (400MHz, DMSO-d_6)

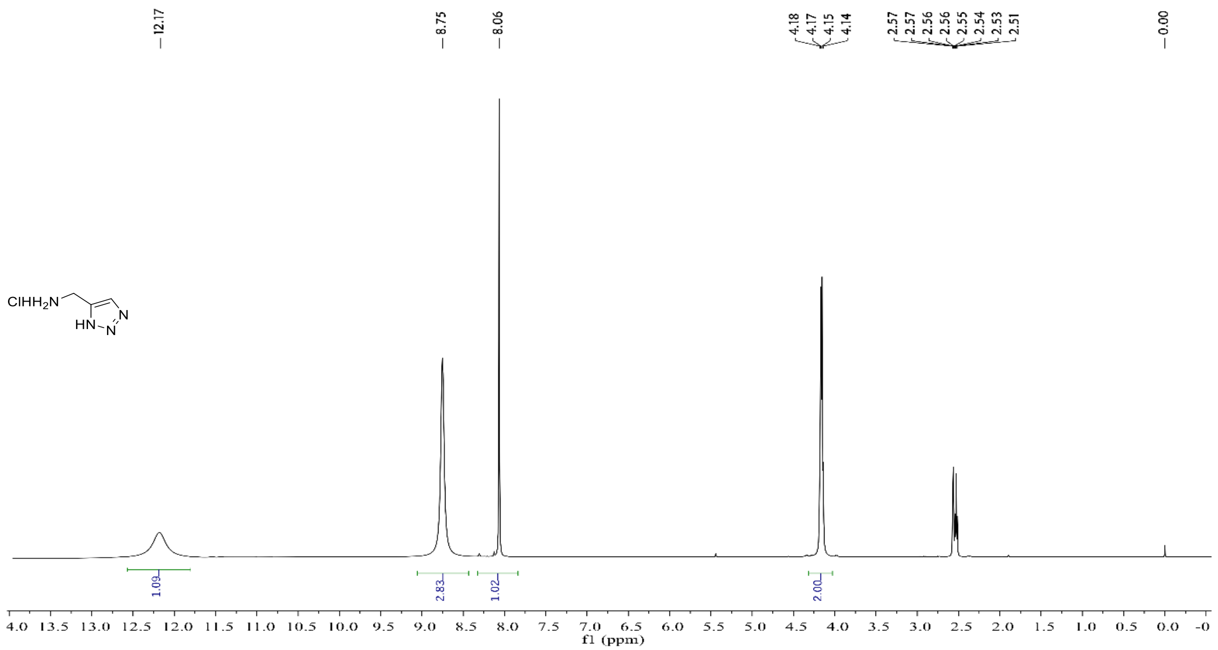

Figure **69**: ^{1}H-NMR spectra (400MHz, DMSO-d_6)

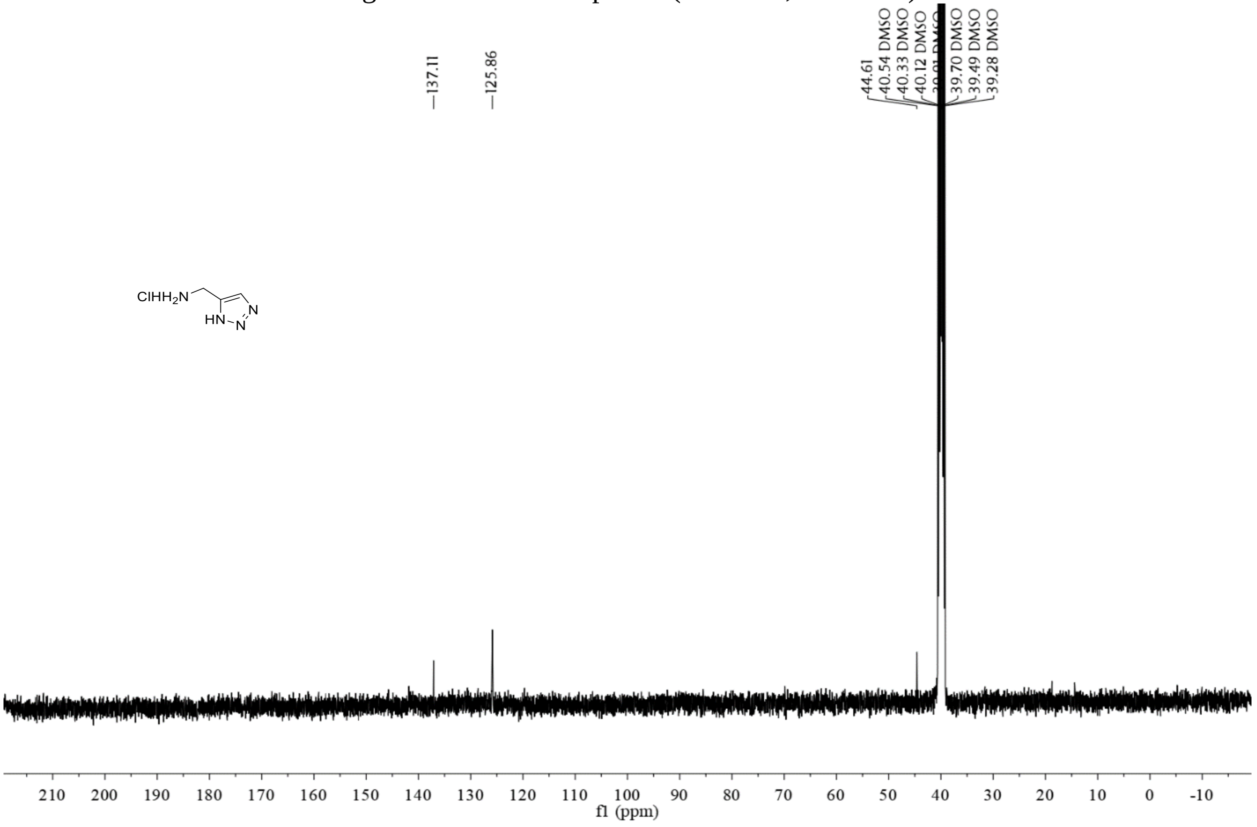

Figure **70**: ^{13}C-NMR spectra (101MHz, DMSO-d_6)
